# A Novel Dual Organocatalyst for the Asymmetric Pinder Reaction and a Mechanistic Proposal Consistent with the Isoinversion Effect Thereof

**DOI:** 10.3390/molecules26216398

**Published:** 2021-10-22

**Authors:** Fotini Moschona, Athena Vagena, Veroniki P. Vidali, Gerasimos Rassias

**Affiliations:** 1 Department of Chemistry, University of Patras, 26504 Patra, Greece; fmoschona@windowslive.com (F.M.); athinaupatrwn@gmail.com (A.V.); 2 NCSR “Demokritos”, Institute of Nanoscience and Nanotechnology, Patr. Grigoriou and Neapoleos 27, 153 41 Athens, Greece; v.vidali@inn.demokritos.gr

**Keywords:** pinder reaction, castagnolli–cushman reaction, asymmetric organocatalysis, chiral thiourea-BiMAH catalysts, isoinversion temperature

## Abstract

In general, the Pinder reaction concerns the reaction between an enolisable anhydride and an aldehyde proceeding initially through a Knoevenagel reaction followed by the ring closing process generating lactones with at least two chiral centers. These scaffolds are frequently present in natural products and synthetic bioactive molecules, hence it has attracted intense interest in organic synthesis and medicinal chemistry, particularly with respect to controlling the diastereo- and enantioselectivity. To the best of our knowledge, there has been only one attempt prior to this work towards the development of a catalytic enantioselective Pinder reaction. In our approach, we designed, synthesized, and tested dual chiral organocatalysts by combining BIMAH amines, (2-(α-(alkyl)methanamine)-1H-benzimidazoles, and a Lewis acid motif, such as squaramides, ureas and thioureas. The optimum catalyst was the derivative of isopropyl BIMAH bearing a bis(3,5-trifluoromethyl) thiourea, which afforded the Pinder products from various aromatic aldehydes with diastereomeric ratio >98:2 and enatioselectivity up to 92 ee%. Interestingly, the enantioselectivity of this catalyzed process is increased at higher concentrations and exhibits an isoinversion effect, namely an inverted "U" shaped dependency with respect to the temperature. Mechanistically, these features, point to a transition state involving an entropy-favored heterodimer interaction between a catalyst/anhydride and a catalyst/aldehyde complex when all other processes leading to this are much faster in comparison above the isoinversion temperature.

## 1. Introduction

**Scheme 1:**
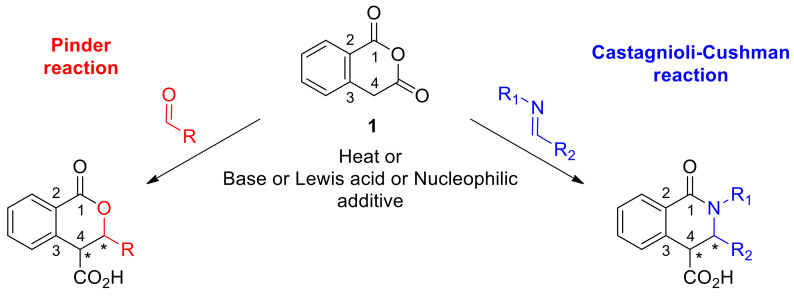
The established reactions of Pinder and Castagnoli–Cushman.

The Pinder reaction has its roots in the Perkin reaction dating back to 1877, where an anhydride was first shown to defy its electrophilic nature and react as a nucleophile instead; at elevated temperatures, acetic anhydride reacted with aldehydes to give the aldol condensation product [1]. In the Perkin–Fittig development, it was demonstrated that with succinic anhydride, the initial aldol product could ring-close to afford the corresponding γ-lactone; although, when heated further, decarboxylation and dehydration occurred to yield the Perkin product [2]. In general, succinic anhydride reacts sluggishly while the 2-aryl substituted analogues exhibit enhanced enolization potential and have proved more productive substrates for the Perkin–Fittig reaction. In 1958, Pinder first reported the reaction of the more readily enolisable homophthalic anhydride (**1**, Scheme 1, pKa ~ 8.2) with aldehydes in the presence of a base to give lactones, or more precisely, 3,4-disubstituted isochroman-1-ones [3].

**Scheme 2:**
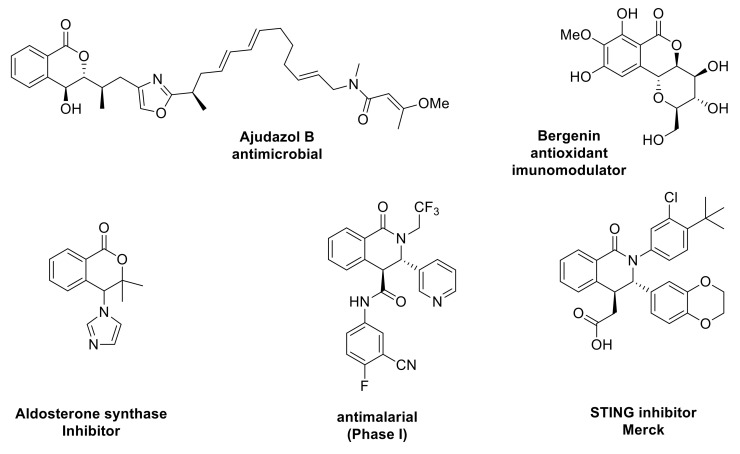
Natural products and drug molecules containing the scaffold of Pinder and Castagnoli–Cushman reaction products.

Forty years later, Gesquire reported the use of BF_3_^.^Et_2_O for the Pinder reaction, although no information was provided on the stereochemical outcome [4]. Bogdanov and Palamareva later disclosed the use of DMAP as an efficient promoter to afford 3,4-disubstitutedisochroman-1-ones with good diastereocontrol (40–90 de%) favoring the trans isomer [5]. The aza-variant of the Pinder reaction was reported in 1969 and is commonly known as the Castagnoli–Cushman reaction (Scheme 1) [6,7,8,9,10]. Recent works by the Knapp and Shaw groups exemplified that, like the Pinder reaction, the Castagnoli–Cushman reaction is also promoted efficiently by nucleophiles and bases such as NMI (M-methyl imidazole) and TMG (tetramethyl guanidine), respectively [11,12,13]. Both Pinder and Castagnoli–Cushman reactions give rise to heterocycles with two adjacent stereogenic centres, a motif that is frequently present among both natural products and synthetic bioactive compounds (Scheme 2) [13,14,15,16,17,18,19,20].

**Scheme 3:**
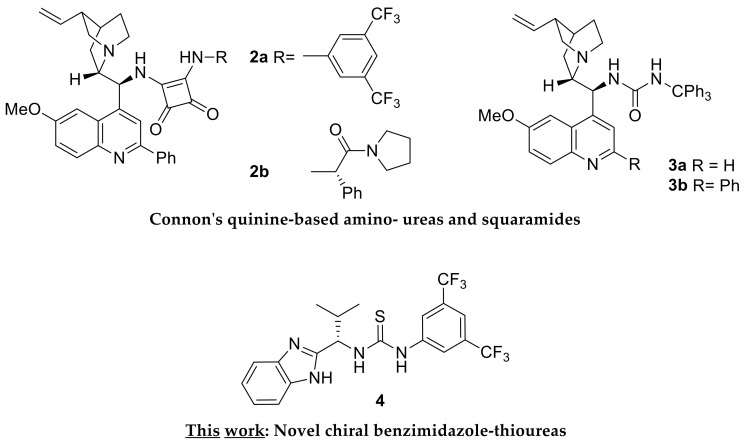
Previously reported and presently described chiral organocatalysts for the asymmetric Pinder reaction.

Surprisingly, it was not until 2012 that the first asymmetric version of the Pinder reaction appeared by Connon who employed the squaramide organocatalyst **2a** derived from a modified quinine scaffold (Scheme 3) [21]. At 5 mol%, −15 °C, in TBME, typically over 8 hours, this catalyst allowed the formation of the dihydroisocoumarine/isochroman-1-one products of homophthalic anhydrides and aliphatic and aromatic aldehydes in high yields (78–98%) and enantioselectivity (90–99 ee%). The diastereocontrol was equally high with aromatic aldehydes (80–94 ee%), albeit lower with aliphatic substrates, favoring the trans isomer in all cases. By developing related organocatalysts **2b**, **3a** and **3b**, the Connon group expanded the scope of the Pinder reaction to encompass the kinetic resolution of chiral aldehydes, the selective formation of cis adducts with aldehydes, and the use of ketones as substrates, respectively [22,23,24,25]. These catalysts are suggested to operate initially by deprotonation of homophthalic anhydride by the basic nitrogen with the resulting ammonium species and the anhydride enolate being in turn held though hydrogen bonds by the Lewis acidic squaramide or urea module of the organocatalyst. The aldehyde is then activated through a hydrogen bond to the ammonium moiety of the alkaloid, thus facilitating an essentially intramolecular nucleophilic attack by the enolate anion. The resulting alkoxide then attacks the benzoyl-type anhydride carbonyl, precipitating the formation of the lactone.

We envisaged that the process would be further facilitated or alternatively promoted by a nucleophilic catalyst capable of attacking the anhydride moiety, thus producing an active acylating agent towards the alcohol intermediate, resulting from the initial attack of the enolate to the aldehyde. Retaining a Lewis acidic catalyst component in the form of (thio)urea or squaramide would ensure the activation of the aldehyde and/or the complexation with the enolate species. Inspired by the successful application of nucleophilic catalysts such as DMAP and NMI in the racemic Pinder and Castagnoli–Cushman processes, respectively, we designed and synthesized a series of chiral benzimidazole-thioureas from which organocatalyst **4** (Scheme 3) emerged as a particularly efficient catalyst that promotes the Pinder reaction with reasonably good diastereo- and enantioselectivity.

## 2. Results and Discussion

### 2.1. Exploring Conditions for Promoting and Suppressing the Racemic Reaction

At the onset of any catalytic asymmetric reaction development, two sets of reaction conditions need to be established. The first relates to a procedure for efficiently generating the racemic products in order to develop a chiral HPLC or alternative analytical method for the determination of the enantiomeric ratio of the products subsequently obtained via asymmetric catalysis. The second set of conditions concerns the maximum suppression, ideally complete silencing, of the background racemic reaction, thus ensuring that any conversion to the product(s) upon addition of the chiral catalyst will arise exclusively though the mediator, hence the enantioselectivity and overall efficiency of the process would not be confounded by alternative pathways.

**Scheme 4:**
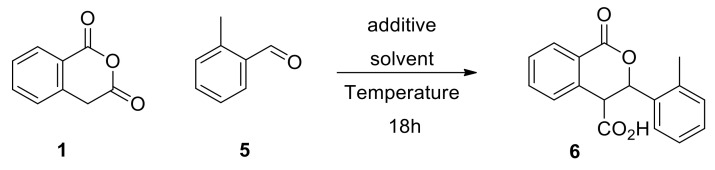
Model Pinder reaction for exploring conditions of maximum and minimum conversion.

In this context, we used the reaction between homophthalic anhydride (**1**, 1 eq) and *o-*methyl benzaldehyde (**5**, 1.1 eq) as the model Pinder reaction to explore initially potential conditions and mediators for the generation of racemic **6** (Scheme 4). The reaction was tested at various temperatures in ethereal solvents used routinely in the Pinder reaction in the presence and absence of the nucleophilic/mildly basic NMI (p*K*aH 7.4), or the much more basic and non-nucleophilic DIPEA (p*K*aH 10.7). The reaction with the latter was more efficient than with NMI in all cases, yet the reaction could not be suppressed in either case even at −10 °C (Table 1). These results meant that the DIPEA process was ideal for generating our racemic products almost quantitatively and that their purification could be achieved easily by crystallization after standard work-up without the need for chromatography. More importantly, we demonstrated that the Pinder reaction could be mediated by a nucleophilic catalyst such as NMI suggesting that incorporation of similar moieties in a chiral catalyst would benefit the asymmetric Pinder reaction, thus allowing us to explore organocatalyst modules other than the classical chinqona bases. We also achieved our objective in determining reaction conditions that suppress the racemic transformation as, in the absence of a promoter, no reaction takes place in *tert-*butyl methyl ether (TBME) either at 0 or −10 °C after 18 hours (Table 1).

In order to ensure this was not due to mass transfer effects due to the low solubility of homophthalic anhydride in cold TBME, we also tested the reaction in cyclopentyl methyl ether (CPME) in which the said substrate is much more soluble. Equally, no reaction was observed in this case either at 0 or −10 °C (Table 1). We chose not to try lower temperatures since our scope was to establish practical conditions for our catalyzed process (typical lowest process temperature used in the industry is −20 °C) and also because Connon had already shown that high ee’s could be obtained at −15 °C with catalyst **2a** [21].

### 2.2. Exploring the Scope of the Aldehyde Substrate

**Scheme 5:**
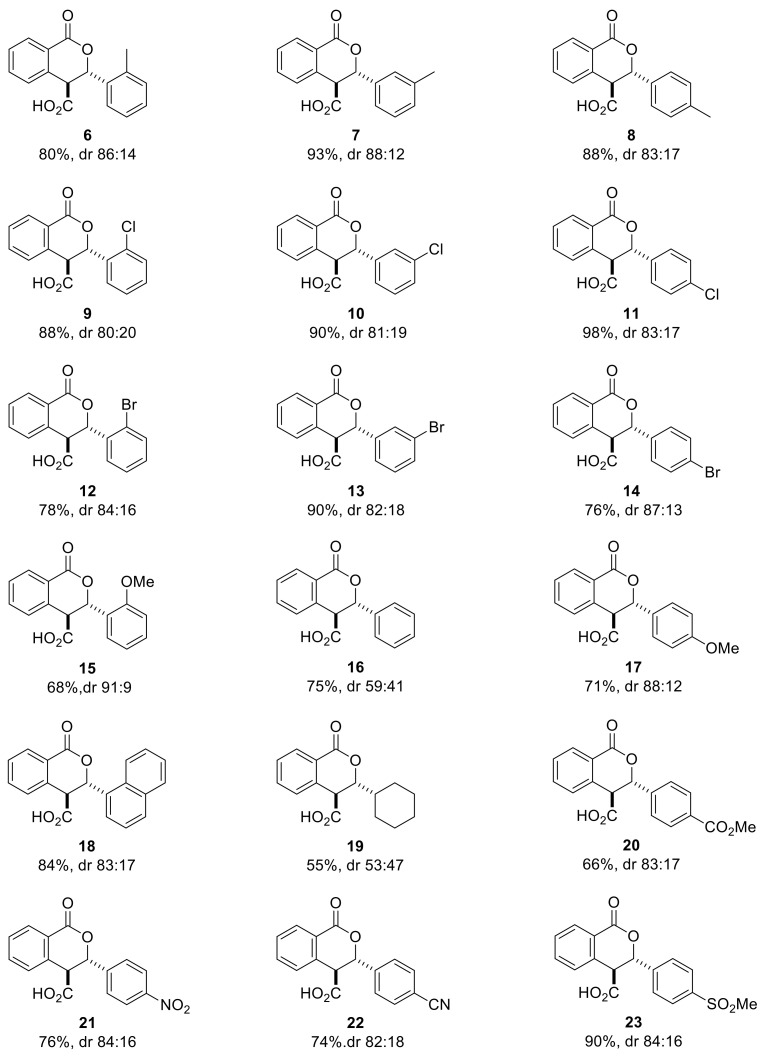
Pinder reaction with various substituted aldehydes under the optimized conditions for the racemic reaction (THF, DIPEA, rt, 18 h).

Next, we used our optimized racemic conditions (THF, DIPEA, rt, 18 h) to perform the Pinder reaction with homophthalic anhydride and aromatic aldehydes of various stereoelectronic properties including the aliphatic cyclohexyl carbaldehyde (Scheme 5).

**Scheme 6:**
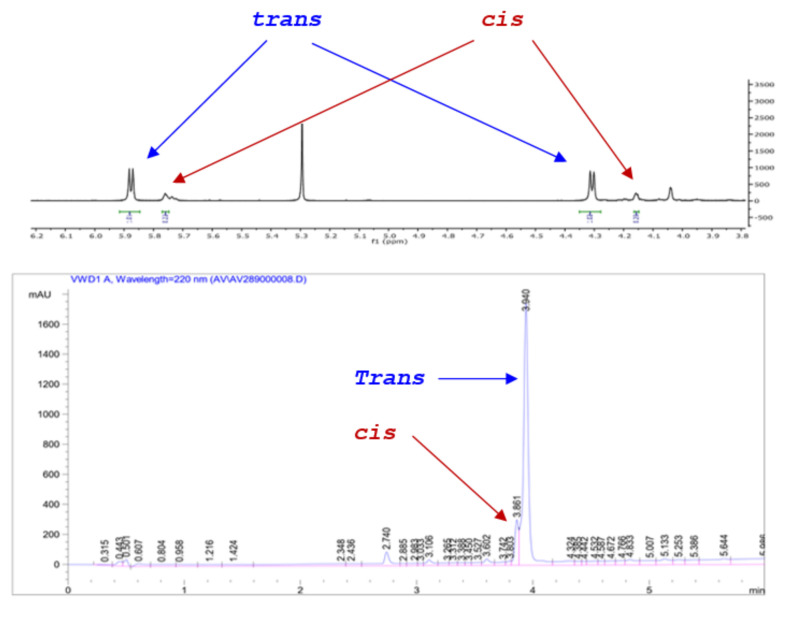
HPLC and ^1^H NMR of crude product **13** demonstrating consistent assessment of *cis/trans* isomer ratios.

All reactions were monitored by HPLC and in most cases the *cis* and *trans* isomers could be discerned and their ratio quantified. In these cases, the *cis*/*trans* ratio in the reaction mixture did not appear to change at prolonged reaction times. More importantly, the HPLC diastereomeric ratio matched exactly that determined by ^1^HNMR (). For example, the dr for **13** was determined at 82:18 by HPLC (favoring the *trans* isomer) during the reaction and after work up and the same value was established by ^1^HNMR for the crude product *via* the *J* values of the *cis* and *trans* ring protons (Scheme 6). As expected, there is no difference in the absorbance between the *cis*/*trans* isomers and the diastereoselectivity of the process could be checked rapidly by HPLC, without applying Response Factor corrections, as the Pinder reaction proceeds.

With the exception of the moderate yield (55%) and negligible diastereoselectivity (dr 53:47) in the Pinder product of cyclohexyl carbaldehyde, all aromatic aldehydes gave high conversions (70–98% yield) and good diastereoselectivities (dr 80:20–91:9); the odd one out being the benzaldehyde adduct with dr 59:41. Nevertheless, in all cases, the *trans* isomer was invariably the predominant one. Isolation by crystallization from hexane and DCM or TBME or ether (1:1 to 6:1 depending on the product) cleanly afforded the *trans* isomer in all cases, indicating a great difference in solubility from the *cis* isomers (^1^HNMR (Supplementary Materials). These results from the DIPEA-promoted racemic/uncontrolled reaction set a reference point for subsequently assessing whether our chiral catalysts enhance, oppose, or override the intrinsic stereochemical preference of the uncatalyzed Pinder reaction.

**Scheme 7:**
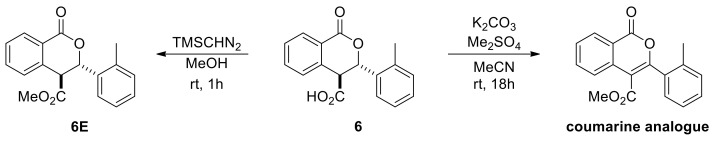
Esterification attempts of the Pinder acid products for analytical purposes.

The racemic products were next used in the development of appropriate chiral HPLC methods that would help establish any asymmetric induction imparted by chiral catalysts evaluated in the subsequent part of the investigation. The low solubility of the acid derivatives was beneficial for their isolation and purification, nevertheless it hindered sample preparation of isopropanol/hexane mixtures (typical mobile phase for normal phase chiral HPLC columns). Attempts to develop reverse or normal phase methods was not met with success, except for only three cases (6, 7 and 9). This is perhaps the reason why in the Connon and Seidel works on asymmetric Pinder and Castagnoli–Cushman reactions respectively, all acid derivatives were converted to their methyl ester analogues prior to determining the ee’s by chiral HPLC. Initially, we attempted the esterification reaction with our model substrate **6** using standard methylating agents such as methyl iodide or dimethyl sulfate and potassium or caesium carbonate in MeCN or DMF. Surprisingly, these efforts led exclusively to the oxidized coumarin product (Scheme 7). We therefore followed the same protocol as Connon [22,23,24,25,26,27] and Seidel [28], which involved the use of TMS-diazomethane in methanol, and successfully generated the corresponding esters in high yields. We also confirmed that the original *cis/trans* ratio in the acid substrates did not change during the esterification step. The racemic esters were subjected to the same chiral HPLC conditions as reported by Connon [22,23] with minor modifications as appropriate, yet we were not able to identify suitable methods for all types of Pinder products we generated. With the chiral HPLC methods at hand we proceeded with the testing of chiral catalysts.

### 2.3. Design, Synthesis and Evaluation of Chiral Dual Organocatalysts

Being mechanism-driven, we envisaged that the initial, reversibly formed, aldol/Knoevenagel alcohol may be subject to a kinetic acylation-ring closure event, thus precipitating the lactone product. We aimed to utilize chiral nucleophilic catalysts in order to facilitate this event and explore potential synergy in the asymmetric induction of the process.

We also planned to preserve the Lewis acid component that proved consistently essential in successful chiral catalysts for the Pinder and Castagnioli–Cushman reactions. From a practical viewpoint, we pursued stable, modular organocatalysts that could be accessed readily in one to three synthetic steps, thus supporting their wider availability and use-testing in this and mechanistically related transformations. For the Lewis acid module of our dual organocatalysts, we decided to screen squaramides, ureas, and thioureas, while for the complementary nucleophilic module, we selected the benzimidazole moiety present in the BIMAH ligands (2-(α-(alkyl)methanamine)-1H-benzimidazoles; alkyl = Me, ^i^Pr, ^i^Bu and ^t^Bu) used in Ru complexes for asymmetric hydrogenations [29].

**Scheme 8:**
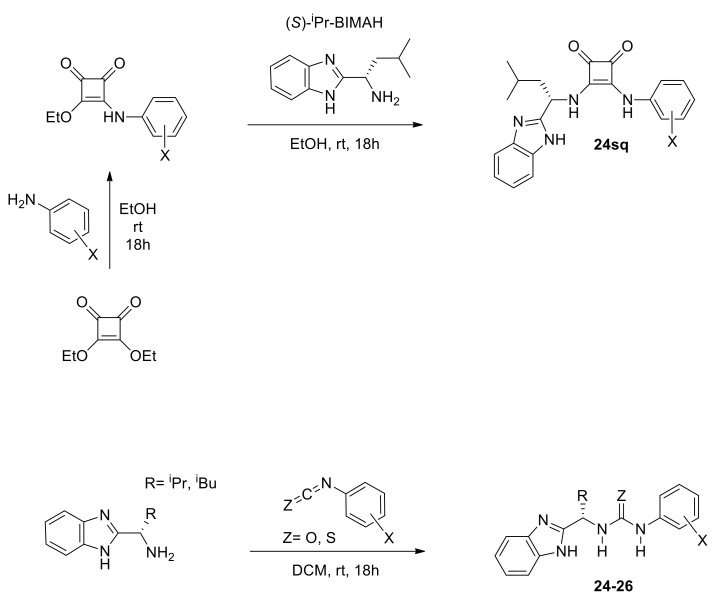
Types of chiral organocatalysts designed for the asymmetric Pinder reaction bearing a Lewis acid and a nucleophilic/basic domain.

**Scheme 9:**
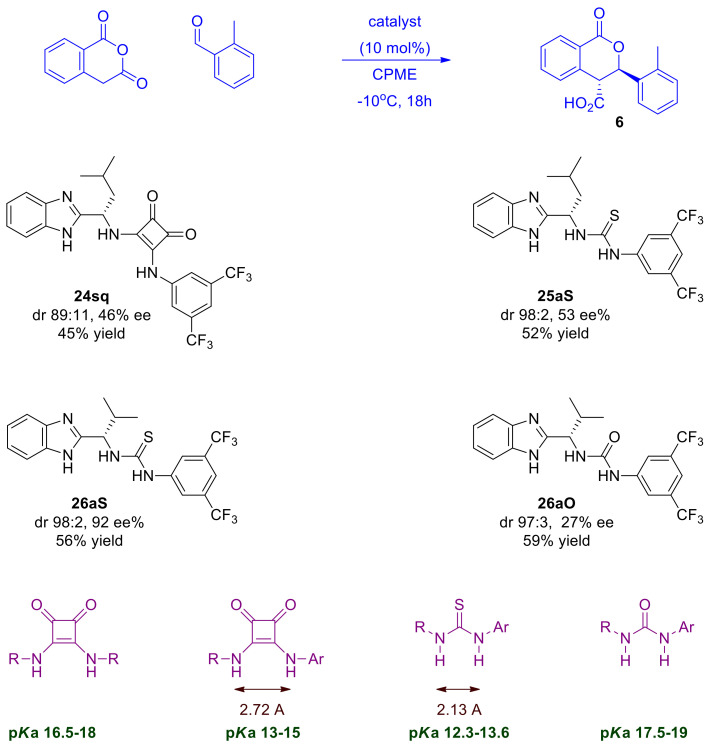
Screening of different types of dual BIMAH-based organocatalysts in the Pinder reaction.

Initially, we prepared a series of dual organocatalysts based on (*S*)-isobutyl BIMAH in order to first identify the best Lewis acid partner for the chiral benzimidazole module and optimize its sub-features as appropriate (Scheme 8). We then tested representative members of these classes of chiral organocatalysts at 10 mol% in the Pinder reaction under appropriate conditions, as identified earlier (Table 1), that suppress the uncatalyzed reaction. This approach allowed us to assess the performance of the reaction proceeding exclusively via the catalysts and revealed encouraging activity of these novel catalysts in the Pinder process (Scheme 9).

At first inspection, all catalysts tested gave improved diastereoselectivities in comparison to the uncatalyzed reaction with the *trans* isomer being formed as the major or nearly exclusive product, suggesting that these catalysts enhance the natural stereochemical preference of the Pinder reaction, at least with these typical substrates.

All types of catalysts tested gave moderate yields of the Pinder products, which prompted us to investigate further the reaction parameters in order to improve conversions, ideally at lower catalyst loadings. Prior to that, it was important to determine the optimum type of mediator and use it in all subsequent optimization work. The chiral benzimidazole-thiourea catalyst **26aS** provided the Pinder product **6** with 92 ee% and dr 98:2, thus it had superior diastereo- and enantioselectivity to all other catalyst classes. The structural and physicochemical properties of the catalysts tested suggest that the enantioselectivity of the Pinder reaction is mostly benefited by the benzimidazole catalyst bearing the Lewis acid module with the lowest p*K*a among those tested. The alkyl/aryl thiourea module (**26aS**) is the most acidic in comparison to the alkyl/alkyl squaramide (**24sq**) and alkyl/aryl urea (**26aO**) counterparts (Scheme 9) [30,31,32]. The Lewis acid’s p*K*a values alone however do not explain the significant difference in enantioselectivity observed between the thiourea catalyst **26aS** and aryl/alkyl squaramide catalyst **24sq** as these have comparable p*K*a values. The key difference between these comparably acidic moieties is the distance between the two hydrogen-bond-donor NH groups, which is much greater in squaramides than in thioureas (Scheme 9) [32]. It is therefore a combination of geometry and p*K*a of the Lewis acid component in these catalysts that governs the enantioselectivity at the transition state, whereas the diastereoselectivity, although enhanced, appears to be largely substrate controlled (see mechanistic discussion for a more detailed rationale).

**Scheme 10:**
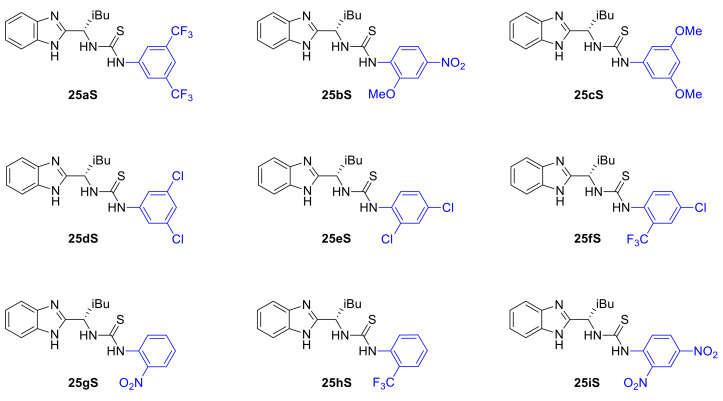
Aniline variation in the isobutyl-BIMAH/thiourea catalysts.

We decided therefore to focus further on the benzimidazole-thiourea type of catalysts particularly with respect to the effect of the aniline substituents on the stereochemical outcome of the Pinder reaction. Thus, a wide range of benzimidazole-aryl with different steric, electronic and acidic/hydrogen bond donor properties were prepared and tested in our model Pinder reaction (Scheme 10). For this purpose, we used the isobutyl thioureas rather than the isopropyl derivative of the BiMAH aminomethyl benzimidazole (Scheme 8, **26aS**) in order to capture large increments in the enantioselectivity of the process in comparison to the isobutyl prototype **25aS** (53 ee%), which may be missed when modifying the nearly excellent performing isopropyl analogue **26aS** (92 ee%). 

The results from this series of catalysts show that the reaction is particularly sensitive to the aniline domain of the organocatalyst with all modifications leading to inferior performance (Table 2). More specifically, none of the chloro-substituted analogues tested (**25dS, 25eS, 25fS**) promoted the desired reaction, a result that was shared by the most acidic dinitro analogue (**25iS**). It is evident that there is an optimum in the acidity/hydrogen bond donor capacity of the thiourea since, in addition to **25aS**, only analogues **25bS** (*o-*MeO, *p-*NO_2_) and **25hS** (*o-*CF_3_) catalyzed the Pinder reaction satisfactorily (62 and 51% yield respectively) albeit with much reduced enantioselectivity (both 18 ee%). The mono nitro derivative **25gS** led to a mixture of products being formed together with the desired lactone **6**. Interestingly, the diastereoselectivity in all cases that worked was 98:2 favoring the *trans* isomer, which is consistent with Connon’s report on *cis* selective catalysts where the steric bulk of the substituent of the terminal nitrogen in the Lewis acid part of the catalyst was found to mostly influence the diastereoselectivity [24]. Consistent with Connon’s [21] and other thiourea organocatalyzed processes, here too, the 3,5-bis(trifluoromethyl)anilino derivative is the most active and enantioselective [21,33,34,35,36]. 

### 2.4. Optimization of Reaction Conditions for the Asymmetric Pinder Reaction

Once the optimum catalyst was identified in **26aS**, we revisited the reaction conditions primarily with the aim to resolve the issue of attenuating conversion with time, leading to modest yields with all active catalysts (product yields 55–65%). In this second iteration, we examined the effect of the solvent, the concentration, the temperature and basic additives.

In terms of solvents, MeCN, DCM and toluene gave much inferior results, whereas ethereal solvents were confirmed, once more, to best serve this organocatalyzed Pinder reaction with CPME being the optimal (Table 3) [32,33,34,35,36]. Interestingly, although the diastereoselectivity remained high (98:2), the enantioselectivity dropped significantly (92 and 76 ee% at 0.16 and 0.08 M, respectively) when the dilution was doubled, suggesting the involvement of dimers, if not higher aggregates, in the enantiodetermining step. Due to solubility issues primarily with homophthalic anhydride and to a lesser extent with the catalyst, we avoided running the reaction at higher concentration in order to avoid confounding the results with complications due to mass transfer effects. The rate of the reaction also decreased in the more dilute conditions, requiring an additional day to reach the same level of conversion (*ca* 70%) when more dilute reaction conditions were utilized (36 h and 54 h at 0.16 and 0.08 M, respectively).

Another interesting effect was observed with changing the reaction temperature where an inversion temperature manifested at −10 °C. Reactions run from −20 °C to 25 °C revealed that the same enantiomer is favored across this range but not uniformly. More specifically, starting from 25 °C, the enantioselectivity increased with decreasing temperature and reached its maximum value at −10 °C, whereas a further temperature decrease resulted in gradual erosion of enantioselectivity (Table 4); the diastereoselectivity however was essentially unaffected. Non-linear effects between temperature and stereoselectivity, a phenomenon termed isoinversion [37], have been reported previously in reactions catalyzed by enzymes [38], chiral organometallic complexes [39], and organocatalysts, such as those used in the CBS reduction [40,41,42,43,44], halocyclisations [45], and in non-catalyzed processes with chiral substrates such as the Staudinger [46] and the photochemical Paterno–Buchi reaction [37].

This is a “U” or inverted “U” relationship of enantioselectivity versus temperature and should not be confused with cases where a temperature decrease or increase results in complete reversal of enantioselectivity from fully favoring one enantiomer to fully favoring the other [47]. The attenuation of enantioselectivity at higher temperatures is normally explained by the higher contribution of a racemic/uncatalyzed process or a thermodynamically-controlled process favoring the other enantiomer. Various explanations have been put forward to rationalize the attenuation of enantioselectivity below and above the inversion temperature [37,48,49] primarily pointing to entropic effects becoming more dominant than enthalpic effects. The mechanistic implications of these will be discussed later together with the effects of other parameters such as concentration and substrate type.

The next iteration of optimization was aimed at resolving the gradual attenuation of conversion rates as the reaction progressed. For example, our model reaction gave the Pinder product in 56% yield after 18 h at −10 °C and although not stalled, it required an additional 18 hours to provide a 16% increase in yield (entries 4 and 5 in Table 3).

We reasoned that because the reaction product is a carboxylic acid, it could be protonating the small amount of benzimidazole catalyst as the reaction progresses, thus lowering the effective concentration of the basic form of the catalyst and slowing down the process. This would constitute a typical case of catalysis subject to product inhibition since the protonated form of the catalyst would be incapable of acting either as a base or a nucleophile, thus negating the intended modes of action. In order to avoid formation of this catalyst sink, we decided to explore bases as additives to neutralize the acid product and preserve the unprotonated form of the catalyst.

The bases tested were carefully selected so that the p*K*aH values of their conjugate acids were sufficiently high to partially neutralize a carboxylic acid but low enough to preclude direct deprotonation of the homophthalic anhydride substrate as this event would promote the racemic/uncatalyzed process. Accordingly, 0.5 eq each of MgO (p*K*aH 9.23), 1,8-diaminonaphthalene (p*K*aH 9.31) and NaHCO_3_ (p*K*aH 10.33) were tested as additives under the optimized conditions in the absence of a catalyst in order to assess the extent of the inadvert base-induced racemic process if any. We found that after 18 h, MgO caused minimal product formation (<4%) in comparison to diaminonaphthalene (7%) and NaHCO_3_ (14%). We next interrogated the maximum stoichiometry of MgO that can be tolerated in the catalyzed reaction before the background racemic process is promoted significantly and cause erosion of enantioselectivity (Table 5).

Apparently, the low solubility of MgO combined with its weak basicity, limits the extent of the racemic process even when 4 equivalents of the inorganic additive is added. In the presence of 10 mol% catalyst however, the same amount of MgO led to significant erosion of enantioselectivity, presumably due to the partial solubilization of the base by the significant amounts of the acidic product formed by the catalyzed reaction. Limiting the amount of MgO to just 1 eq sustained the high enantioselectivity and provided significant enhancement of the reaction rate, thus corroborating our understanding of product inhibition in the catalyzed process.

### 2.5. Substrate Scope in the Asymmetric Pinder Reaction Catalyzed by BIMAH-Thioureas

**Scheme 11:**
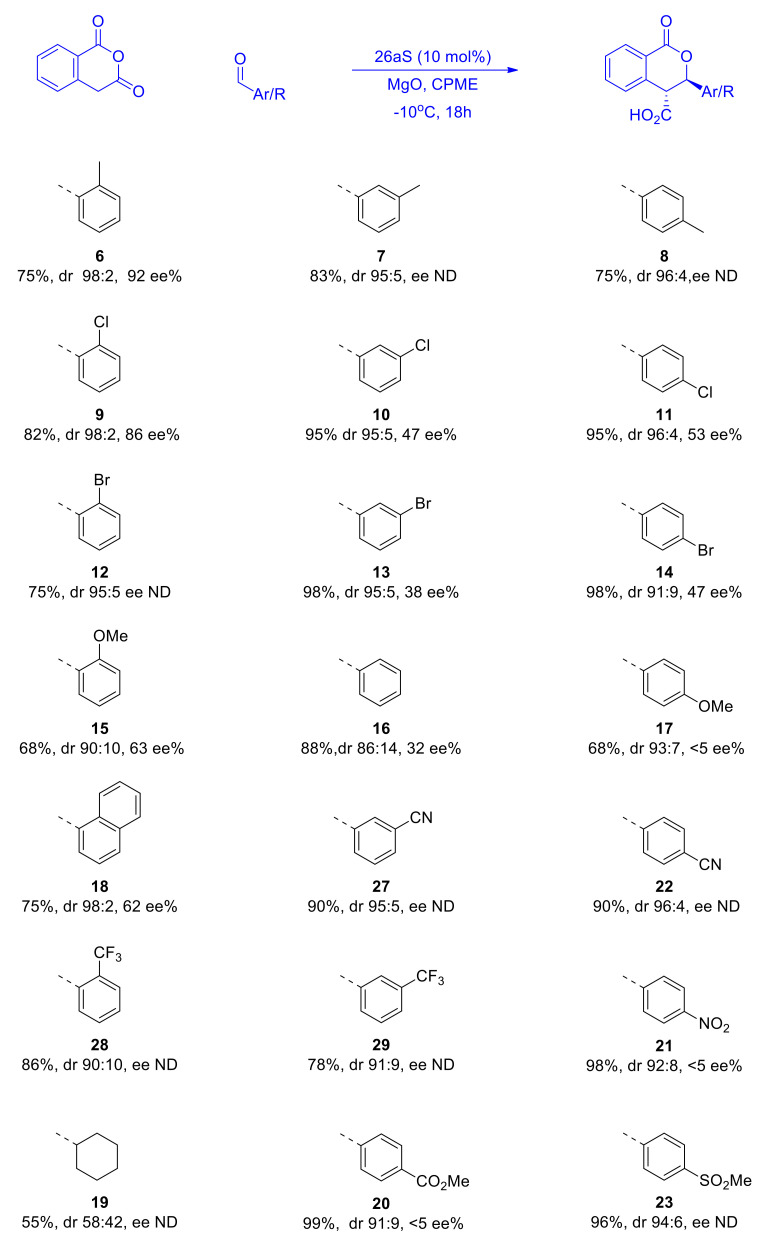
Effect of the steric and electronic properties of aldehyde substrates on the stereochemistry of the Pinder reaction catalyzed by **26aS**. The ee’s were determined either directly for the acids (**6, 8, 9**) or the Me-ester derivatives prepared with TMSCHN_2_ in MeOH as described earlier.

Having optimized the conditions for the asymmetric organocatalyzed Pinder reaction to a reasonable degree, we proceeded to examine other aldehyde substates and determine the influence of their steric and electronic properties on the stereochemical outcome (Scheme 11). The carboxylic acid products were converted to their methyl esters as described above and examined for enantiomeric excesses. As mentioned earlier, despite our best efforts, we were not able to develop chiral HPLC methods for all substrates tested; nevertheless, certain useful conclusions could be readily drawn from this dataset. 

Apparently, both steric and electronic factors in the aldehyde substrates exert significant influence on the stereochemical course of the Pinder reaction catalyzed by our BIMAH-thiourea organocatalyst. A general observation is that the *trans* isomer is invariably the dominant product and much more so than in the DIPEA promoted reaction. More than half of the substrates gave dr ≥95:5. With the exception of the product of cyclohexyl carbaldehyde, which did not improve much (58:42 vs. 53:47), the only product with less than 90% *trans* selectivity was the simple phenyl analogue, yet this constitutes a vast improvement in the trans/cis selectivity with the BIMAH-thiourea catalyst in comparison to the DIPEA process (86:14 vs. 59:41). Benzaldehydes possessing mildly electron donating or withdrawing substituents (Me, Cl, Br) at the ortho position gave the best results both in terms of enantio- and diastereoselectivity (86–92 ee% and dr 98:2). Moving the same groups in the meta and para positions caused a marginal decrease in *cis/trans* selectivity (95:5) but the enantioselectivity was more heavily attenuated (38–53 ee%). The same trend, although much more pronounced in magnitude, was observed with the strongly donating MeO group, which gave 63 ee% and dr 90:10 at the *ortho* position and <5 ee% and dr 93:7 when present at the *para* position. Similarly, strongly electron withdrawing groups at the *para* position gave products with high diastereoselectivity (91–94% *trans*), albeit negligible enantioselectivity. 

In comparison with the only other report on the catalytic asymmetric Pinder reaction, namely Connon’s protocol using catalyst **2a**, the present method with catalyst **26aS** gives similar diastereoselectivities with aromatic aldehyde substrates (90:10–98:2). Catalyst **26aS** appears to be slightly more active as it provides 75–98% yields after 18 h at −10 ℃, whereas **2a** requires 48 h at −15 ℃ to afford similar yields (for the *p-*MeO derivative, a 78% yield was obtained after 115 h). The greatest difference between **2a** and **26aS** is the asymmetric induction with **2a** giving consistently high enantioselectivities (91–99 ee%) with a variety of substrates. The much more basic amine component in **2a** may lead to relatively more stable protonated species that form stronger (ionic/H-bond) interactions with the substrates, and hence better enantiocontrol. This aspect is under consideration in our ongoing efforts to develop the next generation of organocatalysts for the Pinder and Castagnoli–Cushman reactions.

### 2.6. Mechanistic Considerations

Finally, we attempted to propose a mechanism that could reconcile the trends observed during the optimization of the reaction parameters and the response of the various substrates. Key to suggesting a plausible mechanism for this organocatalytic Pinder reaction was to provide a rationale for the following experimental facts:

1. solvents of low polarity benefit the enantioselectivity of the process
2. a mildly basic additive improves the reaction rate
3. strongly electron donating or withdrawing groups on the aldehyde substrate lead to poor enantioselectivity
4. the enantioselectivity of the catalyzed process is increased at higher catalyst loading and/or concentration
5. the enantioselectivity-temperature relationship of the catalyzed process is governed by an inversion temperature
6. the sterically congested *ortho* substituted benzaldehydes give higher enantioselectivity than their *meta* and *para* counterparts
7. the diastereoselectivity is not correlated with enantioselectivity and is enhanced in the catalyzed process.

**Scheme 12:**
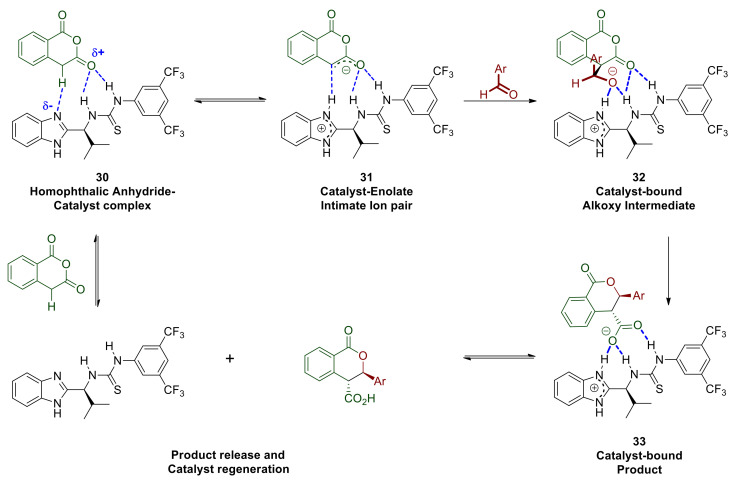
Partial mechanistic hypothesis based on solvent, additive and electrophilic substrates.

Every mechanistic hypothesis initially considers all possible proton transfers and establishment of equilibria thereof, since formation of carbon–carbon and carbon–heteroatom bonds are much more slower processes in comparison. In this context, an interaction can be envisaged between the most basic (benzimidazole-part of the catalyst) and the most acidic species in solution (homophthalic anhydride’s benzylic proton) supported further by hydrogen bonding between the thiourea-part of the catalyst and the enolisable carbonyl group of homophthalic anhydride (**30** in Scheme 12). The latter interaction renders the benzylic proton more acidic, thus its abstraction by the benzimidazole moiety is further facilitated, resulting in a hydrogen bonded intimate ion pair (**31** in Scheme 12).

**Scheme 13:**
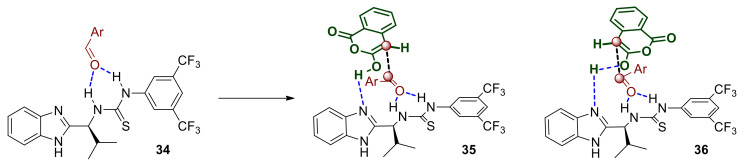
Partial mechanistic hypothesis based on solvent, additive and electron rich substrates.

The initial interaction and equilibrium that is established between the two protomeric complexes are expected to be favored in lower polarity solvents that do not disrupt the hydrogen bonding between the catalyst and the substrate or hinder it via extensively solvating either of those (rational for point **1** listed above). The enolate **31** is expected to react readily and with diminished enantioselectivity with the more electrophilic aldehydes such as those possessing strong electron withdrawing groups (rational for point **3** listed above). The formation of the corresponding initial adduct **32** where the alkoxy intermediate is bound to the catalyst does not appear to be reversible since the dr of the reaction product is maintained constant throughout the progress of the reaction, therefore cyclisation to the lactone is probably rapid. The product thus generated is still bound to the catalyst, namely as complex **33**, which is held together though strong hydrogen bonds primarily to the protonated benzimidazole and secondly to the thiourea. This aspect may work against product release and catalyst regeneration and this is probably why our approach in adding a mild base to deprotonate **33** improved the reaction rate/catalyst turnover frequency (rationale for point **2** listed above). The eventually released acid product may itself act as a proton and/or hydrogen bond donor to activate the most basic aldehydes such as *p-*methoxy-benzaldehyde, which in turn would also react quickly and with poor selectivity without engaging with the catalyst. Alternatively, aldehydes with sufficiently electron rich carbonyl groups may compete for hydrogen bonding to the thiourea Lewis acid component of the catalyst and become activated (**34** Scheme 13). The benzimidazole moiety of the catalyst may then engage via hydrogen bonding and deprotonate the anhydride thus facilitating the nucleophilic attack either via transition state **35** or **36** leading to enantiomers of the observed *trans* product.

These well-established interactions described in Scheme 12 and Scheme 13 help to provide a rational for the poor enantioselectivities observed with *para* substituted benzaldehydes with strongly electron donating or withdrawing groups (point **3**). In contrast, it is the substrates with weak electron donating/withdrawing groups and with *ortho* substituents that lack intrinsic reactivity and/or carbonyl basicity that need to be activated by other means and apparently lead to products with high ee’s. We propose that this alternative and more selective activation mode/reaction pathway is provided by an additional catalyst molecule. This notion is supported by the higher enantioselectivity observed at higher concentration/catalyst loading (point **4**), which points to the involvement of substrate-bound-catalyst dimers (if not higher aggregates). 

**Scheme 14:**
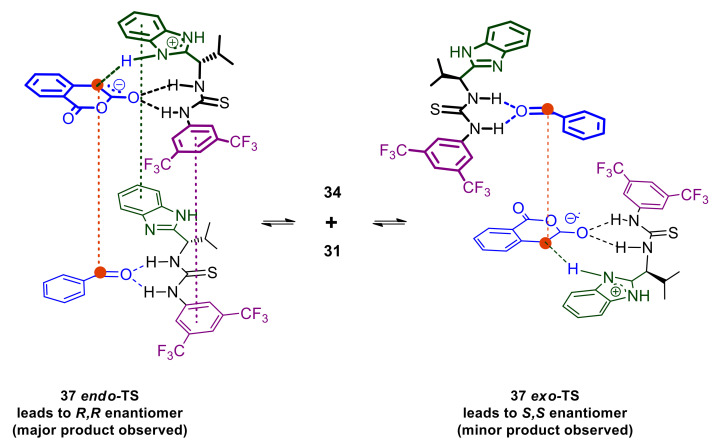
Equilibration between *cis* and *trans* aldehyde-catalyst complexes leading to competing *endo* and *exo* transition states and ultimately to opposite enantiomers.

Integrating this with the understanding already established above, a more complete mechanistic picture is assembled as depicted in Scheme 14. Using benzaldehyde as the substrate, the *trans* Pinder product is obtained with 32 ee% with the *R,R* enantiomer being formed predominately (determination of absolute configuration was based on the work reported by Connon for the same product [21]). In order to arrive at this, complex **34** should be approached by the activated catalyst-homophthalic anhydride complex **31** either according to an *endo*-like transition state or an *exo*-like transition state (**37 *endo*-TS** and **37 *exo*-TS** respectively in Scheme 14). In **37 *endo*-TS**, the alignment of the reacting centers is stabilized by several π-π stacking interactions, whereas **37 *exo*-TS** ensures minimal steric repulsions between **31** and **34**.

The **37 *endo*-TS** set up requires much higher organization, reflecting the need for lower entropy. Consequently, this is expected to be more favored as temperature decreases, thus allowing for fast formation of the key bond once **37 *endo*-TS** is assembled. In contrast, **37 *exo*-TS** is easier to start assembling as it requires only the alignment of the nucleophilic and electrophilic carbon atoms in the activated substrates but the fewer interactions holding it together also favor the disengagement of **31** and **34** from one another. **37 *exo*-TS** may accommodate more degrees of freedom, namely increases in entropy, and therefore predominates at higher temperatures. The increase in enantioselectivity favoring the *R,R* enantiomer at lower temperatures is consistent with the entropically favored **37 *endo*-TS**. Nevertheless, this rational, which predicts even greater enantioselectivity (a kinetic effect) as temperature decreases further, is valid as long as all process leading to the assembly of **37 *endo*-TS** and **37 *exo*-TS** are fast and reversible, whereas the formation of the key C-C bond forming step is the rate determining step. In other words, this appears to be consistent with the Curtin–Hammett principle in which enantiomer distribution is governed by the difference in their respective rate of formation from rapidly equilibrating **37 *endo*-TS** and **37 *exo*-TS**. Faster, irreversible formation of the *R,R* enantiomer *via* **37 *endo*-TS** would cause the equilibrium to replenish this complex via decomplexation of **37 *exo*-TS** to **31** and **34** and renewed formation of **37 *endo*-TS**. This sequence of events requires energy input for the severance/reversal of the developing carbon–carbon and any hydrogen bond interactions involved, an energy input that may not be available if the temperature is decreased beyond a critical point, namely the inversion temperature (point **5**). It is conceivable that beyond the inversion temperature the rate of interconversion between **37 *endo*-TS** and **37 *exo*-TS** becomes comparable to that of the C –C bond formation in **37 *endo*-TS**, if not the rate determining step itself. In this case the enantiomer distribution would be strongly influenced by the relative amounts of the now slowly equilibrating **37 *endo*-TS** and **37 *exo*-TS**, leading to increased enantiomer formation of the *S,S* enantiomer via **37 *exo*-TS** in comparison to the situation where fast equilibration between **37 *endo*-TS** and **37 *exo*-TS** channels the overall process reaction via the entropically favored **37 *endo*-TS** towards the *R,R* enantiomer.

**Scheme 15:**
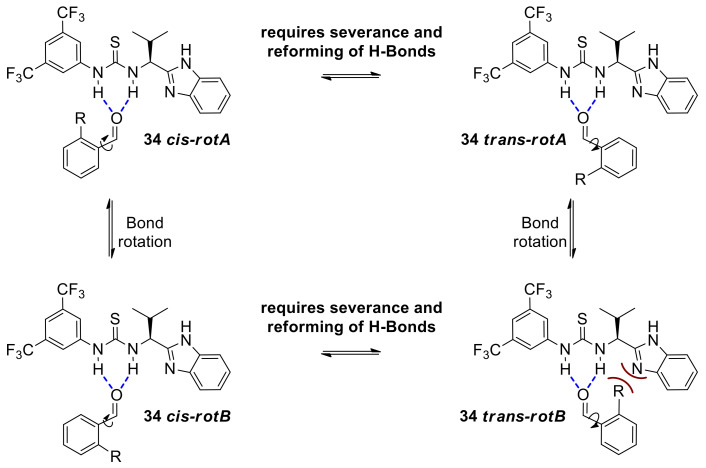
Equilibration between *cis* and *trans* aldehyde-catalyst complexes leading to competing *endo* and *exo* transition states and ultimately to opposite enantiomers.

This assertion also explains the higher enantioselectivities obtained with *ortho-*substituted benzaldehydes (point **6**). Consideration of the aryl rotamers involved in the catalyst-aldehyde complexes **34*trans*** and **34*cis***, suggests that **34*****trans-rotA*** and **34*****cis-rotB*** are the more sterically relaxed complexes so the overall position of the equilibrium favoring either the *cis* or the *trans* complex would depend on the most energetically demanding rotamers **34*****cis-rotA*** and **34*****trans-rotB*** (Scheme 15). The latter is expected to be the most destabilized rotamer due to the steric clash between the benzimidazole domain of the catalyst and an *ortho* substituent in the aldehyde substrate, whereas the corresponding steric repulsion in the former is much less severe if existing at all. Overall, this renders the set of rotamers of complex **34*trans*** less favorable and therefore complex **34*trans***, the minor component of this **34*cis-trans*** equilibrium. The resulting preference for the rotamers of **34*cis*** complex would support competitive formation of the **37 *endo*-TS** over **37 *exo*-TS** (Scheme 14) leading to higher enantioselectivities and the observed *R,R* configuration.

Such steric repulsions are absent in rotamers associated with intermediates **34*trans*** and **34*cis*** arising from *meta* and *para* substituted benzaldehydes since in these cases the aryl substituents point away from the catalyst. Even at lower temperatures where the interconversion between intermediates **34*trans*** and **34*cis*** may be significantly slower, thus determining partition into **37 *endo*-TS** and **37 *exo*-TS**, the *meta* and *para* substituent in a benzaldehyde cannot exert any steric influence on the position of the equilibrium, unlike their *ortho-* counterparts.

**Scheme 16:**
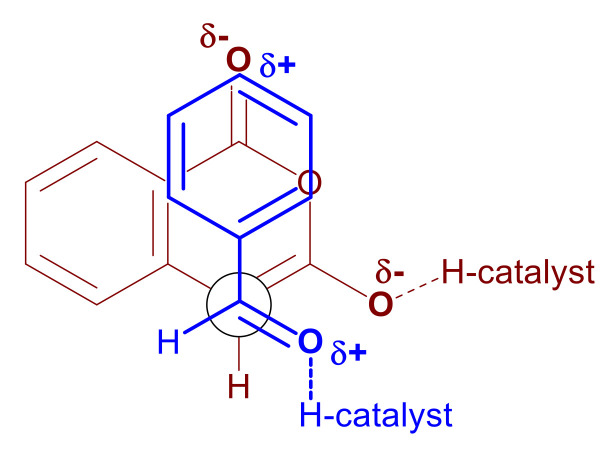
Newman projection of the transition state leading to the *trans-*substituted lactone product.

Finally, concerning point **7** at the beginning of this section, diastereoselectivity appears to be governed primarily by the dipole–dipole interactions developed between the substrates as they approach one another at the transition state. The natural polarization of an aldehyde-substituted aromatic ring is δ^+^, particularly at the *para-* position, whereas the benzoyl carbonyl in the homophthalic anhydride is δ^-^. Consequently, an opposite end alignment of these dipoles would stabilize the transition state leading to the *trans-*substituted lactone product (Scheme 14, **37 *endo*-TS** and Scheme 16). These polarization effects become more pronounced in the catalyzed reaction where the aldehyde is hydrogen bonded to the catalyst and therefore rendered additionally δ^+^ and the homophthalic anhydride is partially deprotonated with the resulting extra electron density being partially delocalized to the benzoyl oxygen. Both activating events (either by the same catalyst molecule, **31** to **33** in Scheme 12, or by two independent catalyst molecules as in **37 *endo*-TS** in Scheme 14) serve to enhance this interaction of opposite polarized substituents leading to increased diastereoselectivity in comparison to the DIPEA promoted reaction. Further evidence for this stabilizing interaction being the driving force for the high diastereoselectivity (Scheme 16) is provided by both the DIPEA-promoted and BIMAH/thiourea-catalyzed reactions, where the substitution pattern of the aryl aldehydes appears to exert little influence in the diastereoselectivity. In contrast, switching to an aliphatic aldehyde in either reaction, had a significant impact on distereoselectivity as demonstrated with cyclohexyl aldehyde as the substrate. In this case there is no electronic communication between the aldehyde group and the distant cyclohexyl carbon atom, therefore no δ^+^ polarization can be relayed at the far end of the ring. In the absence of this phenomenon, the stabilizing interaction favoring the *trans-*substituted lactone product cannot develop, hence the low diastereoselectivity with this substrate (58:42 dr).

## 3. Materials and Methods

### 3.1. Chemistry Materials and Instrumentation

All reagents and solvents were obtained from commercial sources and used without further purification unless otherwise stated. ^1^H and ^13^C NMR spectra were recorded on Bruker spectrometers at 400 or 600 MHz and 101 or 151 MHz, respectively. Chemical shifts were reported on *δ* scale in ppm with the solvent indicated as the internal reference. Coupling constants were reported in Hertz (Hz) and the standard abbreviations indicating multiplicity were used as follows: s = singlet, s(br) = broad singlet, d = doublet, t = triplet, q = quartet, and m = multiplet. High resolution mass spectrometry (HRMS) experiments were recorded with electrospray ionization (ESI) on a Synapt G2-Si mass spectrometer. The purity of all final compounds was confirmed to be ≥95% by NMR and/or HPLC using Agilent 1100 with the UV detector set at 220 nm, equipped with a Phenomenex Luna column (50 × 3.0 mm, 2.6 µm) at 40 ℃, at a flow rate of 1.0 mL/min and solvent gradient of 7 to 95% B over 5.5 min, followed by 0.5 min at 95% B, followed by gradient change to 7% B over 2 min: solvent A = 0.05% TFA in water; solvent B = 0.05% TFA in acetonitrile.

### 3.2. Experimental Procedures

#### 3.2.1. Pinder Reaction Promoted by DIPEA – General Procedure A (Racemic Products)

An oven-dried 25 mL round bottom flask containing a stirring bar charged homophthalic anhydride (100 mg, 0.62 mmol, 1eq) (and the benzaldehyde substrate if solid) and an argon atmosphere was established. Anhydrous TBME (6.0 mL) was then added via syringe followed by *o*-methyl benzaldehyde (73.5 mg, 0.62mmol, 1eq) and N,N-diisopropylethylamine (DIPEA, 80.2 mg, 0.62 mmol, 1 eq). The reaction mixture was stirred at room temperature and was monitored by HPLC until completion, typically for 18 h. The mixture was diluted with MTBE (15 mL) and washed (3 × 15 mL) with an aqueous mixture of HCl 0.5M / Brine (1:2). The organic phase was dried over MgSO_4_, filtered, and the solvent was removed in vacuo to yield the diastereomeric mixture of carboxylic acids as an off-white solid. The crude mixture of diastereomeric acids was purified by recrystallisation from DCM:Hexane (1:1) cooled to −10 °C and aged overnight. The slurry was filtered and the solid was washed with cold hexane to afford the *trans* diastereomer of **6**; 80% yield, m.p. 78–80 °C.

^1^H NMR (600 MHz, CDCl_3_) *δ* ppm 8.23 (d, *J* = 7.7 Hz, 1H), 7.65 (t, *J* = 7.5 Hz, 1H), 7.54 (t, *J* = 7.6 Hz, 1H), 7.35 (d, *J* = 7.6 Hz, 1H), 7.30–7.25 (m, 2H), 7.24–7.16 (m, 2H), 6.14 (d, *J* = 8.0 Hz, 1H), 4.49 (d, *J* = 7.9 Hz, 1H), 2.48 (s, 3H).

^13^C NMR (100 MHz, CDCl_3_): *δ* ppm 174.25, 164.1, 138.2, 136.4, 135.18, 134.4, 130.6, 129.8, 128.1, 128.6, 127.4, 127.1, 124.6, 80.21, 76.2, 49.9, 21.35.

HRMS (ESI): [MNa]^+^calculated for C_18_H_16_O_4_Na requires 282.0946; found 282.0954.

#### 3.2.2. Preparation of Methyl Esters: General Esterification Procedure Using TMS-DiazoMethane

An oven-dried vial, equipped with a small magnetic stirring bar, was charged with 0.05 mmol (1 eq) of the carboxylic acid and an inert atmosphere was established. Anhydrous THF, 0.70 mL, was transferred via syringe and 0.08 mmol (1.6 eq) of TMS diazomethane (0.04 mL of 2M solution in diethyl ether) was added to the resulting solution. The reaction mixture was diluted to a final volume of 0.9 mL by addition of 0.2 mL of anhydrous methanol. The yellow solution was stirred under inert atmosphere and monitored for completion by HPLC; typically, 30 min were required.

The reaction mixture was quenched with 0.05 mL of 0.01 M acetic acid in methanol, which caused decolorization. The reaction mixture was allowed to stir at ambient temperature for 15 min before it was concentrated to dryness in a rotary evaporator. The crude esters were recrystallized from mixtures of either ΤΒΜΕ:Hexane or Et_2_O:Hexane or DCM:Hexane. The solid products were isolated via filtration and washing with cold hexane, whereas for the oily products, the solvent system was decanted, the oil was stirred in cold hexane and the solvent was decanted again before drying under a vacuum pump.

#### 3.2.3. Organocatalyzed Pinder Reaction: General Procedure B for Screening Chiral Catalysts and the Asymmetric Pinder Reaction with Various Substrates

An oven-dried 2 mL reaction vessel equipped with a stirring bar under argon atmosphere was charged with homophthalic anhydride (20 mg, 0.123 mmol, 1 eq) and MgO (5 mg, 0.123 mmol, 1eq) and an inert atmosphere was established. Anhydrous CPME (0.25 mL) was added via syringe followed by freshly distilled *o-*methyl-benzaldehyde (14.7 mg, 123 mmol, 1 eq). The reaction mixture was then diluted with 0.5 mL CPMe, giving a final concentration of 0.16 M. It was then cooled to −10 °C with stirring. Once the desired temperature was achieved, the catalyst was added (10 mol%) and the reaction was stirred for 18 h at −10 °C. The overall conversion and diastereomeric ratio in the reaction mixture were determined by HPLC. At completion the reaction mixture was diluted with TBME (10 mL) and filtered through a fritted syringe containing Celite^TM^. The filtrate was washed with an aqueous solution of HCl (0.1 M, 3 × 15 mL) and Brine (4 mL, 2 × 15 mL) (the product is not stable to alkaline washes). The organic phase was dried over MgSO_4_ and the solvent was removed in vacuo to yield the diastereomeric mixture of carboxylic acids. The crude mixture of diastereomeric acids was purified by recrystallization as described in General Procedure A to afford the trans isomer. Conversion to the ester was achieved as described in the General Esterification Procedure. The ester derivatives were used for the determination of ee% by chiral HPLC according to the conditions described in Table 6 below:

#### 3.2.4. Synthesis of Catalyst 24sq

An oven-dried 25 mL reaction vessel containing a stirring bar under argon atmosphere was charged with 3,4-diethoxycyclobut-3-ene-1,2-dione (2 g, 12 mmol, 1 eq) and 3,5-bis(trifluoromethyl)aniline 2.8 g (12.3 mmol, 1.05 eq) followed by the addition of 8 mL EtOH (4 vol, 1.5 M final solution) and the reaction mixture was stirred for 20 h at room temperature. The resulting suspension was filtered and washed with 2 × 10 mL of chilled EtOH to give the 3-((3,5-bis(trifluoromethyl)phenyl)amino)-4-ethoxycyclobut-3-ene-1,2-dione intermediate. 

An oven-dried 25 mL reaction vessel containing a stirring bar under argon atmosphere was charged with 3-((3,5-bis(trifluoromethyl)phenyl)amino)-4-ethoxycyclobut-3-ene-1,2-dione 60 mg (0.17 mmol, 1 eq) and (*S*)-1-(1H-benzo[d]imidazol-2-yl)-2-methylpropan-1-amine 32 mg (0.17 mmol, 1 eq). The two solids were dissolved by addition of 1 mL EtOH (resulting solution 0.2 M) and the reaction mixture was stirred for 20 h at room temperature. At completion, the solvent was removed in vacuo and 1 mL of TBME was added, resulting in the formation of yellow solid. The solid was filtered and washed with 2 × 3 mL n-hexane.

#### 3.2.5. Synthesis of Catalyst 26aS: General Procedure for Thiourea/BIMAH Organocatalysts

An oven-dried 25 mL reaction vessel containing a stirring bar under argon atmosphere was charged with 1-isothiocyanato-3,5-bis(trifluoromethyl)benzene 60 mg (2.2 mmol, 1.05 eq) and (*S*)-1-(1H-benzo[d]imidazol-2-yl)-2-methylpropan-1-amine 40 mg (0.21 mmol, 1 eq). The two solids were dissolved by the addition of 0.4 mL DCM (resulting solution 0.5 M) and the reaction mixture was stirred for 20 h at room temperature. At completion, the solvent was removed in vacuo and 0.5 mL of THF was added resulting in the formation of a yellow solid. The solid was filtered and washed with 2 × 0.5 mL n-hexane.

## 4. Conclusions and Perspectives

The present work constitutes the second reported attempt towards an organocatalytic asymmetric Pinder reaction. The optimum organocatalyst from those examined consists of a thiourea Lewis acid component linked to a chiral aminomethyl-benzimidazole (BiMAH) as the Lewis base/nucleophilic component; the latter motif is hereby utilized for the first time in a urea-type organocatalyst. The catalyzed reaction is highly diastereoselective (90:10 to 98:2 dr) although reasonable enantioselectivites were obtained mainly with *ortho-* substituted benzaldehydes (62–92 ee%) rather than with their *meta-* and *para-* isomers (32–52 ee%). The asymmetric induction is subject to an isoinversion temperature effect at −10 °C and increases with catalyst loading/concentration. A mechanistic proposal that is consistent with all observations was put forward, where the enantiotopic transition states involve heterodimers between the complexes of the aldehyde-catalyst and homophthalic anhydride-catalyst. The key process to the equilibration of these appears to be the mode of aldehyde binding to the catalyst with differently oriented aldehydes in the bound state, leading to different enantiomers. Currently we are trying to exploit this aspect via novel BiMAH derivatives with 4,7-disubsituted benzimidazole cores, as the presence of substituents at these positions may drive the aldehyde to bind in a single orientation. In turn, this is expected to facilitate the assembly of one heterodimeric transition state super-complex, thus precluding enantioselectivity leakage via alternative transition states.

## Figures and Tables

**Table 1:** Screening of reaction conditions for promoting and suppressing the racemic reaction.

Entry	Promoter	Solvent (10 vol)	Temperature °C	Conversion at 18 h (a/a% of 6 by HPLC)	dr *(trans:cis)*
1	DIPEA (1 eq)	ΤΒΜΕ	−10	55	88:12
2			0	74	85:15
3			20	82	85:15
4		THF	20	94	86:14
5	NMI (2 eq)	TBME	−10	35	88:12
6			0	37	87:13
7			20	54	87:13
8	-		−10	0	-
9			0	0	-
10		CPME	−10	0	-
11			0	0	-

**Table 2:** Screening the performance of benzimidazole-thiourea catalysts in the Pinder reaction.

Entry	Catalyst	Conversion (a/a% of 6 by HPLC)	ee%	dr (*trans:cis*)
1	25aS	52	56	98:2
2	25bS	51	18	98:2
3	25cS	32	29	96:4
4	25dS	8	-	-
5	25eS	15	-	-
6	25fS	18	-	-
7	25gS	28	-	97:3
8	25hS	62	18	98:2
9	25iS	10	-	-

**Table 3:** Examining the effect of solvents and concentration using 10 mol% of catalyst 26aS at −10 °C.

Entry	Reaction Time	Solvent	Concentration	Conversion (a/a% of 6 by HPLC)	ee%	dr *(trans:cis)*
1	54 h	THF	0.16 M	80	60	98:2
2	54 h	TBME	0.16 M	58	86	98:2
3	36 h	2-MeTHF	0.16 M	21	-	-
4	18 h	CPME	0.16 M	56	92	98:2
5	36 h	CPME	0.16 M	72	92	98:2
6	54 h	CPME	0.08 M	70	76	98:2
7	54 h	CPME	0.04 M	57	69	98:2

**Table 4:** Examining the effect of temperature using catalyst 26aS at 10 mol% in CPME.

Entry	Temperature (°C)	Conversion at 18 h (a/a% of 6 by HPLC)	ee%	dr *(trans:cis)*
1	25	92	24	97:3
2	0	87	78	98:2
3	−10	56	92	98:2
4	−15	51	76	98:2
5	−20	47	66	98:2

**Table 5:** Examining the effect of MgO stoichiometry in the Pinder reaction in the presence and absence of catalyst 26aS.

Entry	MgO (eq)	Conversion (a/a% of 6 by HPLC at 36 h)		ee%
		Without Catalyst	10 mol% of 26aS	
1	none	0	56	90
2	0.5	4	65	88
3	1	6	78	89
4	4	7	82	78

**Table 6:** Chiral HPLC conditions for determining the ee%’s of Pinder products (carboxylic acids) or their ester derivatives as indicated.

Substrate	Substituent	Column Type	HPLC Settings	Retention Times
**6-acid**	*ο-*Me	Chiralcel OD-H (4.6 mm × 25 cm)	Hexane/EtOH: 80/20, 1.0 mL mL/min, 25 °C, UV detection at 220 nm.	6.1 min 6.5 min
**7-ester**	*m-*Me	Chiralcel OD-H (4.6 mm × 25 cm)	Hexane/IPA: 90/10, 1.0 mL/min, 40 °C, UV detection at 220 nm.	11.66 min 12.53 min
**8-acid**	*p-*Me	Chiralcel OD-H (4.6 mm × 25 cm)	Hexane/ EtOH: 95/5, 1.0 mL/min, 40 °C, UV detection at 220 nm.	21.33 min 22.79 min
**9-acid**	*o-*Cl	YMC Amylose SA S-5 (250 × 4.6 mml.D.)	Hexane/IPA: 80/20 1.0 mL/min, 25 °C, UV detection at 220 nm.	10.50 min 11.53 min
**10-ester**	*m-*Cl	Chiralcel IA-H (4.6 mm × 25 cm)	Hexane/IPA: 80/20, 0.6 mL/min, 25 °C, UV detection 254 nm	14.04 min 15.44 min
**11-ester**	*p-*Cl	Chiralcel IA-H (4.6 mm × 25 cm)	Hexane/IPA: 85/15, 0.6 mL/min, 25 °C, UV detection at 280 nm	21.82 min 24.24 min
**12-ester**	*o-*Br	Chiralcel OD-H (4.6 mm × 25cm)	Hexane/IPA: 75/25, 0.8 mL/min, 18 °C, UV detection at 220 nm.	7.9 min 8.5 min
**13-ester**	*m-*Br	Chiralcel IA-H (4.6 mm × 25 cm)	Hexane/IPA: 90/10, 1.0 mL min, 25^o^C, UV detection at 210 nm.	17.90 min 20.11 min
**14-ester**	*p-*Br	Chiralcel IA-H (4.6 mm × 25 cm)	Hexane/IPA: 95/5, 0.6 mL/min, 25 °C, UV detection at 230 nm.	56.38 min 63.67 min
**15-ester**	*o-*MeO	Chiralcel IA-H (4.6 mm × 25 cm)	Hexane/IPA: 85/15, 0.6 mL/min, 25 °C, UV detection at 230 nm	28.21 min 30.68 min
**16-ester**	H	Chiralcel IA-H (4.6 mm × 25 cm)	Hexane/IPA: 85/15, 0.6 mL/min, 25 °C, UV detection at 254 nm.	18.83 min 21.10 min
**17-ester**	*p-*MeO	Chiralcel IA-H (4.6 mm × 25 cm)	Hexane /IPA: 85/15, 0.6 mL/min, 25 °C, UV detection at 230 nm.	17.87 min 22.00 min
**21-ester**	*p-*NO_2_	Chiralcel IA-H (4.6 mm × 25 cm)	Hexane/IPA:80/20, 0.6 mL/min, 25 °C, UV detection at 254 nm.	13.00 min 14.77 min

## Data Availability

Not applicable.

## Supplementary Materials

The following are available online. NMR spectra and characterization of the synthesized compounds and catalysts.

## Sample Availability

Samples of the compounds are available from the authors.
