# Supplementary material for: A Novel Dual Organocatalyst for the Asymmetric Pinder Reaction and a Mechanistic Proposal Consistent with the Isoinversion Effect Thereof
Source: Molecules. 2021 Oct 22;26(21):6398. doi: 10.3390/molecules26216398 (PMC8588278; doi:10.3390/molecules26216398)
Supplement: Supplementary file 1 [file molecules-26-06398-s001.zip › molecules-1369370-supplementary.pdf]

SUPPLEMENTARY MATERIAL

A novel dual organocatalyst for the Asymmetric Pinder Reaction and a mechanistic proposal consistent with the isoinversion effect thereof

Fotini Moschona[1], Athena Vagena[1], Veroniki P. Vidali[2] and Gerasimos Rassias[1]*

[1] University of Patras, Department of Chemistry, Rio University Campus, 26504, Patra, Greece; fmoschona@windowslive.com (F.M.); athinaupatrwn@gmail.com (A.V.); rassias@upatras.gr (G.R.)

[2] NCSR "Demokritos", Institute of Nanoscience and Nanotechnology, Patr. Grigoriou and Neapoleos 27, 153 41 Athens, Greece; v.vidali@inn.demokritos.gr (V.P.V.)

Chemistry materials and instrumentation

All reagents and solvents were obtained from commercial sources and used without further purification unless otherwise stated. ^{1}H and ^{13}C NMR spectra were recorded on Bruker spectrometers at 400 or 600 MHz and 101 or 151 MHz, respectively. Chemical shifts were reported on δ scale in ppm with the solvent indicated as the internal reference. Coupling constants were reported in Hertz (Hz) and the standard abbreviations indicating multiplicity were used as follows: s = singlet, s(br) = broad singlet, d = doublet, t = triplet, q = quartet, and m = multiplet. High resolution mass spectrometry (HRMS) experiments were recorded with electrospray ionization (ESI) on Synapt G2-Si mass spectrometer. The purity of all final compounds was confirmed to be ≥95% by NMR and/or HPLC using Agilent 1100 with the UV detector set at 220 nm, equipped with a Phenomenex Luna column (50 × 3.0 mm, 2.6 μm) at 40 °C, at a flow rate of 1.0 mL/min and solvent gradient of 7 to 95% B over 5.5 min, followed by 0.5 min at 95% B, followed by gradient change to 7% B over 2 min: solvent A = 0.05% TFA in water; solvent B =0.05% TFA in acetonitrile.

Experimental Procedures

Pinder reaction promoted by DIPEA – General procedure A (racemic products)

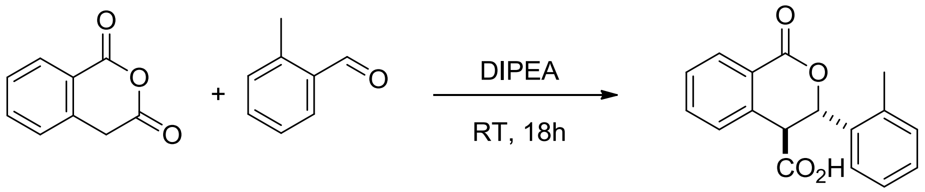

5 **31** **6**

An oven-dried 25 mL round bottom flask containing a stirring bar is charged homophthalic anhydride (100 mg, 0.62 mmol, 1eq) (and the benzaldehyde substrate if solid) and argon atmosphere was established. Anhydrous TBME (6.0 mL) was then added via syringe followed by *o*-methyl benzaldehyde (73.5 mg, 0.62mmol, 1eq) and N,N-diisopropylethylamine (DIPEA, 80.2 mg, 0.62 mmol, 1 eq). The reaction mixture was stirred at room temperature and was monitored by HPLC until completion, typically for 18h. The mixture was diluted with TBME (15 mL) and washed (3 x 15 mL) with an aqueous mixture of HCl 0.5M / Brine (1:2). The organic phase was dried over $MgSO_4$, filtered and the solvent was removed in vacuo to yield the diastereomeric mixture of carboxylic acids as an off-white solid. The crude mixture of diastereomeric acids was purified by recrystallisation from DCM:Hexane (1:1) cooled to -10 ^{o}C and aged overnight. The slurry is filtered and the solid is washed with cold hexane to afford the *trans* diastereomer of **6**; 80 % yield, m.p. 78-80 °C.

^{1}H NMR (600 MHz, $CDCl_3$) δ ppm 8.23 (d, J = 7.7 Hz, 1H), 7.65 (t, J = 7.5 Hz, 1H), 7.54 (t, J = 7.6 Hz, 1H), 7.35 (d, J = 7.6 Hz, 1H), 7.30 – 7.25(m, 2H), 7.24 – 7.16 (m, 2H), 6.14 (d, J = 8.0 Hz, 1H), 4.49 (d, J = 7.9 Hz, 1H), 2.48 (s, 3H).

^{13}C NMR (100 MHz, $CDCl_3$): δ ppm 174.25, 164.1, 138.2, 136.4, 135.18, 134.4, 130.6, 129.8, 128.1, 128.6, 127.4, 127.1, 124.6, 80.21, 76.2, 49.9, 21.35.

HRMS (ESI): $[M-H]^-$ calculated for $C_{18}H_{13}O_4$ requires 281.0819.; found 281.0834.

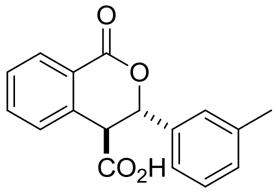

7

PreparedaccordingtogeneralprocedureA. The crude mixture of diastereomeric acids was purified by recrystallisation from TBME:Hexane (1:1) cooled to-10°C and aged overnight. The slurry is filtered and the solid is washed with cold hexaneto afford the *trans* diastereomer of **7**; 93 % yield, m.p. 118-100 °C.

^{1}H NMR (600 MHz, $CDCl_3$) δ ppm 8.18 (d, J = 7.8 Hz, 1H), 7.61 (td, J = 7.6, 1.2 Hz, 1H), 7.49 (t, J = 7.6 Hz, 1H), 7.30 (d, J = 7.7 Hz, 1H), 7.21 (dd, J = 13.7, 6.0 Hz, 2H), 7.13 (t, J = 7.3 Hz, 2H), 5.88 (d, J = 7.3 Hz, 1H), 4.36 (d, J = 7.3 Hz, 1H).

^{13}C NMR (151 MHz, $CDCl_3$) δ ppm 174.25, 163.99, 138.59, 136.42, 135.12, 134.45, 130.66, 129.88, 129.05, 128.63, 127.40, 127.11, 124.64, 123.75, 80.21, 49.87.

HRMS (ESI): $[M-H]^-$ calculated for $C_{18}H_{13}O_4$ requires 281.0819.; found 281.0829.

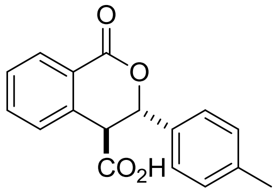

8

PreparedaccordingtogeneralprocedureA. The crude mixture of diastereomeric acids was purified by recrystallisation from DCM:Hexane (1:6) cooled to-10°C and aged overnight. The slurry is filtered and the solid is washed with cold hexaneto afford the *trans* diastereomer of **8**; 88 % yield, m.p. 88-90 °C.

^{1}H NMR (600 MHz, $CDCl_3$) δ ppm 8.19 (d, J = 7.7 Hz, 1H), 7.65 – 7.58 (m, 1H), 7.50 (t, J = 7.6 Hz, 1H), 7.30 (t, J = 8.2 Hz, 1H), 7.26 (d, J = 8.0 Hz, 2H), 7.16 (d, J = 7.9 Hz, 2H), 5.90 (d, J = 7.2 Hz, 1H), 4.36 (d, J = 7.2 Hz, 1H), 2.34 (s, 3H).

^{13}C NMR (151 MHz, $CDCl_3$) δ ppm 174.46, 164.06, 139.00, 135.17, 134.44, 133.51, 130.63, 129.47, 129.03, 127.14, 126.66, 124.66, 80.17, 49.90.

HRMS (ESI): $[M-H]^-$ calculated for $C_{18}H_{13}O_4$ requires 281.0819; found 281.0823.

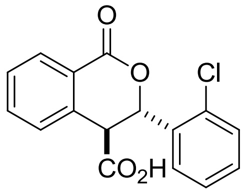

9

PreparedaccordingtogeneralprocedureA. The crude mixture of diastereomeric acids was purified by recrystallisation from TBME:Hexane (1:2) cooled to-10ºC and aged overnight. The slurry is filtered and the solid is washed with cold hexaneto afford the *trans* diastereomer of **9**; 88 % yield, m.p. 75-77 °C.

^{1}H NMR (600 MHz, $CDCl_3$) *δ*ppm 8.21 (d, *J* = 7.7 Hz, 1H), 7.62 – 7.55 (m, 1H), 7.50 (t, *J* = 7.5 Hz, 1H), 7.40 (d, *J* = 7.9 Hz, 1H), 7.30 (d, *J* = 7.6 Hz, 1H), 7.24 (d, *J* = 2.2 Hz, 1H), 7.20 (q, *J* = 7.4 Hz, 2H), 6.43 (d, *J* = 5.0 Hz, 1H), 4.44 (t, d = 5 Hz, 1H).

^{13}C NMR (151 MHz, $CDCl_3$) *δ* ppm 174.53, 163.76, 134.50, 134.23, 133.86, 132.35, 130.31, 130.26, 130.06, 129.22, 128.27, 127.83, 127.13, 124.55, 47.68.

HRMS (ESI): $[MH]^+$ calculated for $C_{17}H_{12}O_4Cl$ requires 303.0419; found 302.0422.

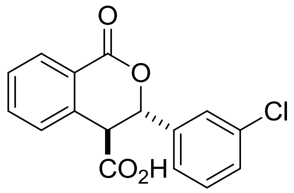

10

^{1}H NMR (600 MHz, $CDCl_3$) *δ* ppm 8.20 (d, *J* = 7.7 Hz, 1H), 7.64 (t, *J* = 7.5 Hz, 1H), 7.52 (t, *J* = 7.6 Hz, 1H), 7.41 (s, 1H), 7.35 – 7.26 (m, 4H), 5.87 (d, *J* = 7.7 Hz, 1H), 4.34 (d, *J* = 7.7 Hz, 1H).

^{13}C NMR (151 MHz, DMSO-d_6) *δ* ppm 171.33, 163.72, 140.36, 137.10, 134.96, 133.66, 130.92, 129.92, 129.09, 129.01, 127.91, 127.34, 126.07, 124.71, 79.55, 49.04.

HRMS (ESI): $[MH]^+$ calculated for $C_{17}H_{12}O_4Cl$ requires 303.0419; found 302.0411.

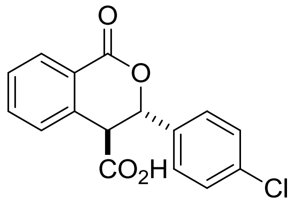

11

^{1}H NMR (600 MHz, $CDCl_3$) δ ppm 8.08 (d, J = 7.6 Hz, 1H), 7.52 (t, J = 7.5 Hz, 1H), 7.42 (t, J = 7.6 Hz, 1H), 7.25 – 7.18 (m, 6H), 5.79 (d, J = 7.5 Hz, 1H), 4.22 (d, J = 7.5 Hz, 1H).

^{13}C NMR (151 MHz, DMSO-d_6) δ ppm 171.41, 163.80, 137.10, 136.91, 134.93, 133.76, 129.91, 129.30, 129.02, 128.99, 127.93, 124.77, 79.63, 49.11.

HRMS (ESI): $[MH]^+$ calculated for $C_{17}H_{12}O_4Cl$ requires 303.0419; found 302.0425.

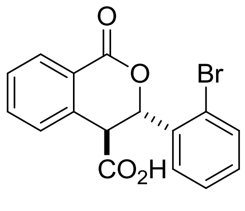

12

PreparedaccordingtogeneralprocedureA. The crude mixture of diastereomeric acids was purified by recrystallisation from DCM:Hexane (1:6) cooled to-10°C and aged overnight. The slurry is filtered and the solid is washed with cold hexaneto afford the *trans* diastereomer of **12**; 78 % yield.

^{1}H NMR (600 MHz, $CDCl_3$) δ ppm 8.23 (d, J = 7.6 Hz, 1H), 7.61 (t, J = 7.6 Hz, 2H), 7.51 (dd, J = 24.0, 16.5 Hz, 1H), 7.31 (d, J = 7.6 Hz, 1H), 7.24 (t, J = 7.4 Hz, 1H), 7.18 (dd, J = 9.7, 7.8 Hz, 2H), 6.42 (d, J = 4.3 Hz, 1H), 4.47 (d, J = 4.3 Hz, 1H).

^{13}C NMR (151 MHz, DMSO-d_6) δ ppm 171.19, 163.66, 136.61, 136.15, 135.10, 133.86, 131.37, 129.96, 129.21, 129.04, 128.54, 128.25, 124.54, 123.26, 79.47, 47.76.

HRMS (ESI) m/z: $[M-H]^-$ calculated for $C_{17}H_{10}O_4Br$ requires 344.9768; found 344.9763

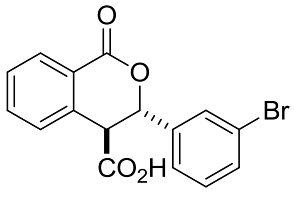

13

PreparedaccordingtogeneralprocedureA. The crude mixture of diastereomeric acids was purified by recrystallisation from TBME:Hexane (1:4) cooled to-10°C and aged overnight. The slurry is filtered and the solid is washed with cold hexaneto afford the *trans* diastereomer of **13**; 90% yield, m.p. 101-104 °C.

^{1}H NMR (600 MHz, $CDCl_3$) δ ppm 8.21 (d, *J* = 7.6 Hz, 1H), 7.65 (t, *J* = 7.4 Hz, 1H), 7.57 (s, 1H), 7.54 (t, *J* = 7.6 Hz, 1H), 7.50 (d, *J* = 7.8 Hz, 1H), 7.33 (t, *J* = 7.3 Hz, 2H), 7.25 (t, *J* = 7.8 Hz, 1H), 5.88 (d, *J* = 7.6 Hz, 1H), 4.35 (d, *J* = 7.5 Hz, 1H).

^{13}C NMR (151 MHz, DMSO-d_6) δ ppm 171.33, 163.72, 140.56, 137.10, 134.96, 132.01, 131.18, 130.23, 129.93, 129.01, 127.88, 126.46, 124.71, 122.21, 79.55, 48.94.

HRMS (ESI) m/z: $[M-H]^-$ calculated for $C_{17}H_{10}O_4Br$ requires 344.9768; found 344.9773

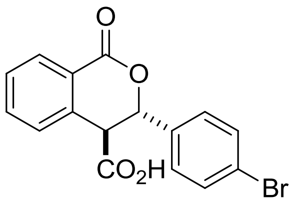

14

^{1}H NMR (600 MHz, DMSO-d_6) δ 7.98 (d, *J* = 7.6 Hz, 1H), 7.73 – 7.65 (m, 1H), 7.58 (d, *J* = 8.2 Hz, 2H), 7.50 (dd, *J* = 17.0, 9.5 Hz, 1H), 7.38 (d, *J* = 8.2 Hz, 3H), 5.98 (d, *J* = 6.7 Hz, 1H), 4.64 (d, *J* = 6.7 Hz, 1H).

HRMS (ESI) m/z: $[M-H]^-$ calculated for $C_{17}H_{10}O_4Br$ requires 344.9768; found 344.9761

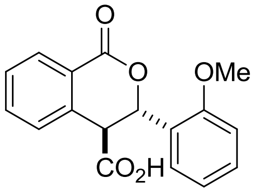

15

PreparedaccordingtogeneralprocedureA. The crude mixture of diastereomeric acids was purified by recrystallisation from Et_2O:Hexane (1:2) cooled to-10°C and aged overnight. The slurry is filtered and the solid is washed with cold hexaneto afford the *trans* diastereomer of **15**; 68% yield.

^1H NMR (600 MHz, DMSO-d_6): δ ppm8.01 (d, J = 7.7 Hz, 1H), 7.66 (dd, J = 10.8, 4.3 Hz, 1H), 7.55 – 7.49 (m, 1H), 7.37 (d, J = 7.5 Hz, 1H), 7.31 (dd, J = 11.2, 4.3 Hz, 1H), 7.05 (dd, J = 21.5, 7.9 Hz, 2H), 6.87 (t, J = 7.5 Hz, 1H), 6.22 (d, J = 5.0 Hz, 1H), 4.53 (d, J = 5.0 Hz, 1H), 3.85 (s, 3H), 2.50 (s, 4H).

HRMS (ESI) m/z: $[M-H]^-$ calculated for $C_{17}H_{13}O_5$ requires 297.0768; found 297.0771

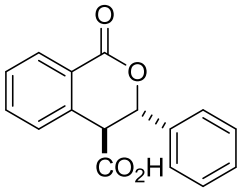

16

^1H NMR (600 MHz, CD_3CN) δ 8.09 (dd, J = 7.8, 0.9 Hz, 1H), 7.68 (td, J = 7.6, 1.3 Hz, 1H), 7.54 (d, J = 7.3 Hz, 1H), 7.41 (m, 6H), 5.98 (d, J = 6.9 Hz, 1H), 4.51 (d, J = 6.9 Hz, 1H).

^{13}C NMR (151 MHz, DMSO-d_6) δ 171.57 (s), 163.96 (s), 137.93 (s), 137.20 (s), 134.84 (s), 129.82 (s), 128.97 (t, J = 6.8 Hz), 128.06 (s), 127.26 (s), 124.89 (s), 80.32 (s), 49.15 (s).

Consistent with ref. 5 in the manuscript: Bogdanov, M. G.; Palamareva, M. D. *Tetrahedron* **2004**, *60*, 2525. doi:10.1016/j.tet.2004.01.040

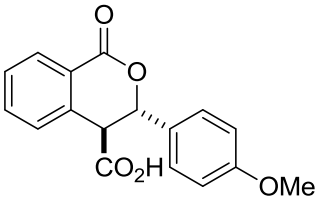

17

^1H NMR (600 MHz, DMSO-d_6) δ ppm 8.02 (t, J = 11.6 Hz, 1H), 7.66 (dd, J = 10.8, 4.3 Hz, 1H), 7.51 (dd, J = 14.9, 7.3 Hz, 1H), 7.37 (d, J = 7.5 Hz, 1H), 7.31 (dd, J = 11.2, 4.3 Hz, 1H), 7.09 – 7.00 (m, 2H), 6.87 (t, J = 7.5 Hz, 1H), 6.22 (d, J = 5.0 Hz, 1H), 4.53 (d, J = 5.0 Hz, 1H), 3.85 (s, 3H).

Consistent with ref. 5 in the manuscript: Bogdanov, M. G.; Palamareva, M. D. *Tetrahedron* **2004**, *60*, 2525. doi:10.1016/j.tet.2004.01.040

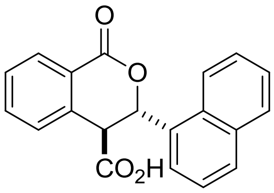

18

^{1}H NMR (600 MHz, CD_3CN) δ ppm 8.10 (dt, J = 7.6, 3.7 Hz, 1H), 7.94 (d, J = 8.6 Hz, 1H), 7.92 – 7.86 (m, 3H), 7.67 (td, J = 7.6, 1.2 Hz, 1H), 7.61 – 7.58 (m, 1H), 7.55 (ddd, J = 18.6, 10.3, 4.7 Hz, 3H), 7.41 (d, J = 7.7 Hz, 1H), 6.15 (d, J = 6.9 Hz, 1H), 4.63 (d, J = 6.9 Hz, 1H).

^{13}C NMR (151 MHz, DMSO-d_6) δ ppm 171.77 (s), 164.12 (s), 137.33 (s), 134.82 (s), 133.90 (s), 132.94 (s), 130.70 (s), 129.92 (d, J = 10.5 Hz), 129.25 (s), 128.99 (s), 128.06 (s), 127.31 (s), 126.56 (s), 125.42 (d, J = 18.2 Hz), 124.86 (s), 123.98 (s), 77.37 (s), 31.13 (s).

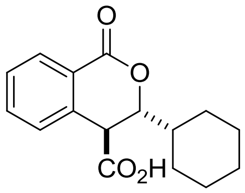

19

PreparedaccordingtogeneralprocedureA. The crude mixture of diastereomeric acids was purified by recrystallisation from TBME:Hexane (1:4) cooled to-10°C and aged overnight. The slurry is filtered and the solid is washed with cold hexaneto afford the *trans* diastereomer of **19**; 55% yield, m.p. 101-104 °C.

^{1}H NMR (600 MHz, CD_3CN) δppm 8.01 (t, J = 6.7 Hz, 1H), 7.63 (dd, J = 7.5, 6.6 Hz, 1H), 7.51 (t, J = 7.6 Hz, 1H), 7.42 (d, J = 7.5 Hz, 1H), 4.32 (dd, J = 10.0, 2.8 Hz, 1H), 4.11 (d, J = 2.7 Hz, 1H), 2.20 (d, J = 12.8 Hz, 2H), 2.02 – 1.96 (m, 1H), 1.85 – 1.75 (m, 3H), 1.69 (d, J = 12.6 Hz, 1H), 1.32 (dd, J = 12.8, 1.9 Hz, 1H), 1.26 – 1.19 (m, 1H), 1.06 (dtd, J = 23.7, 12.5, 3.3 Hz, 2H).

Consistent with ref. 5 in the manuscript: Bogdanov, M. G.; Palamareva, M. D. *Tetrahedron* **2004**, *60*, 2525. doi:10.1016/j.tet.2004.01.040

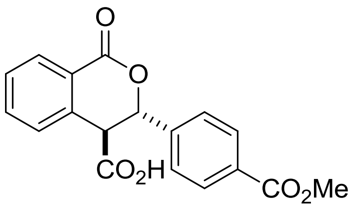

20

Preparedaccordingtogeneralprocedure A. The crude mixture of diastereomeric acids was purified by recrystallisation from EtOAc : Hexane (1:5) cooled to-10ºC and aged overnight. The slurry is filtered and the solid is washed with cold hexaneto afford the *trans* diastereomer of **20**; 66% yield, m.p. 101-104 °C.

^{1}HNMR (600 MHz, CD_3CN) δ ppm 8.06 (d, J = 7.8 Hz, 1H), 7.98 (d, J = 8.3 Hz, 2H), 7.65 (t, J = 7.5 Hz, 1H), 7.51 (d, J = 8.2 Hz, 3H), 7.37 (d, J = 7.7 Hz, 1H), 6.04 (d, J = 6.6 Hz, 1H), 4.49 (d, J = 6.5 Hz, 1H), 3.85 (s, 3H).

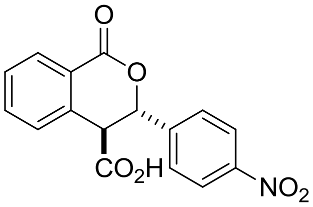

21

^{1}H NMR (600 MHz, CD_3CN) δ ppm 8.14 (d, J = 7.6 Hz, 1H), 7.83 (d, J = 7.8 Hz, 1H), 7.71 (t, J = 7.2 Hz, 1H), 7.68 – 7.56 (m, 4H), 7.41 (d, J = 7.6 Hz, 1H), 6.26 (d, J = 7.6 Hz, 1H), 4.64 (d, J = 7.6 Hz, 1H).

^{13}C NMR (151 MHz, CD_3CN) δ ppm 169.99, 163.24, 136.15, 134.96, 134.59, 132.85, 129.98, 129.79, 128.88, 128.64, 127.43, 124.51, 75.90, 48.47.

Consistent with ref. 5 in the manuscript: Bogdanov, M. G.; Palamareva, M. D. *Tetrahedron* **2004**, *60*, 2525. doi:10.1016/j.tet.2004.01.040

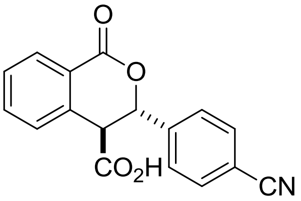

22

^{1}H NMR (600 MHz, CD_3CN) *δ* ppm 8.05 (t, *J* = 9.4 Hz, 1H), 7.72 (t, *J* = 8.6 Hz, 2H), 7.68 – 7.64 (m, 1H), 7.57 (t, *J* = 7.2 Hz, 2H), 7.51 (dd, *J* = 14.4, 6.8 Hz, 1H), 7.38 (d, *J* = 7.7 Hz, 1H), 6.04 (d, *J* = 6.6 Hz, 1H), 4.47 (d, *J* = 6.6 Hz, 1H).

^{13}C NMR (151 MHz, DMSO-*d*$_6$) *δ* ppm 171.27 (s), 163.60 (s), 143.28 (s), 136.77 (s), 134.98 (s), 133.00 (s), 132.54 (s), 129.91 (s), 129.10 (s), 128.24 (s), 128.10 (s), 124.67 (s), 111.88 (s), 79.48 (s), 48.84 (s).

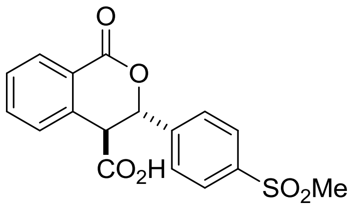

23

PreparedaccordingtogeneralprocedureA. The crude mixture of diastereomeric acids was purified by recrystallisation from Et_2O:Hexane (1:5) cooled to-10°C and aged overnight. The slurry is filtered and the solid is washed with cold hexaneto afford the *trans* diastereomer of **23**; 90% yield.

^{1}H NMR (600 MHz, DMSO-*d*$_6$) *δ* ppm 7.98 (t, *J* = 9.7 Hz, 1H), 7.94 (d, *J* = 8.2 Hz, 2H), 7.75 – 7.64 (m, 3H), 7.57 – 7.47 (m, 1H), 7.40 (d, *J* = 7.6 Hz, 1H), 6.15 (d, *J* = 6.2 Hz, 1H), 4.72 (d, *J* = 6.2 Hz, 1H), 3.21 (s, 3H).

HRMS (ESI) m/z: $[M-H]^-$calculated for $C_{17}H_{13}O_6S$ requires 345.0438; found 345.0434

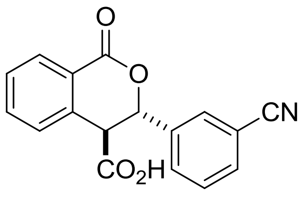

27

^{1}H NMR (600 MHz, CD_3CN) *δ* ppm 8.09 (d, *J* = 7.4 Hz, 1H), 7.80 (s, 1H), 7.77 – 7.66 (m, 3H), 7.62 – 7.49 (m, 2H), 7.42 (d, *J* = 7.4 Hz, 1H), 6.02 (d, *J* = 6.6 Hz, 1H), 4.51 (d, *J* = 6.6 Hz, 1H).

^{13}C NMR (151 MHz, DMSO-*d*$_6$) δ 171.27, 163.62, 139.49, 137.09, 135.07, 132.95, 132.34, 131.24, 130.37, 130.03, 129.10, 127.82, 124.64, 118.85, 112.05, 79.33, 48.94.

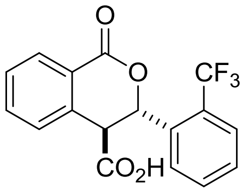

28

^{1}H NMR (600 MHz, CD_3CN) δ ppm 8.16 (d, J = 7.8 Hz, 1H), 7.84 (d, J = 7.8 Hz, 1H), 7.73 (td, J = 7.6, 1.1 Hz, 1H), 7.70 – 7.57 (m, 4H), 7.43 (t, J = 8.0 Hz, 1H), 6.28 (d, J = 7.7 Hz, 1H), 4.66 (d, J = 7.6 Hz, 1H).

^{13}C NMR (151 MHz, CD_3CN) δ ppm 169.86 (s), 163.18 (s), 136.00 (s), 134.95 (s), 134.58 (s), 132.83 (s), 129.97 (s), 129.78 (s), 128.90 (s), 128.62 (s), 127.41 (s), 126.65 (s), 124.48 (d, J = 4.7 Hz), 75.83 (s).

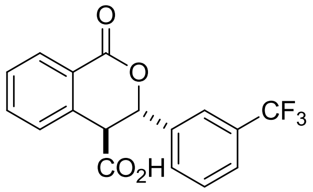

29

^{1}H NMR (600 MHz, $CDCl_3$) δ ppm 8.19 (d, J = 7.6 Hz, 1H), 7.68 – 7.57 (m, 4H), 7.55 – 7.47 (m, 2H), 7.32 (d, J = 7.6 Hz, 1H), 5.95 (d, J = 7.7 Hz, 1H), 4.35 (d, J = 7.7 Hz, 1H).

Preparation of methyl esters - General Esterification Procedureusing TMS-diazomethane

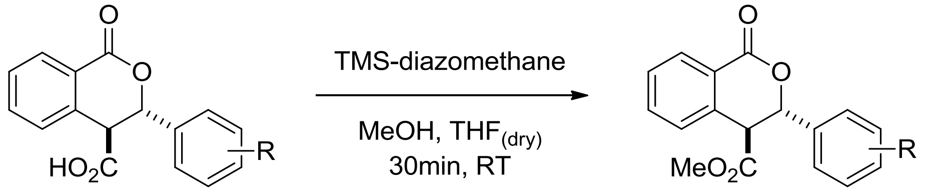

Anoven-dried vial, equipped with a small magnetic stirring bar, is charged with 0.05 mmol(1eq) of the carboxylic acid and inert atmosphere is established. Anhydrous THF, 0.70 ml, is transferred via syringe and to the resulting solution is added 0.08 mmol, (1.6 eq) of TMSdiazomethane (0.04 mlof 2M solution in diethyl ether). Thereactionmixtureisdilutedto a final volume of 0.9mlby addition of 0.2mlof anhydrous methanol. Theyellowsolutionisstirred under inert atmosphere and monitored for completion by HPLC; typically, 30 minutes are required.

Tothereactionmixtureisquenched with 0.05mlof 0.01 Msolutionofaceticacidinmethanol which causes decolorization. The reaction mixture is allowed to stir at ambient temperature

for 15 minutes before it is concentrated to dryness in a rotary evaporator. The crude esters were recrystallized from mixtures of either TBME: Hexane orEt_2O: Hexane or DCM:Hexane. The solid products were isolated via filtration and washing with cold hexane whereas for the oilyproducts, the solvent system was decanted, the oil was stirred in cold hexane and solvent decanted again before drying under a vacuum pump.

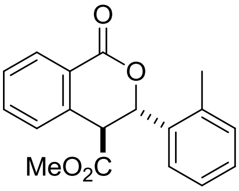

38

Prepared according to the general esterification procedure. The crude mixture of diastereomeric esters was purified by recrystallisation from DCM:hexane (1:8), cooled to-10°C and aged overnight. The slurry is filtered and the solid is washed with cold hexaneto afford the *trans* ester **38**; 66% yield, m.p. 114-116 °C.

^{1}H NMR (400 MHz, $CDCl_3$) δ ppm 8.20 (d, 1H, J = 7.7 Hz), 7.62 (app. t, 1H), 7.51 (app. t, 1H), 7.31 (d,1H, J = 7.3 Hz) 7.28-7.13 (m, 4H), 6.08 (d, 1H, J = 8.7 Hz), 4.48 (d, 1H, J = 8.7 Hz), 3.68 (s, 3H),2.45 (s, 3H).

^{13}CNMR (100 MHz, $CDCl_3$): δ ppm 169.7, 163.8, 136.1 (2xCq), 133.9, 133.7, 130.6,130.3, 128.7, 128.4, 126.4, 126.2, 125.9, 124.1, 77.1, 52.2, 48.7, 18.9

HRMS (ESI)m/z: $[MNa]^+$calculated for $C_{18}H_{16}O_4Na$ requires 319.0946; found 319.0953.

Consistent with ref. 10 in the manuscript: Cornaggia, C.; Manoni, F.; Torrente, E.; Tallon, S.; Connon, S. J. *Org.Lett.* **2012**, *14*(7), 1850-1853. doi:10.1021/ol300453s

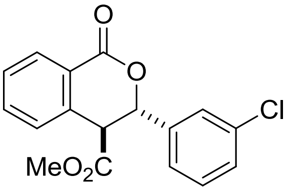

39

Prepared according to the general esterification procedure. The crude mixture of diastereomeric esters was purified by recrystallisation from Et_2O : Hexane (1:6), cooled to-10°C and aged overnight. The slurry is filtered,the solid isdrained of solvent and dried under vacuum (no hexane wash) to afford the *trans* ester **39**; 79% yield, m.p. 70-72 °C.

^{1}HNMR (400 MHz, $CDCl_3$) δ ppm 8.18 (d, J = 7.5 Hz, 1H), 7.62 (app. t, 1H), 7.50 (app. t, 1H), 7.41 (s,1H), 7.37-7.24 (m, 3H), 7.21 (d, 1H, J = 7.5 Hz), 5.82 (d, 1H, J = 8.5 Hz), 4.30 (d, 1H, J = 8.5 Hz), 3.72 (s, 3H)

^{13}CNMR (100 MHz, $CDCl_3$) δ ppm 170.0, 163.8, 138.7, 135.9, 134.9, 134.7, 130.9, 130.2, 129.5, 129.1, 127.1, 126.8, 125.1, 124.4, 79.9, 52.9, 50.8

HRMS (ESI) m/z: $[MH]^+$ calculated for $C_{17}H_{14}O_4Cl$ requires 317.0581; found 317.0592

Consistent with ref. 10 in the manuscript: Cornaggia, C.; Manoni, F.; Torrente, E.; Tallon, S.; Connon, S. J. *Org.Lett.* **2012**, *14*(7), 1850-1853. doi: 10.1021/ol300453s

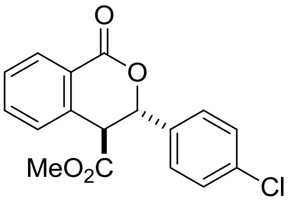

40

Prepared according to the general esterification procedure. Recrystallisation was attempted from TBME: Hexane (1:5), DCM: Hexane (1:7), Et_2O:Hexane (1:4); in all cases the *trans* ester **40** was isolated as oil which was washed with hexane;71% yield.

^{1}HNMR (400 MHz, $CDCl_3$): δppm8.18 (d, 1H, J = 7.7 Hz), 7.61 (app. t, 1H), 7.50 (app. t, 1H), 7.39-7.29 (m, 4H), 7.19 (d, 1H, J = 7.7 Hz), 5.82 (d, 1H, J = 8.7 Hz), 4.30 (d, 1H, J = 8.7 Hz), 3.71 (s,3H)

13CNMR (100 MHz, $CDCl_3$): δppm170.0, 163.9, 136.1, 135.3, 135.2, 134.7, 130.9, 129.12, 129.11, 128.4, 126.7, 124.5, 80.1, 52.9, 50.8

HRMS (ESI)m/z: $[MH]^+$calculated for $C_{17}H_{14}O_4Cl$ requires 317.0581; found 317.0577.

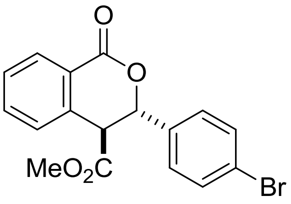

41

Prepared according to the general esterification procedure. The crude mixture of diastereomeric esters was purified by recrystallisation from Et_2O : Hexane (1:2), cooled to-10°C and aged overnight. The slurry is filtered, and the yellow solid is washed with cold mixture Et_2O : Hexane (1:2), to afford the *trans* ester **41**; 84% yield, m.p. 138-140 °C.

^{1}HNMR (400 MHz, $CDCl_3$) δ ppm 8.18 (d, 1H, J = 7.8 Hz), 7.65-7.57 (app. t, 1H), 7.55-7.45 (m, 3H),7.28 (d, 2H, J = 7.2 Hz), 7.19 (d, 1H, J = 7.5 Hz), 5.81 (d, 1H, J = 8.4 Hz), 4.29 (d, 1H, J = 8.4 Hz), 3.71 (s, 3H)

^{13}CNMR (100 MHz, $CDCl_3$) δ ppm 169.1, 163.8, 135.9, 135.7, 134.6, 132.0, 130.8, 129.1, 128.6, 126.7, 124.4, 123.3, 80.0, 52.9, 50.7

HRMS (ESI) m/z: $[MH]^+$calculated for $C_{17}H_{14}O_4Br$ requires 361.0070; found 361.0066

Consistent with ref. 10 in the manuscript: Cornaggia, C.; Manoni, F.; Torrente, E.; Tallon, S.; Connon, S. J. *Org.Lett.* **2012**, *14*(7), 1850-1853. doi: 10.1021/ol300453s

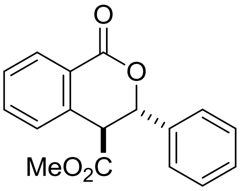

42

Preparedaccordingtogeneralesterification procedure. The crude mixture of diastereomeric esters was purified by recrystallisation from TBME: Hexane (1:4), cooled to-10°C and aged overnight. The slurry is filtered, and the yellow solid is washed with cold hexaneto afford the *trans* ester **42**; 58% yield, m.p. 118-120 °C.

^{1}H NMR (400 MHz, $CDCl_3$) δ ppm 8.19 (d, 1H, J = 8.0 Hz), 7.60 (app. t, 1H), 7.49 (app. t, 1H), 7.44-7.30 (m, 5H), 7.20 (d, 1H, J = 7.7 Hz), 5.86 (d, 1H, J = 8.3 Hz), 4.35 (d, 1H, J =8.3 Hz), 3.69 (s, 3H)

^{13}CNMR (101 MHz, $CDCl_3$) δ ppm 170.3, 164.1, 136.8, 136.3, 134.5, 130.8, 129.2, 129.0, 128.9, 126.92, 126.90, 124.8, 80.8, 52.8, 50.9

HRMS (ESI) m/z: $[MNa]^+$ calculated for $C_{17}H_{14}O_4Na$ requires 305.0790; found 305.0805.

Consistent with ref. 10 in the manuscript: Cornaggia, C.; Manoni, F.; Torrente, E.; Tallon, S.; Connon, S. J. *Org.Lett.* **2012**, *14*(7), 1850-1853. doi: 10.1021/ol300453s

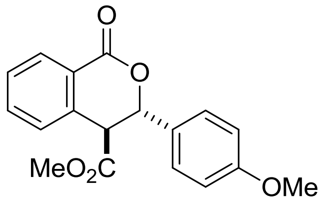

43

Prepared according to the general esterification procedure. No further purification was necessary; 64% yield, m.p. 82-84 °C.

^{1}HNMR (400 MHz, $CDCl_3$): δ ppm 8.18 (d, 1H, J = 7.8 Hz), 7.60 (app. t, 1H), 7.49 (app. t, 1H), 7.31 (d, 2H, J = 8.6 Hz), 7.19 (d, 1H, J = 7.8 Hz), 6.88 (d, 1H, J = 8.6 Hz), 5.77 (d, 1H, J = 9.0 Hz), 4.34 (d, 1H, J = 9.0 Hz), 3.80 (s, 3H), 3.69 (s, 3H);

^{13}CNMR (101MHz, $CDCl_3$): δ ppm 170.3, 164.4, 160.2, 136.6, 134.5, 130.8, 128.9, 128.7, 128.5, 126.7, 124.7, 114.2, 80.7, 55.4, 52.8, 50.9

HRMS (ESI)m/z: $[MNa]^+$ calculated for $C_{18}H_{16}O_5Na$ requires 335.0895; found 335.0905.

Consistent with ref. 10 in the manuscript: Cornaggia, C.; Manoni, F.; Torrente, E.; Tallon, S.; Connon, S. J. *Org.Lett.* **2012**, *14*(7), 1850-1853. doi: 10.1021/ol300453s

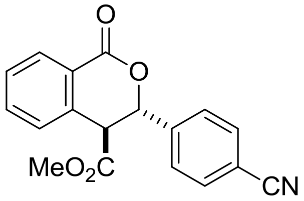

44

Prepared according to the general esterification procedure. The crude mixture of diastereomeric esters was purified by recrystallisation from Et_2O: Hexane (1:4), cooled to-10°C and aged overnight. The slurry is filtered, and the yellow solid is washed with cold mixture Et_2O : Hexane (1:2), to afford the *trans* ester **44**; 54% yield.

^{1}HNMR (400 MHz, $CDCl_3$) δ ppm 8.21 (d, J = 7.9 Hz, 1H), 7.70 (d, J = 8.5 Hz, 2H), 7.65 (app. t, J = 7.9 Hz, 1H), 7.58.7.51 (m, 3H), 7.22 (d, J = 7.7 Hz, 1H), 5.94 (d, J = 8.5 Hz, 1H), 4.32 (d, J = 8.5 Hz, 1H), 3.76 (s, 3H)

^{13}CNMR (101MHz, $CDCl_3$) δ ppm 169.6, 163.4, 141.7, 135.4, 134.7, 132.6, 130.8, 129.2, 127.6, 126.7, 124.2, 118.2, 113.2, 78.6, 52.9, 50.5

HRMS (ESI) m/z: $[M+H]^+$calculated for $C_{18}H_{14}NO_4$ requires 308.0917; found: 308.0926

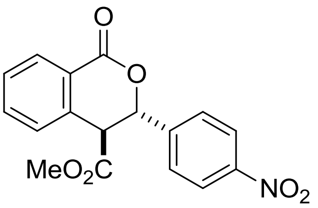

45

Preparedaccordingtogeneralesterification procedure. The crude mixture of diastereomeric esters was purified by recrystallisation from DCM: Hexane (1:8), cooled to-10°C and aged overnight. The slurry is filtered, and the yellow solid is washed with cold hexaneto afford the *trans* ester **45**; 48% yield, m.p. 131-133 °C.

^{1}H NMR (400 MHz, $CDCl_3$) δ ppm 8.24 (d, 2H, J = 8.6 Hz), δ 8.19 (d, 1H, J = 7.8 Hz), 7.68-7.57 (m, 3H), 7.52 (app. t, 1H), 7.21 (d, 1H, J = 7.8 Hz), 5.97 (d, 1H, J = 8.3 Hz), 4.32 (d, 1H, J = 8.3 Hz),3.73 (s, 3H)

^{13}C NMR (101MHz, $CDCl_3$) δ ppm169.7, 163.4, 148.4, 143.7, 135.5, 134.9, 131.0, 129.4, 127.9, 126.8, 124.3, 124.1, 79.5, 53.1, 50.7

HRMS (ESI): m/z: $[M-H]^-$ calculated for $C_{17}H_{12}NO_6$ requires 326.0670; found: 326.0674.

Consistent with ref. 10 in the manuscript: Cornaggia, C.; Manoni, F.; Torrente, E.; Tallon, S.; Connon, S. J. *Org.Lett.* **2012**, *14*(7), 1850-1853. doi: 10.1021/ol300453s

Organocatalysed Pinder reaction – General procedure B for screening chiral catalysts and the asymmetric Pinder reaction with various substrates

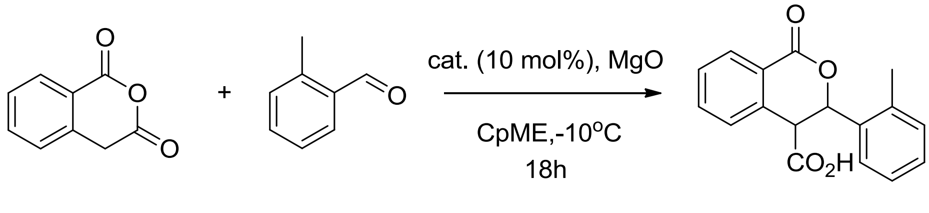

An oven-dried 2 mL reaction vessel equipped with a stirring bar under argon atmosphere was charged with homophthalic anhydride (20 mg, 0.123 mmol, 1 eq), MgO (5 mg, 0.123 mmol, 1eq) and inert atmosphere was established. Anhydrous CpMe (0.25 mL) was added via syringe followed by freshly distilled *o*-methyl-benzaldehyde (14.7 mg, 123 mmol, 1eq). The reaction mixture was then diluted with 0.5 mL CpMe, giving a final concentration of 0.16M, then cooled to -10 °C with stirring. Once the desired temperature was achieved the catalyst is added (10 mol%) and the reaction was stirred for 18h at -10 °C. The overall conversion and diastereomeric ratio in the reaction mixture were determined by HPLC. At completion the reaction mixture was diluted with TBME (10 mL) and filtered through a fritted syringe containing Celite^{TM}. The filtrate was washed with an aqueous solution of HCl (0.1M, 3 x 15 mL) and Brine (4 ml, 2 x 15 mL). (The product is not stable to alkaline washes). The organic phase was dried over MgSO4 and the solvent was removed in vacuo to yield the diastereomeric mixture of carboxylic acids. The crude mixture of diastereomeric acids was purified by recrystallisation as described in General Procedure A to afford the trans isomer. Conversion to the ester was achieved as described in the General Esterification Procedure. The ester derivatives were used for the determination of ee% by chiral HPLC according to the conditions described in the Table below:

**Substrate**	**Substituent**	**Column type**	**HPLC settings**	**Retention Times**
**6-acid**	*o*-Me	Chiralcel OD-H (4.6 mm x 25 cm)	Hexane/EtOH: 80/20, 1.0 mL mL/min, 25°C, UV detection at 220 nm.	6.1 min 6.5 min
**7-ester**	*m*-Me	Chiralcel OD-H (4.6 mm x 25 cm)	Hexane/IPA: 90/10, 1.0 mL/min, 40°C, UV detection at 220 nm.	11.66 min 12.53 min
**8-acid**	*p*-Me	Chiralcel OD-H (4.6 mm x 25 cm)	Hexane/ EtOH: 95/5, 1.0 mL/min, 40°C, UV detection at 220 nm.	21.33 min 22.79 min
**9-acid**	*o*-Cl	YMC Amylose SA S-5 (250 x 4.6 mml.D.)	Hexane/IPA: 80/20 1.0 mL/min, 25°C, UV detection at 220 nm.	10.50 min 11.53 min
**10-ester**	*m*-Cl	Chiralcel IA-H (4.6 mm x 25 cm)	Hexane/IPA: 80/20, 0.6 mL/min, 25°C, UV detection 254 nm	14.04 min 15.44 min
**11-ester**	*p*-Cl	Chiralcel IA-H (4.6 mm x 25 cm)	Hexane/IPA: 85/15, 0.6 mL/min, 25°C, UV detection at 280 nm	21.82 min 24.24 min

**12-ester**	*o*-Br	Chiralcel OD-H (4.6 mm x 25cm)	Hexane/IPA: 75/25, 0.8 mL/min,18°C, UV detection at 220 nm.	7.9 min 8.5 min
**13-ester**	*m*-Br	Chiralcel IA-H (4.6 mm x 25 cm)	Hexane/IPA: 90/10, 1.0 mL min, 25°C, UV detection at 210 nm.	17.90 min 20.11 min
35	*p*-Br	Chiralcel IA-H (4.6 mm x 25 cm)	Hexane/IPA: 95/5, 0.6 mL/min, 25°C, UV detection at 230 nm.	56.38 min 63.67 min
36	*o*-MeO	Chiralcel IA-H (4.6 mm x 25 cm)	Hexane/IPA : 85/15, 0.6 mL/min,25 ^{o}C, UV detection at 230 nm	28.21 min 30.68 min
37*	H	Chiralcel IA-H (4.6 mm x 25 cm)	Hexane/IPA: 85/15, 0.6 mL/min, 25°C, UV detection at 254 nm.	18.83 min 21.10 min
38	*p*-MeO	Chiralcel IA-H (4.6 mm x 25 cm)	Hexane/IPA: 85/15, 0.6 mL/min, 25°C, UV detection at 230 nm.	17.87 min 22.00 min
44	*p*-NO_2	Chiralcel IA-H (4.6 mm x 25 cm)	Hexane/IPA:80/20, 0.6 mL/min, 25°C, UV detection at 254 nm.	13.00 min 14.77 min

* This product was also assessed by modification of the chiral HPLC method reported in ref. 10 of the manuscript: Cornaggia, C.; Manoni, F.; Torrente, E.; Tallon, S.; Connon, S. J. *Org.Lett.* **2012**, *14*(7), 1850-1853. doi: 10.1021/ol300453s.Using OD-H, Hex:IPA 90:10 and flow rate 1.00 ml/minwe found that the enantiomer eluting second was formed in excess (32 ee%, consistent across both OD-H and IA-H methods) which corresponds to the *R*,*R* enantiomer according to the reference above.This is depicted below:

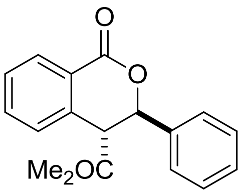

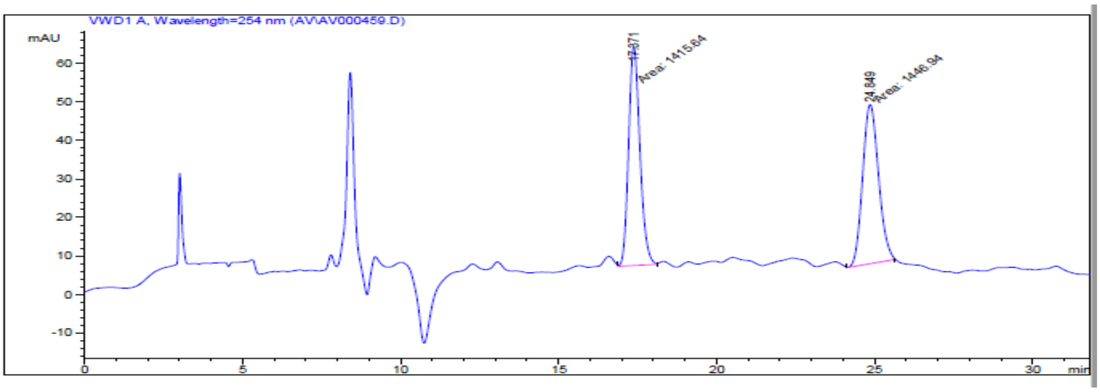

Signal 1: VWD1 A, Wavelength=254 nm

Peak #	RetTime [min]	Type	Width [min]	Area mAU *s	Height [mAU]	Area %
1	17.371	MM	0.4149	1415.63513	56.86457	49.4532
2	24.849	MM	0.5830	1446.93738	41.36269	50.5468

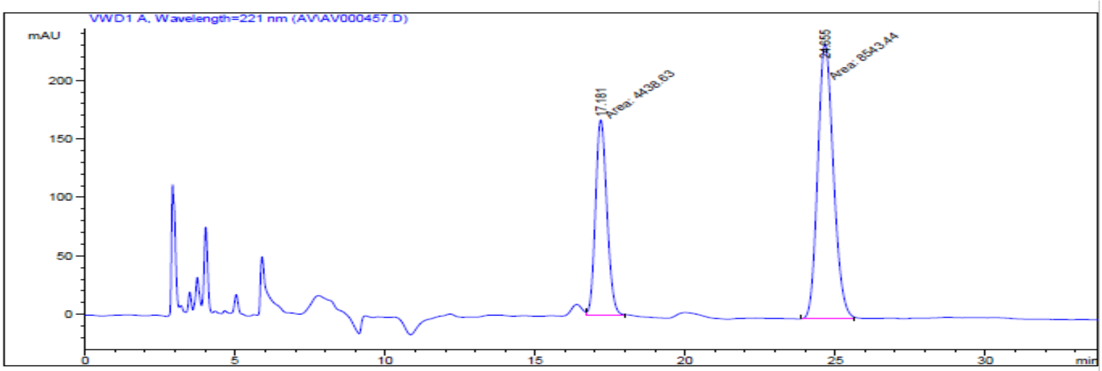

Signal 1: VWD1 A, Wavelength=221 nm

Peak #	RetTime [min]	Type	Width [min]	Area mAU *s	Height [mAU]	Area %
1	17.181	MM	0.4422	4438.62744	167.30299	34.1904
2	24.655	MM	0.6063	8543.44238	234.85391	65.8096

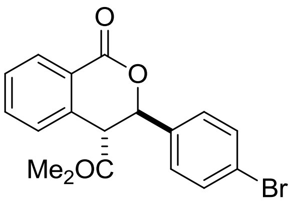

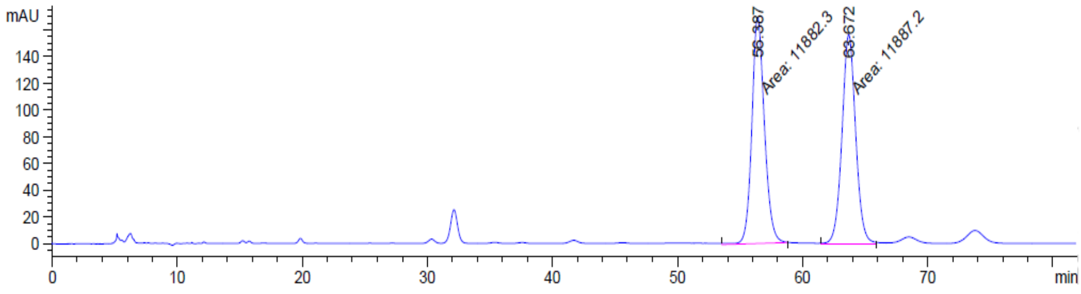

Peak #	RetTime [min]	Type	Width [min]	Area [mAU*s]	Height [mAU]	Area %
1	56.387	MM	1.1680	1.18823e4	169.55489	49.9897
2	63.672	MM	1.2651	1.18872e4	156.60956	50.0103

Totals : 2.37695e4 326.16444

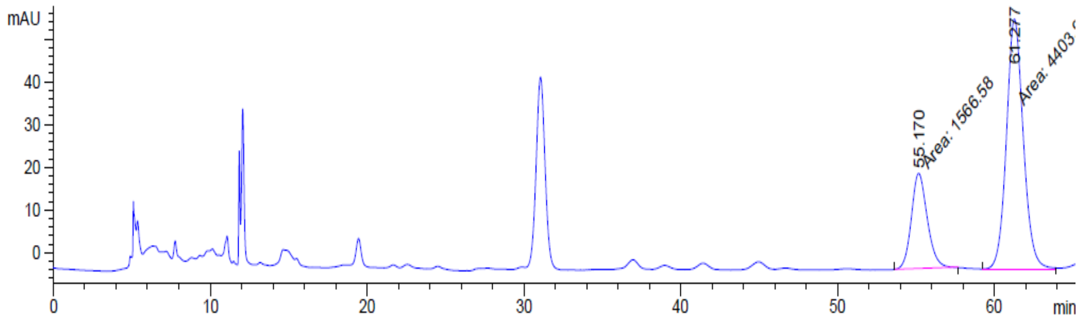

Peak #	RetTime [min]	Type	Width [min]	Area [mAU*s]	Height [mAU]	Area %
1	55.170	MM	1.1717	1566.58459	22.28381	26.2386
2	61.277	MM	1.2530	4403.93945	58.57781	73.7614

Totals : 5970.52405 80.86162

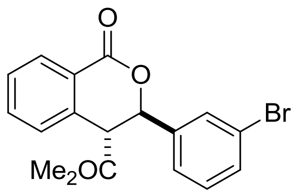

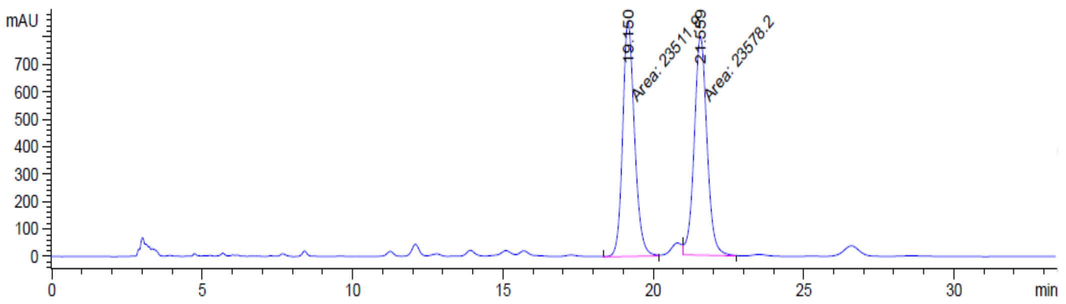

Peak #	RetTime [min]	Type	Width [min]	Area [mAU*s]	Height [mAU]	Area %
1	19.150	MM	0.4576	2.35119e4	856.42297	49.9295
2	21.559	MM	0.4927	2.35782e4	797.57581	50.0705

Totals : 4.70901e4 1653.99878

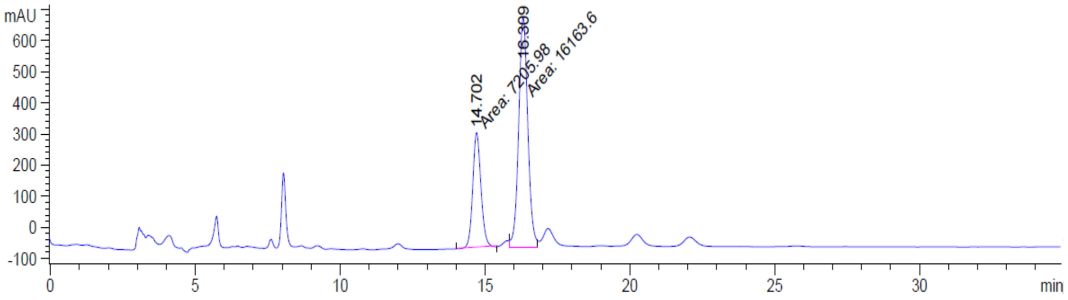

Peak #	RetTime [min]	Type	Width [min]	Area [mAU*s]	Height [mAU]	Area %
1	14.702	MM	0.3262	7205.97559	368.14929	30.8349
2	16.309	MM	0.3651	1.61636e4	737.94202	69.1651

Totals : 2.33695e4 1106.09131

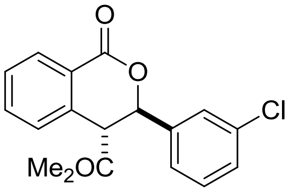

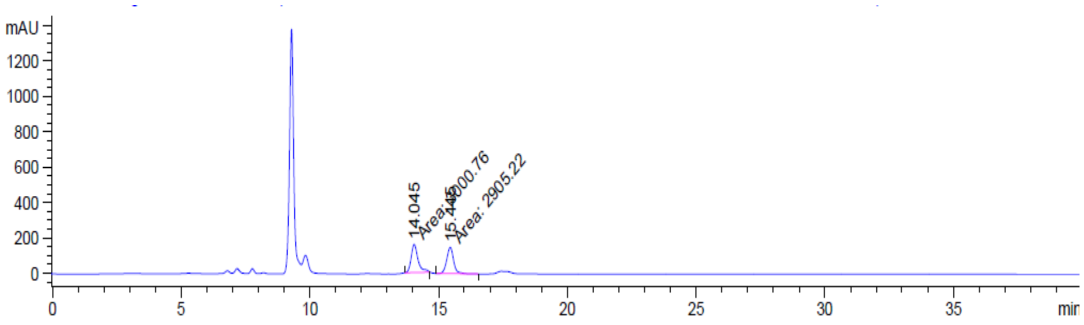

Peak #	RetTime [min]	Type	Width [min]	Area [mAU*s]	Height [mAU]	Area %
1	14.045	MM	0.3129	3000.76074	159.82011	50.8088
2	15.445	MM	0.3236	2905.22266	149.64888	49.1912

Totals : 5905.98340 309.46899

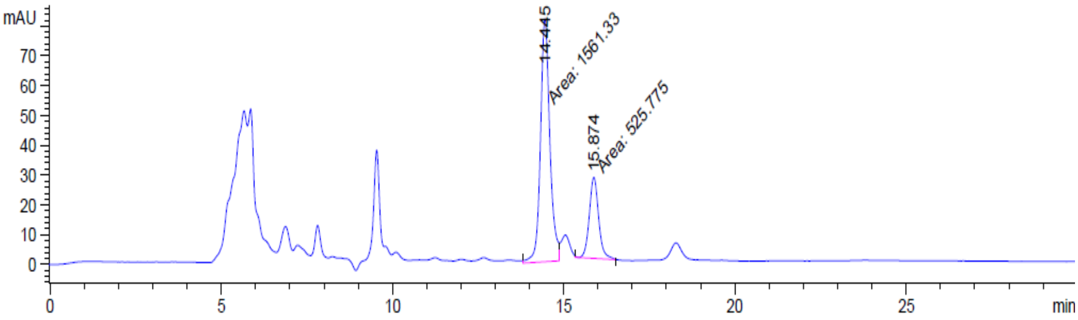

Peak #	RetTime [min]	Type	Width [min]	Area [mAU*s]	Height [mAU]	Area %
1	14.445	MM	0.3186	1561.32837	81.67506	74.8084
2	15.874	MM	0.3207	525.77478	27.32699	25.1916

Totals : 2087.10315 109.00204

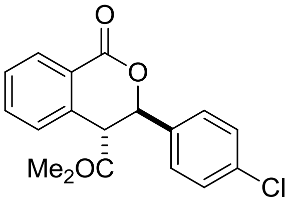

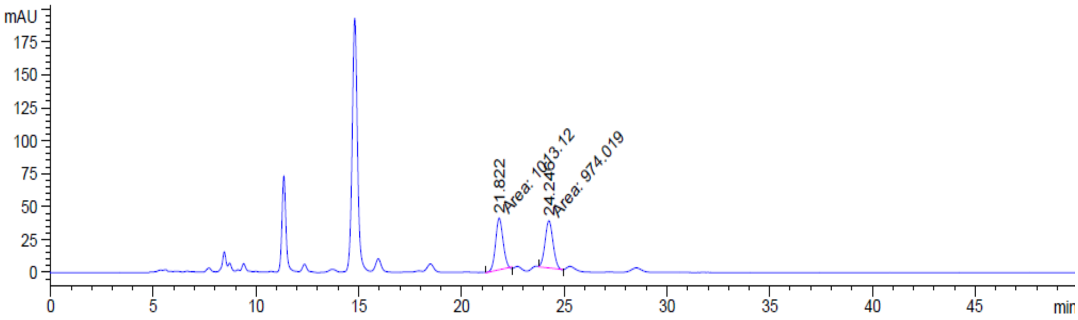

Peak #	RetTime [min]	Type	Width [min]	Area [mAU*s]	Height [mAU]	Area %
1	21.822	MM	0.4273	1013.11859	39.51706	50.9838
2	24.246	MM	0.4509	974.01855	35.99956	49.0162

Totals : 1987.13715 75.51662

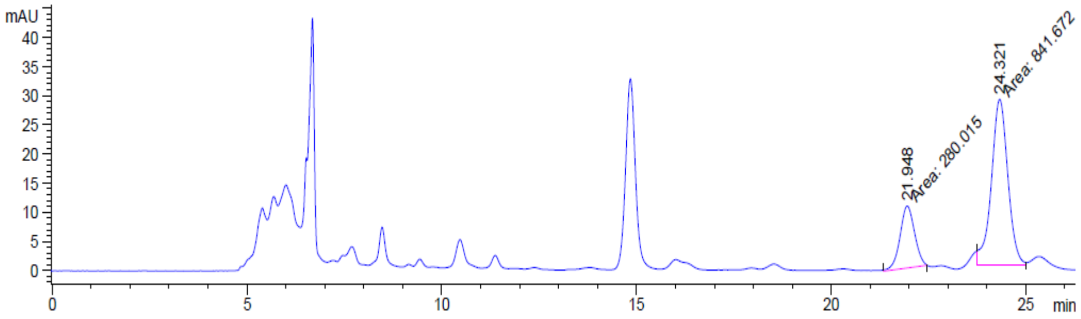

Peak #	RetTime [min]	Type	Width [min]	Area [mAU*s]	Height [mAU]	Area %
1	21.948	MM	0.4368	280.01477	10.68545	24.9637
2	24.321	MM	0.4917	841.67224	28.52824	75.0363

Totals : 1121.68701 39.21369

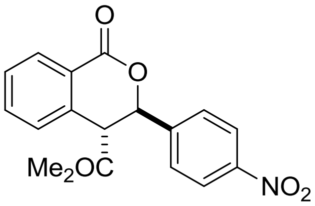

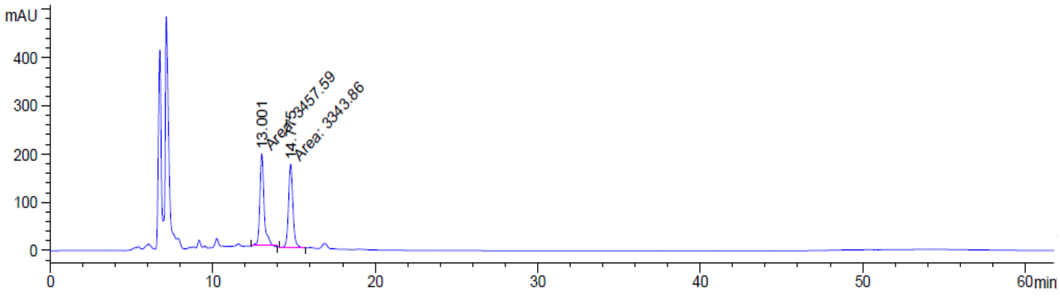

Peak #	RetTime [min]	Type	Width [min]	Area [mAU*s]	Height [mAU]	Area %
1	13.001	MM	0.3028	3457.58545	190.29811	50.8360
2	14.775	MM	0.3219	3343.86450	173.14705	49.1640

Totals : 6801.44995 363.44516

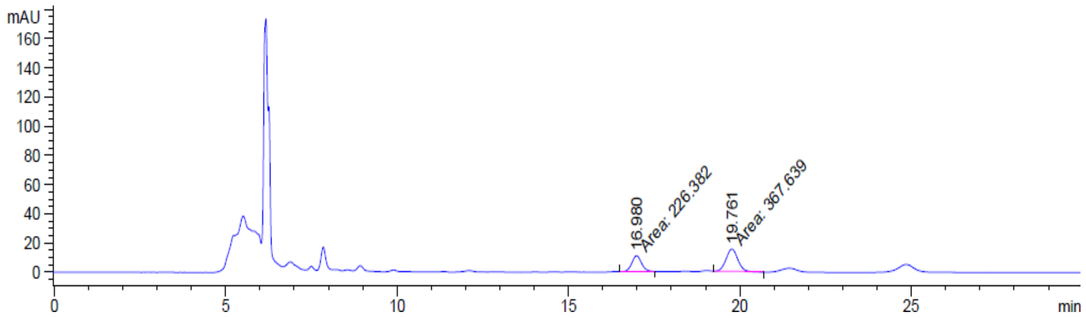

Peak #	RetTime [min]	Type	Width [min]	Area [mAU*s]	Height [mAU]	Area %
1	16.980	MM	0.3410	226.38203	11.06535	38.1101
2	19.761	MM	0.3981	367.63889	15.39081	61.8899

Totals : 594.02092 26.45616

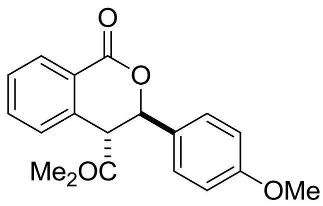

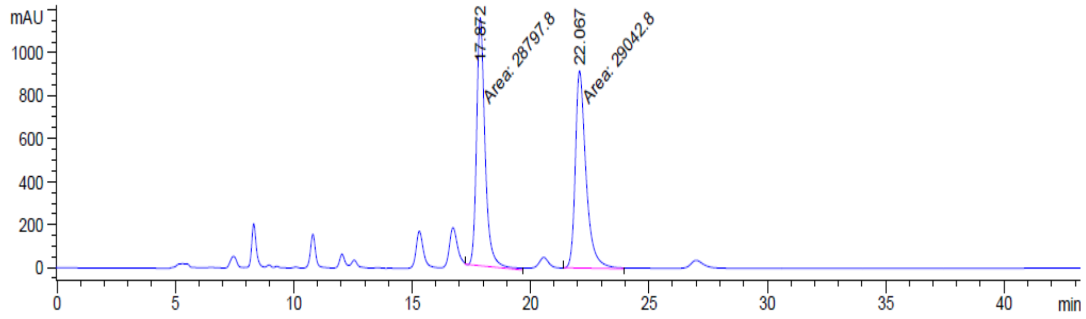

Peak #	RetTime [min]	Type	Width [min]	Area [mAU*s]	Height [mAU]	Area %
1	17.872	MM	0.4163	2.87978e4	1152.94409	49.7882
2	22.067	MM	0.5273	2.90428e4	918.01263	50.2118

Totals : 5.78406e4 2070.95673

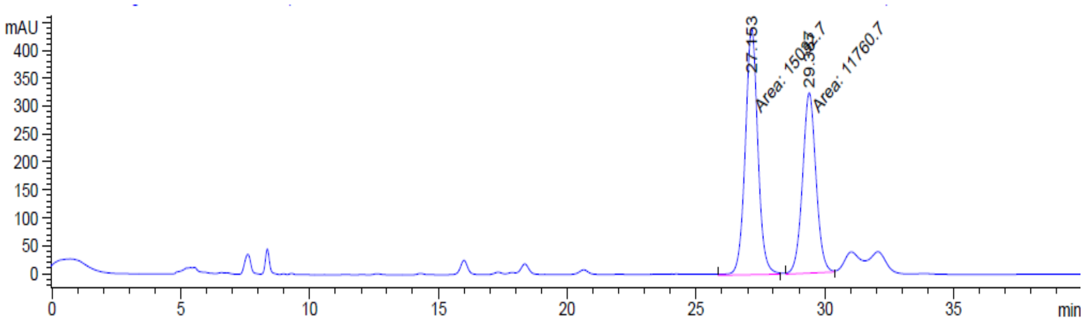

Peak #	RetTime [min]	Type	Width [min]	Area [mAU*s]	Height [mAU]	Area %
1	27.153	MM	0.5681	1.50327e4	441.02301	56.1059
2	29.387	MM	0.6069	1.17607e4	322.97687	43.8941

Totals : 2.67934e4 763.99988

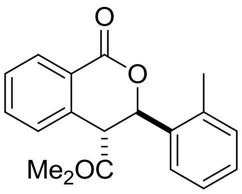

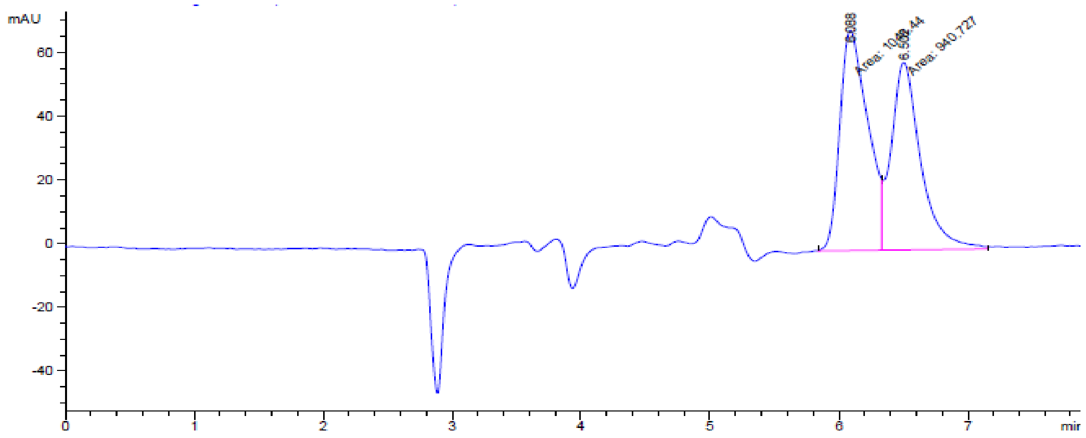

Peak #	RetTime [min]	Type	Width [min]	Area mAU *s	Height [mAU]	Area %
1	6.088	MF	0.2525	1046.43726	69.06831	52.6598
2	6.502	FM	0.2668	940.72656	58.77325	47.3402

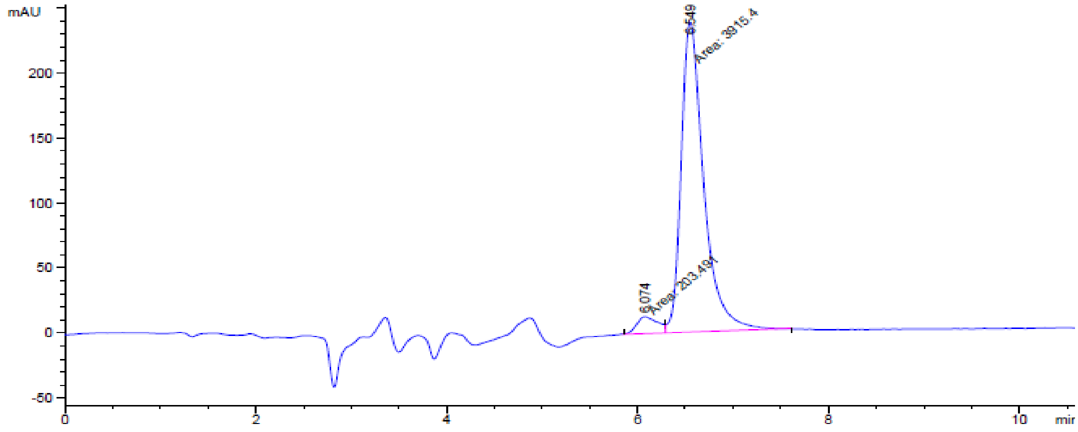

Peak #	RetTime [min]	Type	Width [min]	Area mAU *s	Height [mAU]	Area %
1	6.074	MF	0.2609	203.49100	13.00128	4.9404
2	6.549	FM	0.2740	3915.39722	238.15657	95.0596

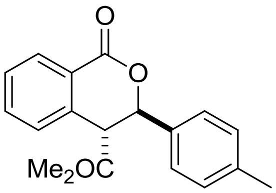

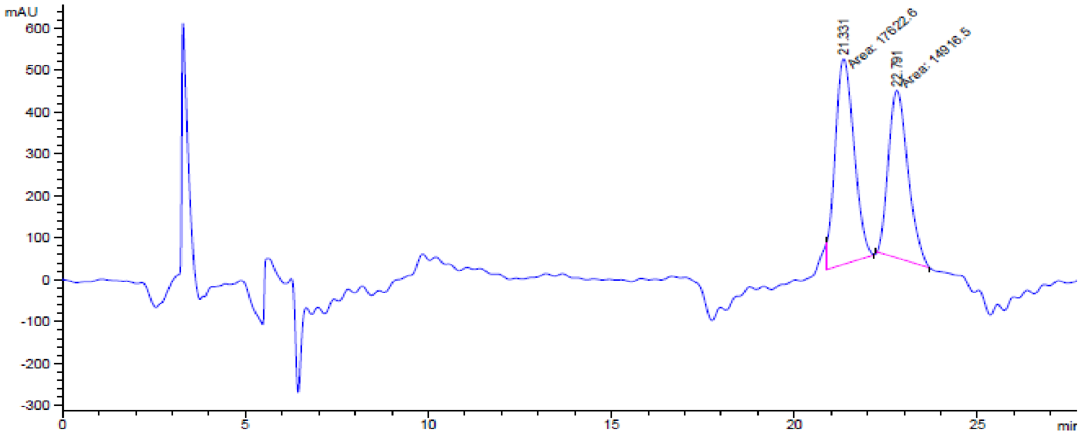

Peak #	RetTime [min]	Type	Width [min]	Area mAU *s	Height [mAU]	Area %
1	21.331	FM T	0.5994	1.76226e4	490.01767	54.1582
2	22.791	MM T	0.6224	1.49165e4	399.41150	45.8418

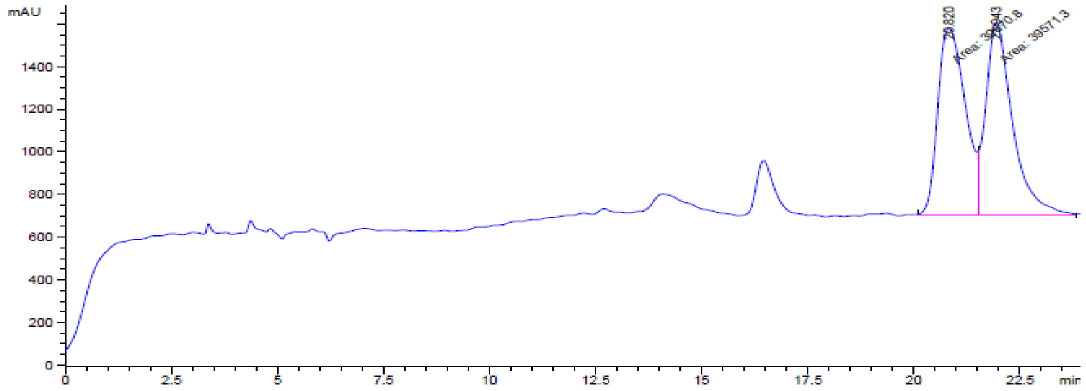

Peak #	RetTime [min]	Type	Width [min]	Area mAU *s	Height [mAU]	Area %
1	20.820	MF	0.7594	3.98708e4	875.06567	50.1885
2	21.943	FM	0.7315	3.95713e4	901.60907	49.8115

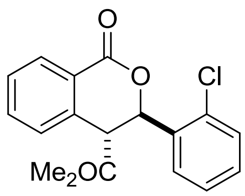

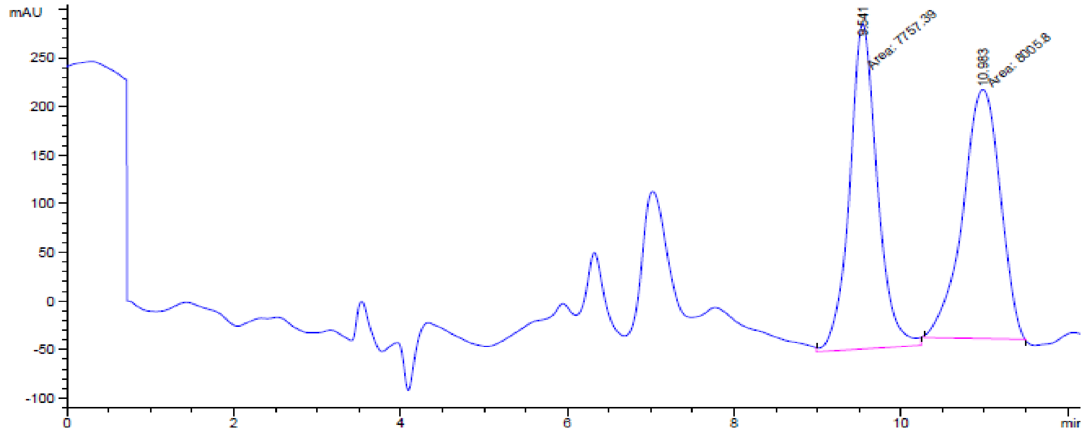

Peak #	RetTime [min]	Type	Width [min]	Area mAU *s	Height [mAU]	Area %
1	9.541	MM	0.3869	7757.39063	334.16409	49.2121
2	10.983	MM	0.5209	8005.79736	256.14786	50.7879

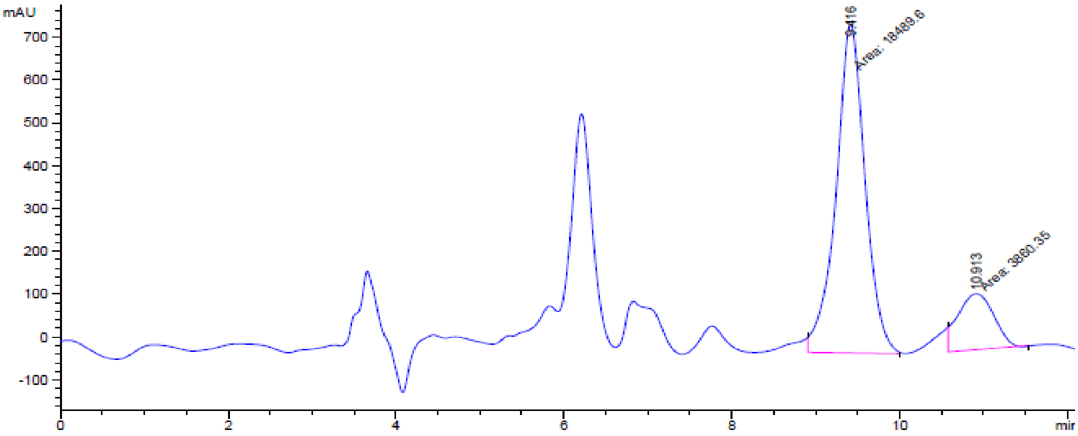

Peak #	RetTime [min]	Type	Width [min]	Area mAU *s	Height [mAU]	Area %
1	9.416	FM T	0.4015	1.84896e4	767.50299	82.7277
2	10.913	FM	0.4921	3860.35132	130.75725	17.2723

Synthesis of catalyst 24sq

An oven-dried 25 mL reaction vessel containing a stirring bar under argon atmosphere was charged with 3,4-diethoxycyclobut-3-ene-1,2-dione (2 g, 12 mmol, 1eq) and 3,5-bis(trifluoromethyl)aniline 2.8 g (12.3 mmol, 1.05 eq) followed by the addition of 8 mL EtOH (4 vol, 1.5M final solution) and the reaction mixture was stirred for 20 h at room temperature. The resulting suspension was filtered and washed with (2 x 10 mL) of chilled EtOH to give the 3-((3,5-bis(trifluoromethyl)phenyl)amino)-4-ethoxycyclobut-3-ene-1,2-dione intermediate.

An oven-dried 25 mL reaction vessel containing a stirring bar under argon atmosphere was charged with 3-((3,5-bis(trifluoromethyl)phenyl)amino)-4-ethoxycyclobut-3-ene-1,2-dione 60 mg (0,17 mmol, 1 eq) and (*S*)-isobutyl BIMAH 32 mg (0.17 mmol, 1 eq). The two solids were dissolved by addition of 1 mL EtOH (resulting solution 0.2M) and the reaction mixture was stirred for 20 h at room temperature. At completion, the solvent was removed in vacuo and 1 mL of TBME was added resulting in the formation of yellow solid. The solid was filtered and washed with (2 x 3 mL) *n*-hexane.

^{1}H NMR (600 MHz, DMSO-d_6) δ ppm 10.27 (s, 1H), 8.38 (s, 1H), 8.05 (d, J = 16.1 Hz, 2H), 7.62 (s, 2H), 7.49 (s, 1H), 7.18 (s, 2H), 5.63 (d, J = 85.2 Hz, 1H), 2.04 – 1.95 (m, 1H), 1.91 (d, J = 6.2 Hz, 1H), 1.65 (tt, J = 13.3, 6.6 Hz, 1H), 0.93 (dt, J = 17.0, 8.6 Hz, 6H).

^{13}C NMR (151 MHz, DMSO-d_6) δ ppm 184.82, 181.00, 169.24, 163.22, 154.38, 143.24, 141.31, 134.48, 131.72, 124.51, 122.84, 122.71, 122.01, 120.94, 119.19, 118.56, 115.34, 111.99, 51.57, 45.19, 24.74, 23.07, 22.27.

Synthesis of catalyst 26aS - General procedure for thiourea/BIMAH organocatalysts

An oven-dried 25 mL reaction vessel containing a stirring bar under argon atmosphere was charged with 1-isothiocyanato-3,5-bis(trifluoromethyl)benzene 60 mg (2.2 mmol, 1.05 eq) and (*S*)-1-(1H-benzo[d]imidazol-2-yl)-2-methylpropan-1-amine 40mg (0.21 mmol, 1 eq). The two solids were dissolved by the addition of 0.4 mL DCM (resulting solution 0.5 M) and the reaction mixture was stirred for 20 h at room temperature. At completion, the solvent was removed in vacuo and 0.5 mL of THF was added resulting in the formation of a yellow solid. The solid was filtered and washed with (2 x 0.5mL) n-hexane.

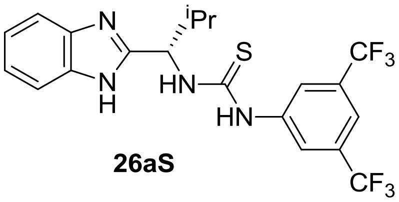

26aS

^{1}H NMR (600 MHz, $CDCl_3$):δ ppm 1.15 (br d, *J*=6.05 Hz, 3 H) 1.20 - 1.29 (m, 3 H) 2.51 (dq, *J*=13.41, 6.59 Hz, 1 H) 5.73 - 5.84 (m, 1 H) 7.21 (br dd, *J*=5.59, 2.66 Hz, 2 H) 7.32 (br s, 1 H) 7.52 (br s, 3 H) 8.91 - 9.26 (m, 1 H) 9.73 - 10.16 (m, 1 H)

^{13}C-NMR (151 MHz, $CDCl_3$): δ ppm 153.98, 140.13, 131.76, 126.02, 122.88, 121.64, 121.50, 118.33, 118.02, 114.21, 58.26, 19.49, 19.36

HRMS (ESI): $[MH]^+$ calculated for $C_{20}H_{18}F_6N_4S$ requires 461.1229; found 461.1221

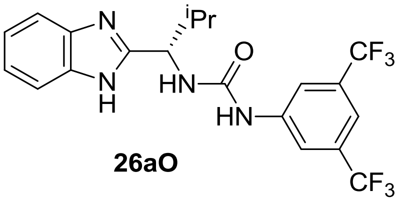

26aO

^{1}H NMR (400 MHz, DMSO-d_6): δ ppm 9.71 (s, 1H), 8.03 (s, 2H), 7.61 (m, 2H), 7.58 (s, 1H), 7.29 (m, 2H), 7.22 (d, J = 7.5 Hz, 1H), 4.96 – 4.90 (m, 1H), 2.34 (m, 1H), 0.94 (t, J = 6.3 Hz, 6H).

^{13}C-NMR (101 MHz, DMSO-d_6): δ ppm 155.18, (s, 1 C), 142.60 (s, 1 C), 131.26 (s, 1 C), 131.05 (s, 1 C), 125.10 (s, 1 C), 123.32 (s, 1 C), 122.39 (s, 1 C), 117.59 (s, 1 C), 55.12 (s, 1 C), 53.67 (s, 1 C), 32.62 (s, 1 C), 19.41(s, 1 C), 18.17 (s, 1 C)

HRMS (ESI): $[MH]^+$ calculated for $C_{20}H_{19}F_6N_4O$ requires 445.1458; found 445.1462

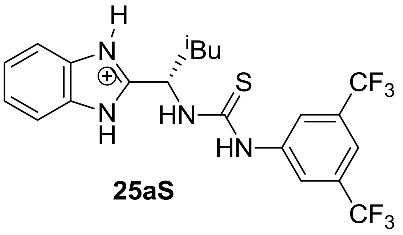

25aS

^{1}H NMR (400 MHz, DMSO-d_6) δ ppm 12.38 (s, 1H), 9.37 (s, 1H), 8.06 (s, 2H), 7.56 (s, 2H), 7.46 (d, J = 7.3 Hz, 1H), 7.21 – 7.10 (m, 2H), 7.05 (d, J = 8.3 Hz, 1H), 5.07 (dd, J = 14.7, 8.3 Hz, 1H), 1.81 (qd, J = 13.7, 8.2 Hz, 2H), 1.69 – 1.55 (m, 1H), 0.93 (d, J = 6.5 Hz, 6H).

HRMS (ESI): $[MH]^+$ calculated for $C_{21}H_{21}F_6N_4O$ requires 475.1386; found 475.1389

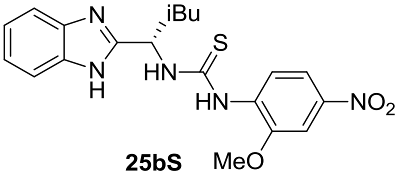

25bS

^{1}H NMR (600 MHz, $CDCl_3$): δ ppm 8.52 (d, J = 18.4 Hz, 2H), 7.87 (d, J = 24.1 Hz, 2H), 7.62 – 7.42 (m, 3H), 7.22 (d, J = 13.6 Hz, 2H), 6.81 (s, 1H), 5.09 (s, 1H), 3.85 – 3.72 (m, 3H), 1.83 – 1.60 (m, 3H), 1.10 – 1.03 (m, 6H).

HRMS (ESI): $[MH]^+$ calculated for $C_{20}H_{24}N_5O_3S$ requires 414.1594; found 414.1598

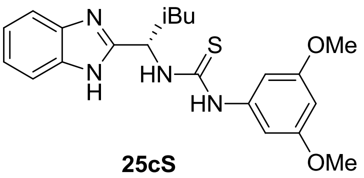

25cS

^{1}H NMR (600 MHz, $CDCl_3$): δ ppm 7.63 (s, 1H), 7.53 (s, 1H), 7.27 – 7.19 (m, 3H), 6.66 (s, 1H), 6.61 – 6.57 (m, 2H), 6.44 (s, 1H), 5.98 (s, 1H), 5.09 (s, 1H), 3.85 – 3.81 (m, 6H), 1.69 – 1.62 (m, 3H), 1.12-1.01 (m, 6H).

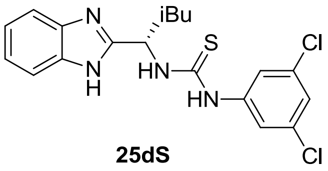

25dS

^{1}H NMR (600 MHz, $CDCl_3$): δ ppm 9.09 (d, J = 110.2 Hz, 2H), 7.53 (dd, J = 5.7, 3.1 Hz, 2H), 7.31 (dt, J = 14.4, 7.2 Hz, 2H), 7.19 (s, 1H), 7.05 (s, 1H), 5.93 (s, 1H), 5.30 (s, 1H), 2.18- 1.94 (m, 2H), 1.91 (dd, J = 26.1, 18.9 Hz, 2H), 0.85 (dd, J = 18.2, 11.4 Hz, 6H).

HRMS (ESI): $[MH]^+$ calculated for $C_{19}H_{21}Cl_2N_4S$ requires 407.0858; found 407.0855

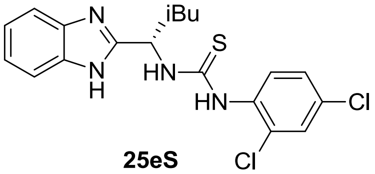

25eS

^{1}H NMR (600 MHz, DMSO-d_6): δ ppm 9.57 (s, 1H), 8.84 (d, J = 6.5 Hz, 1H), 7.85 (d, J = 8.7 Hz, 2H), 7.67 – 7.55 (m, 3H), 7.35 (ddd, J = 8.6, 7.2, 2.6 Hz, 3H), 5.81 (d, J = 6.7 Hz, 1H), 2.01 – 1.89 (m, 2H), 1.74 (dt, J = 13.4, 6.6 Hz, 1H), 0.98 (dd, J = 14.1, 6.8 Hz, 6H).

HRMS (ESI): $[MH]^+$ calculated for $C_{19}H_{21}Cl_2N_4S$ requires 407.0858; found 407.0863

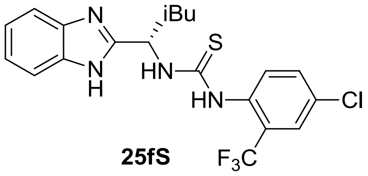

25fS

^{1}H NMR (600 MHz, $CDCl_3$): δ ppm 7.63 (s, 1H), 7.53 (s, 1H), 7.27 – 7.19 (m, 3H), 6.66 (s, 1H), 6.61 – 6.57 (m, 2H), 6.44 (s, 1H), 5.98 (s, 1H), 5.09 (s, 1H), 3.85 – 3.81 (m, 6H), 1.66 (t, J = 8.2 Hz, 3H), 1.12 – 1.00 (m, 6H).

HRMS (ESI): $[MH]^+$ calculated for $C_{20}H_{21}ClF_3N_4S$ requires 441.1122; found 405.1128

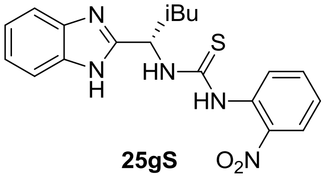

25gS

^{1}H NMR (600 MHz, $CDCl_3$): δ ppm 8.48 (s, 1H), 8.19 (s, 1H), 7.69 (s, 1H), 7.65 – 7.44 (m, 3H), 7.41 (s, 1H), 7.24 (d, J = 10.0 Hz, 2H), 7.19 (s, 1H), 7.12 (s, 1H), 5.09 (s, 1H), 2.10 (s, 1H), 1.80 (s, 1H), 1.68 (s, 1H), 1.13 – 1.00 (m, 6H).

HRMS (ESI): $[MH]^+$ calculated for $C_{19}H_{22}N_5O_2S$ requires 384.1489;found 383.1487

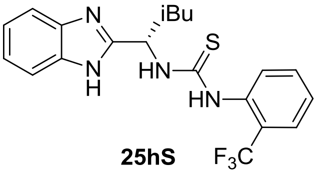

25hS

^{1}H NMR (600 MHz, DMSO-d_6): δ ppm 9.35 (s, 1H), 8.64 (d, J = 7.3 Hz, 1H), 7.68 (dd, J = 17.3, 6.5 Hz, 1H), 7.63 (dd, J = 10.1, 4.3 Hz, 3H), 7.44 (t, J = 6.9 Hz, 1H), 7.30 (dd, J = 5.7, 3.0 Hz, 2H), 6.01 – 5.60 (m, 1H), 2.01 – 1.86 (m, 2H), 1.70 (ddt, J = 20.1, 13.3, 6.7 Hz, 1H), 0.99 – 0.91 (m, 6H).

HRMS (ESI): $[MH]^+$ calculated for $C_{20}H_{22}F_6N_4O$ requires 407.1512; found 406.1422

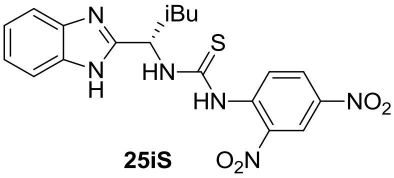

25iS

^{1}H NMR (600 MHz, $CDCl_3$): δ ppm 9.29 (s, 1H), 9.00 (s, 1H), 8.51 (s, 1H), 8.24 (s, 1H), 7.70 (s, 1H), 7.54 (d, J = 26.6 Hz, 2H), 7.22 (d, J = 13.3 Hz, 2H), 6.79 (s, 1H), 5.09 (s, 1H), 1.79 (s, 1H), 1.74 – 1.62 (m, 2H), 1.15 – 0.96 (m, 6H).

HRMS (ESI): $[MH]^+$ calculated for $C_{19}H_{21}N_6O_4S$ requires 429.1340; found 417.1346

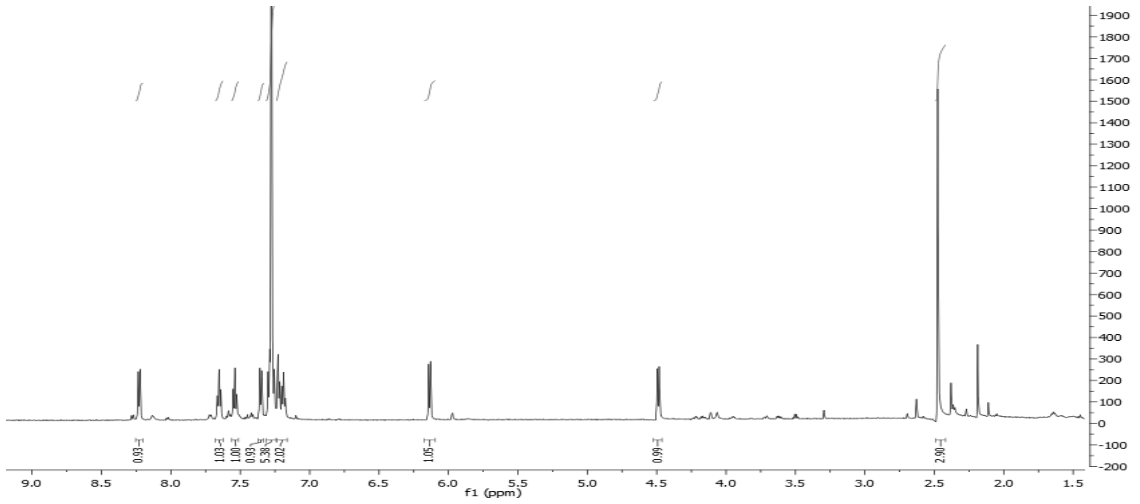

^{1}H NMR spectrum of **6**.

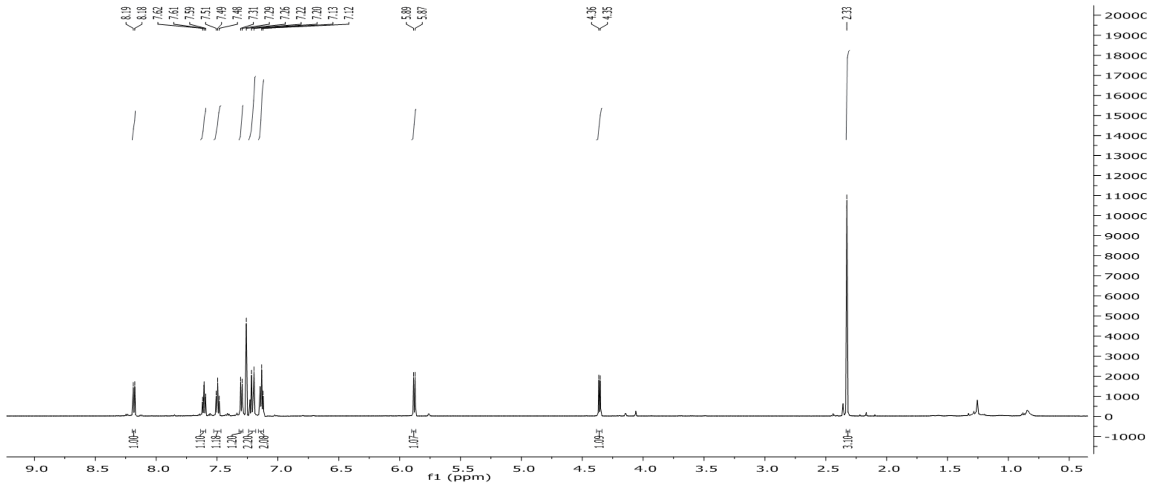

^{1}H NMR spectrum of **7**.

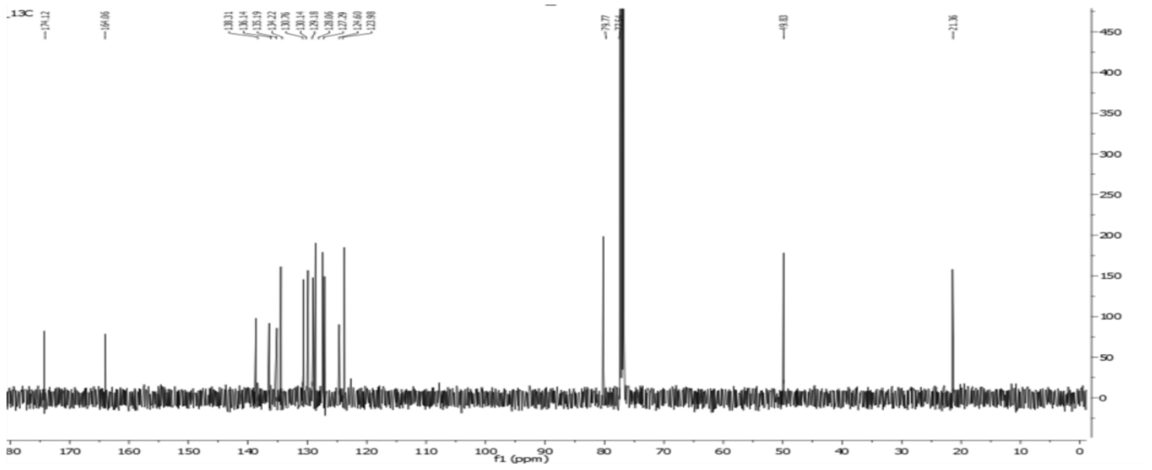

^{13}C NMR spectrum of **7**.

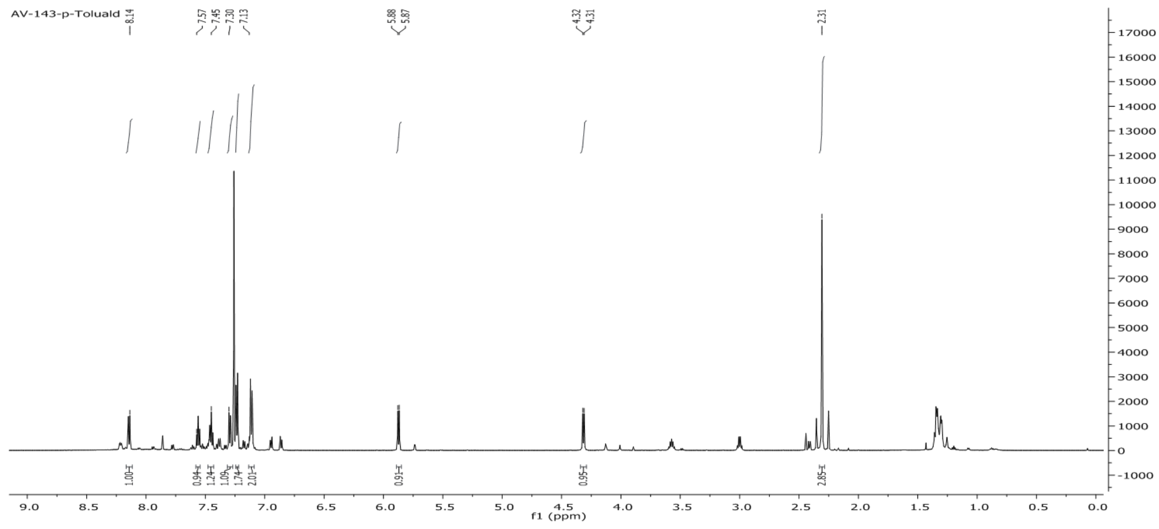

^{1}H NMR spectrum of **8**.

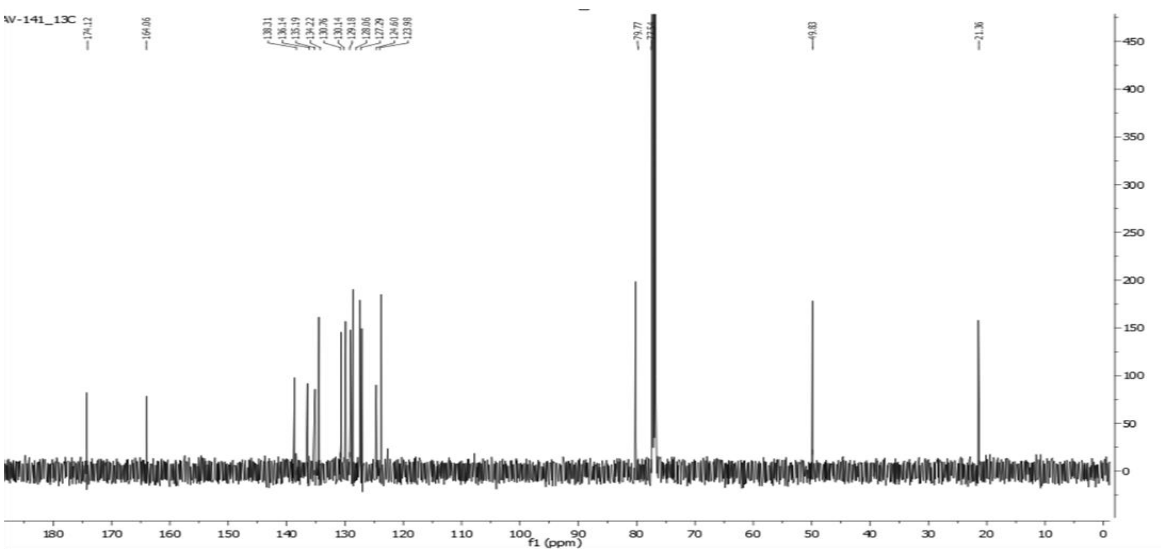

^{13}C NMR spectrum of **8**.

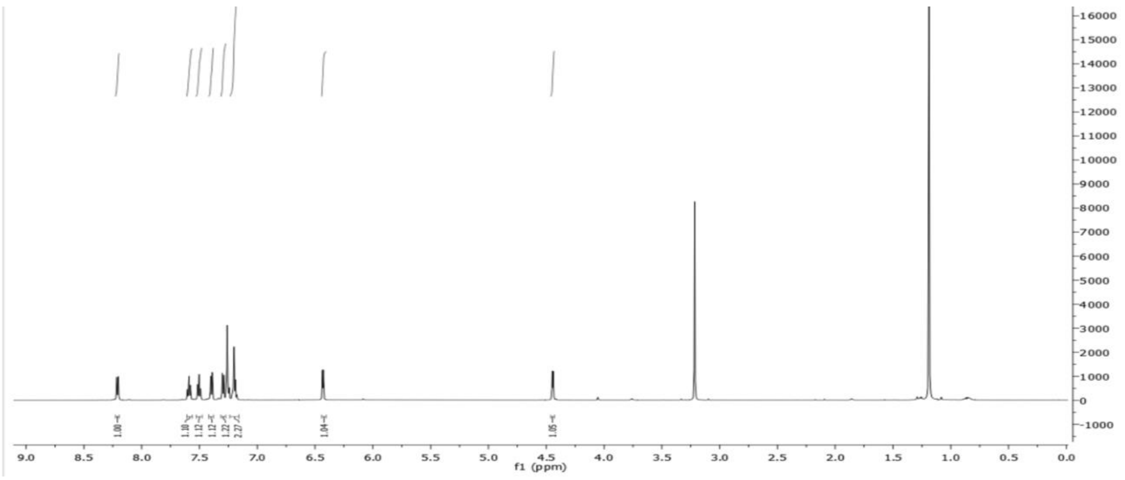

^{1}H NMR spectrum of **9**.

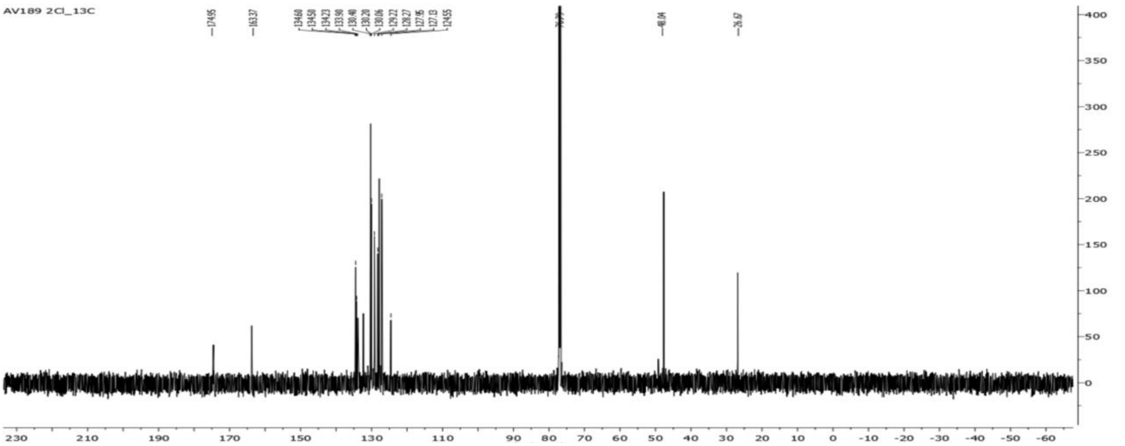

^{13}C NMR spectrum of **9**.

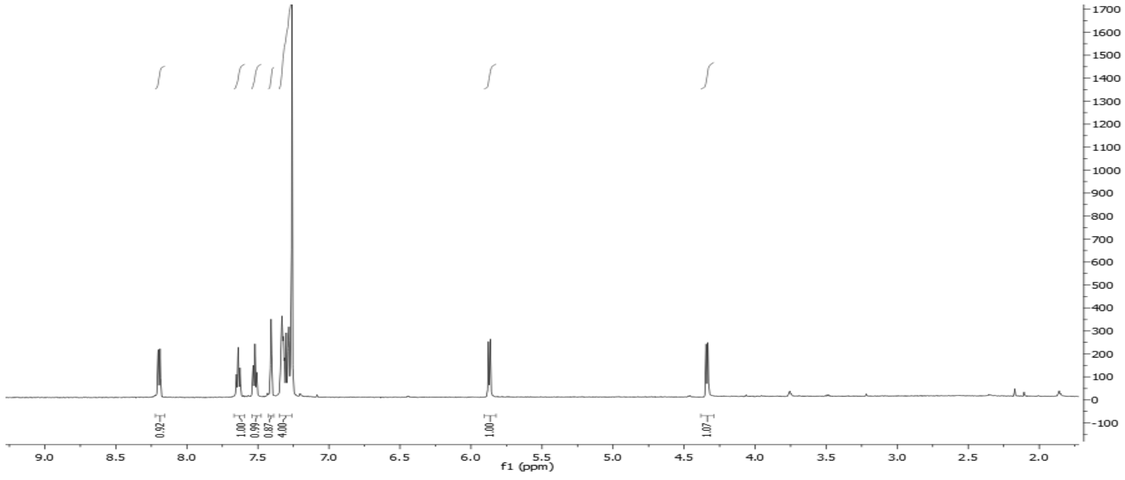

^{1}H NMR spectrum of **10**.

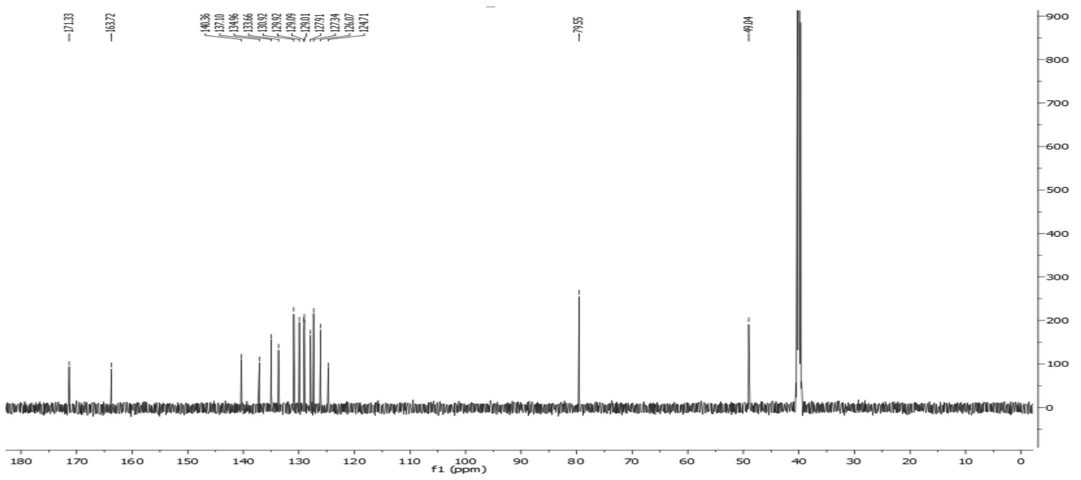

^{13}C NMR spectrum of **10**.

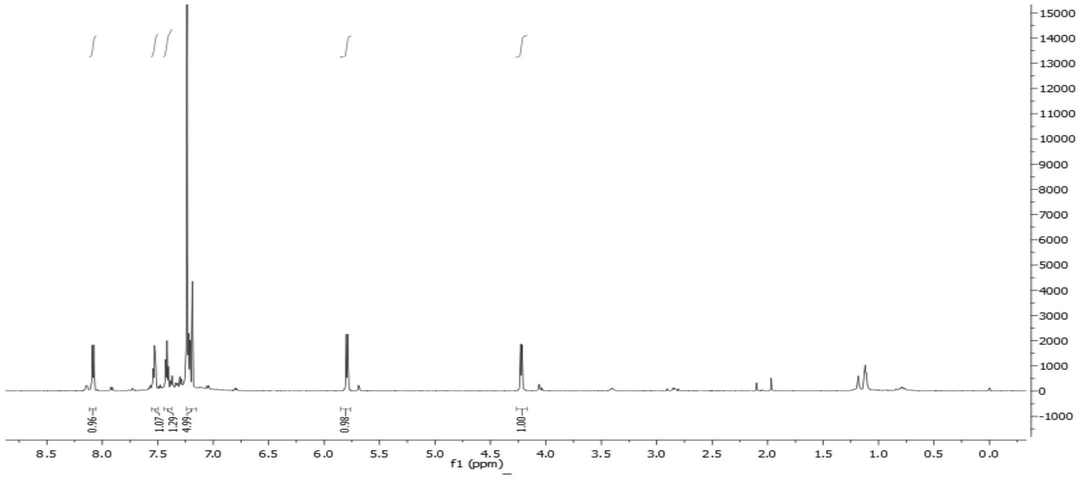

^{1}H NMR spectrum of **11.**

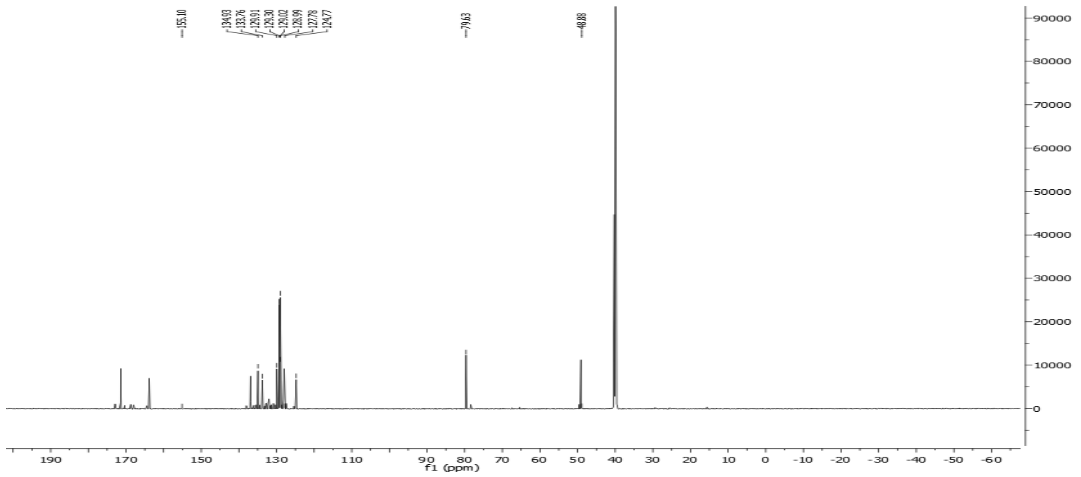

^{13}C NMR spectrum of **11**.

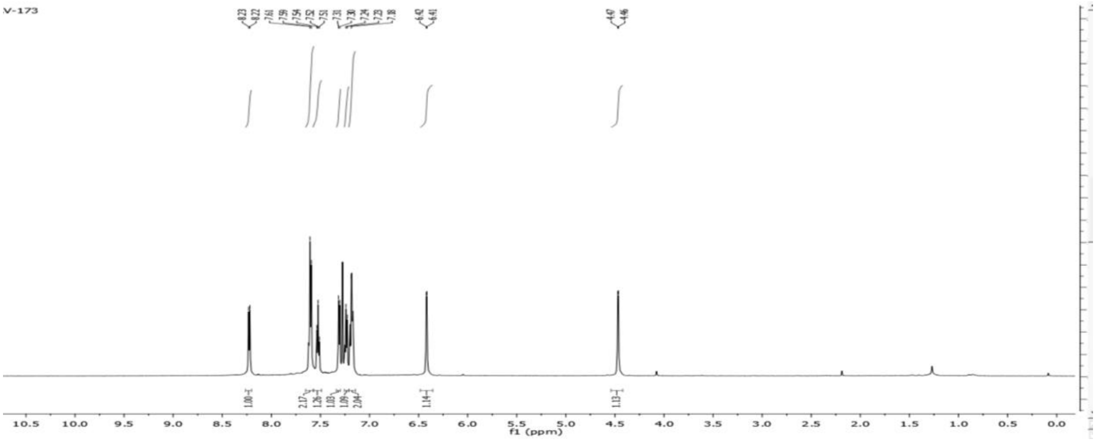

^{1}H NMR spectrum of **12**.

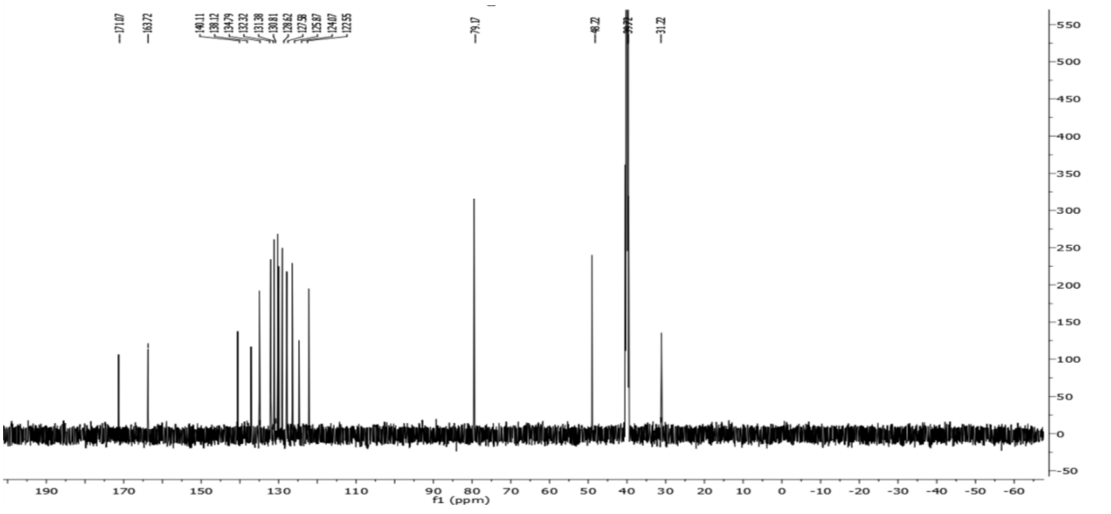

^{13}C NMR spectrum of **12**.

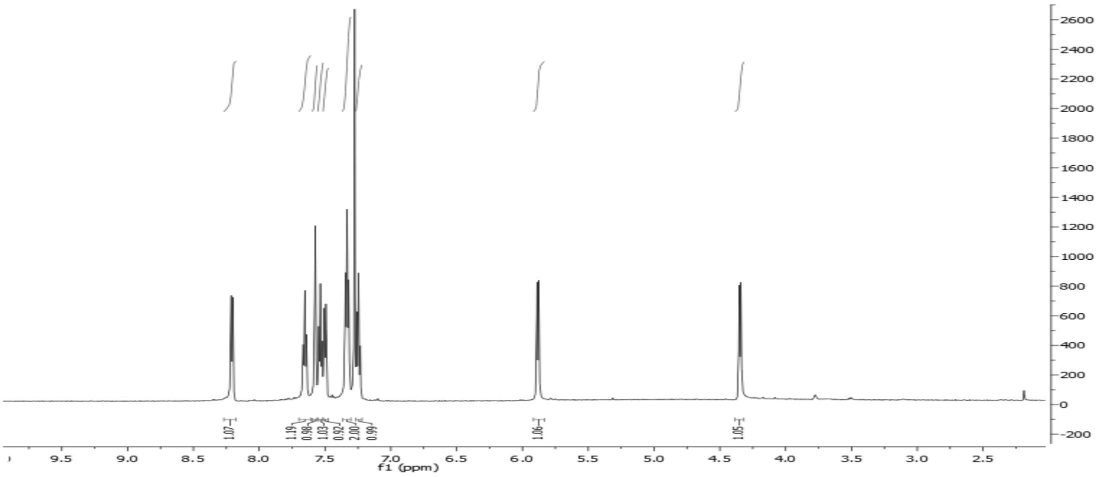

^{1}H NMR spectrum of **13**.

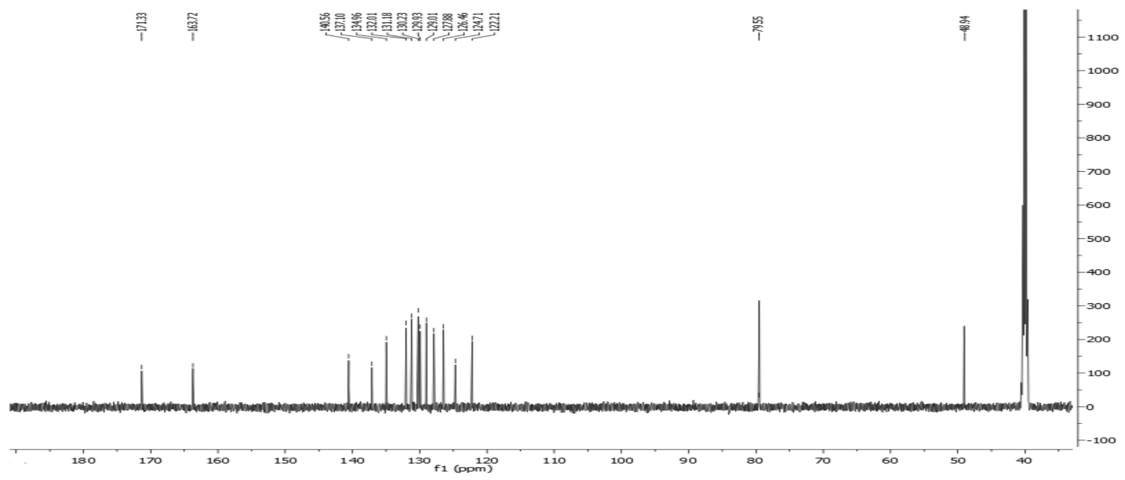

^{13}C NMR spectrum of **13**.

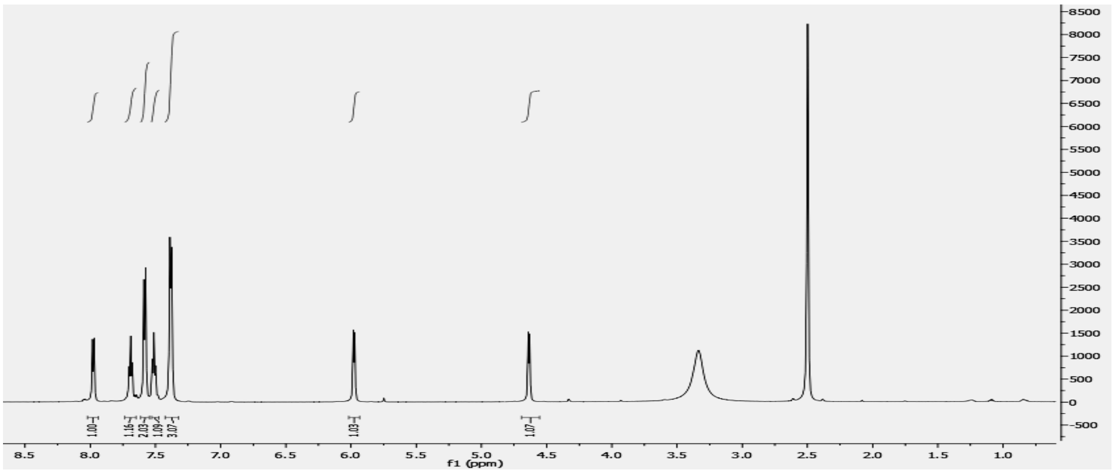

^{1}H NMR spectrum of **14**.

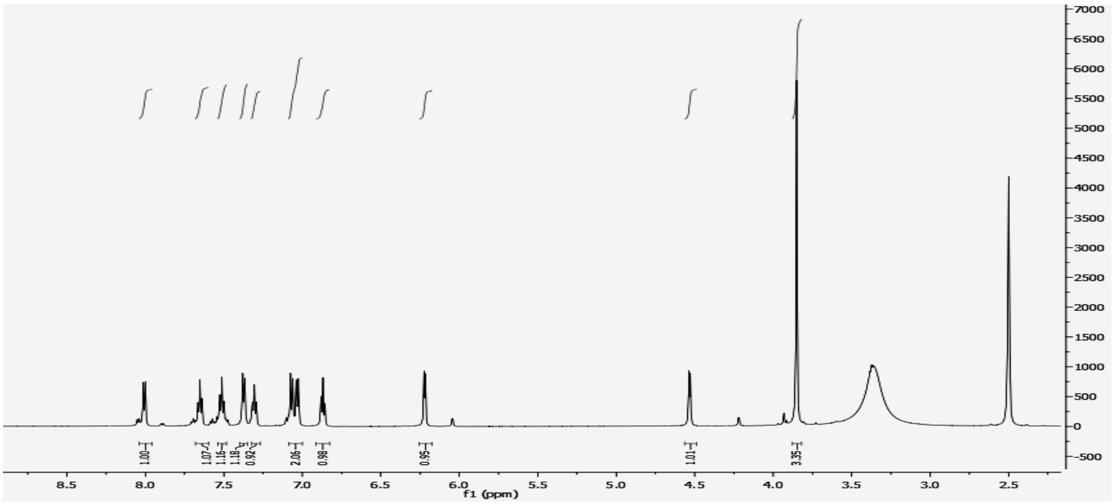

^{1}H NMR spectrum of **15**.

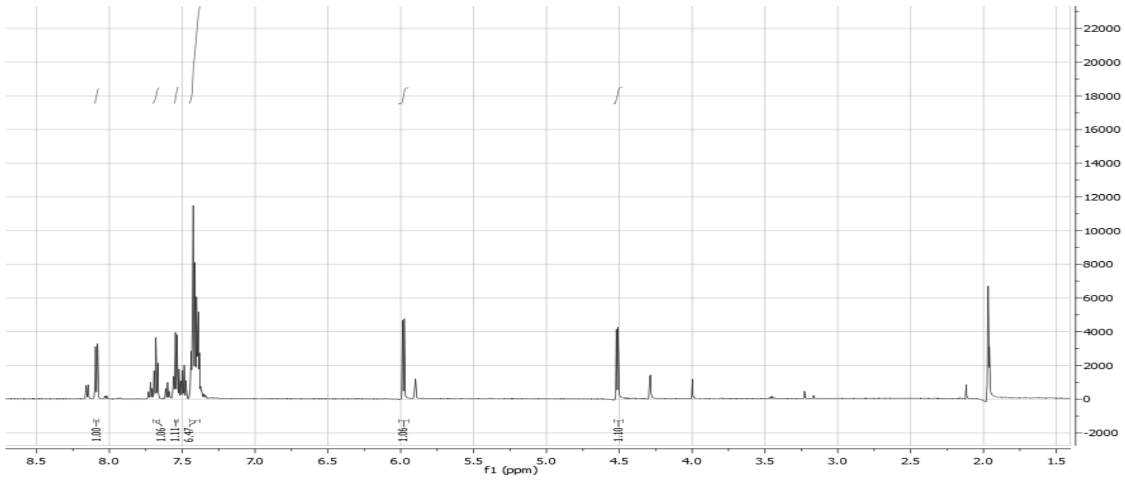

^{1}H NMR spectrum of **16**.

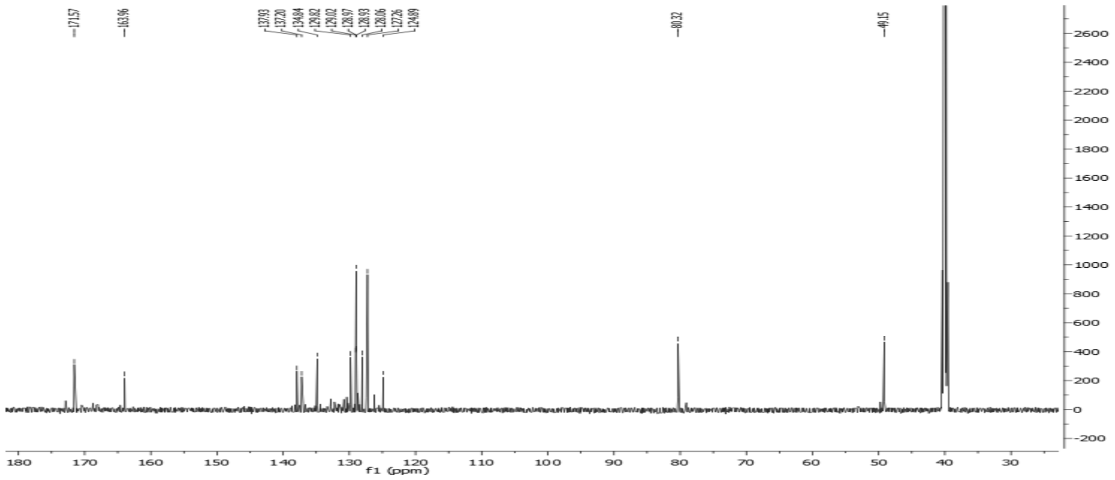

^{13}C NMR spectrum of **16**.

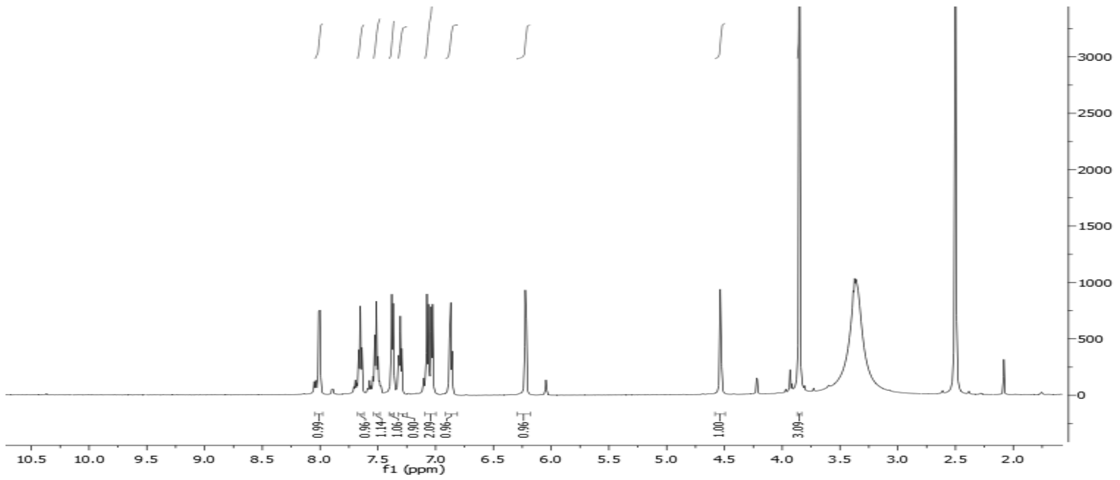

^{1}H NMR spectrum of **17**.

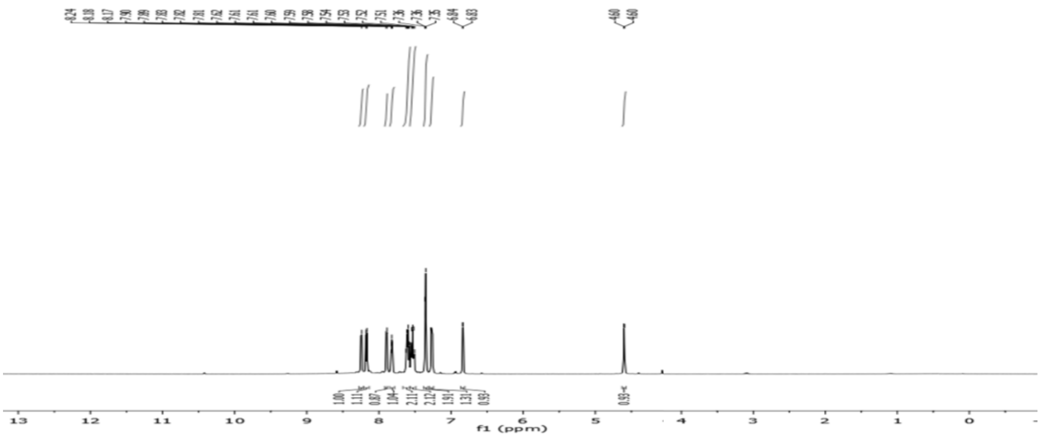

^{1}H NMR spectrum of **18**.

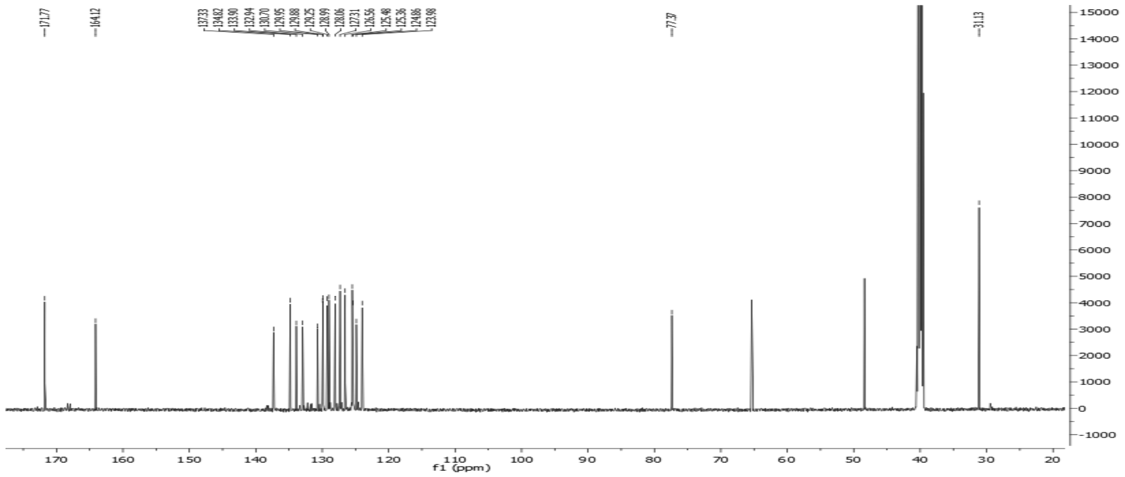

^{13}C NMR spectrum of **18**.

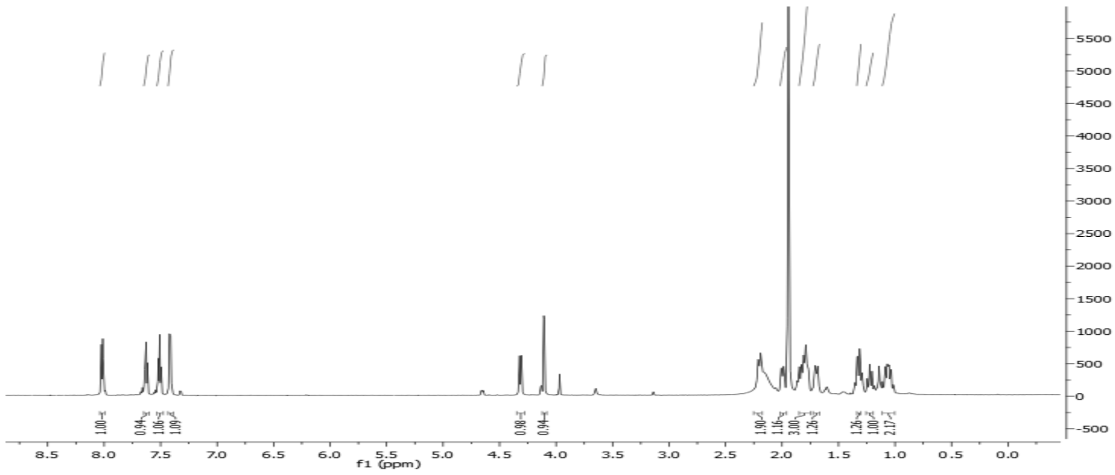

^{1}H NMR spectrum of **19**.

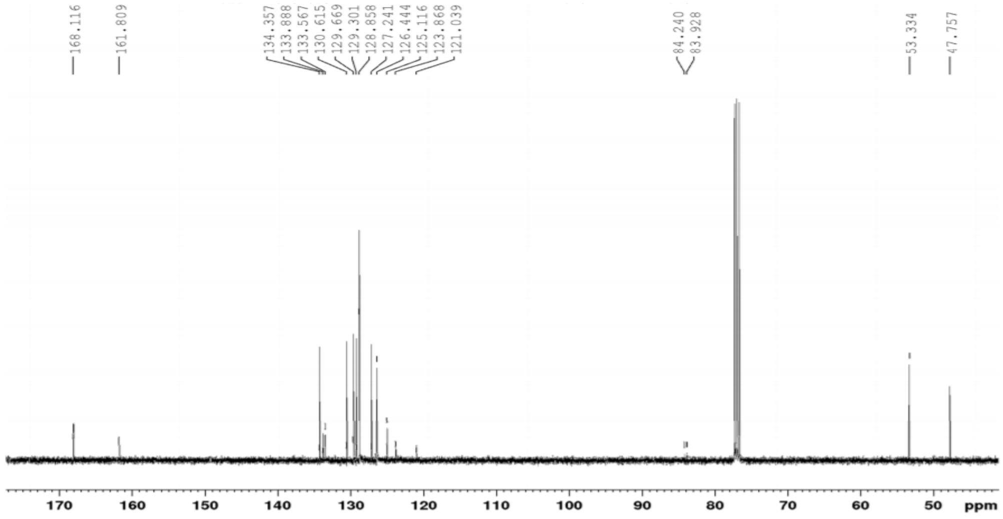

^{13}C NMR spectrum of **19**.

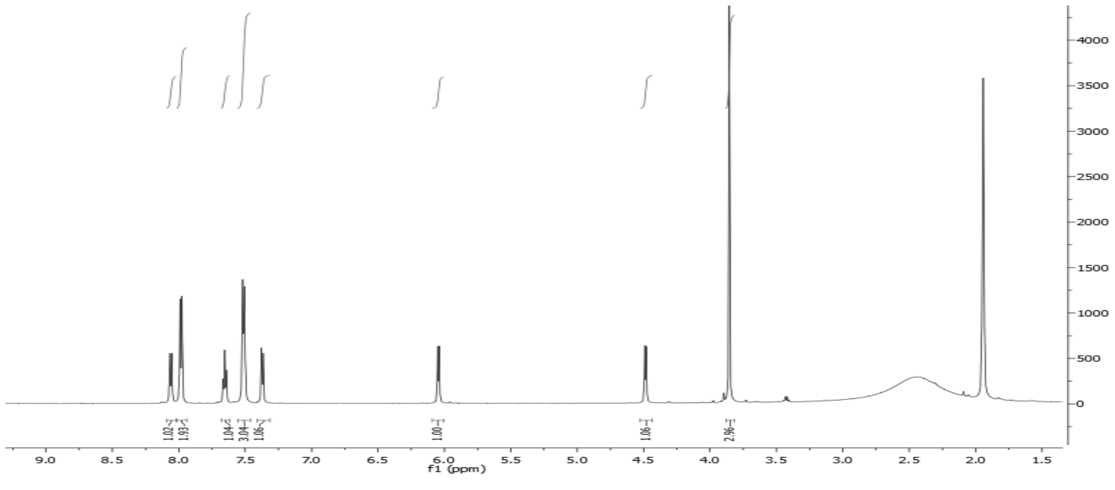

^{1}H NMR spectrum of **20**.

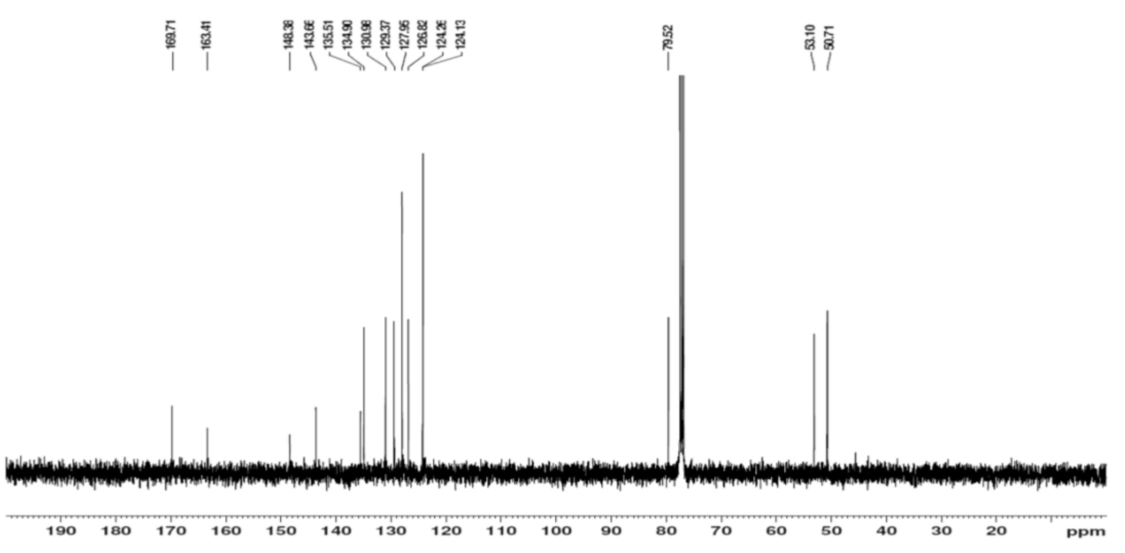

^{13}C NMR spectrum of **20**.

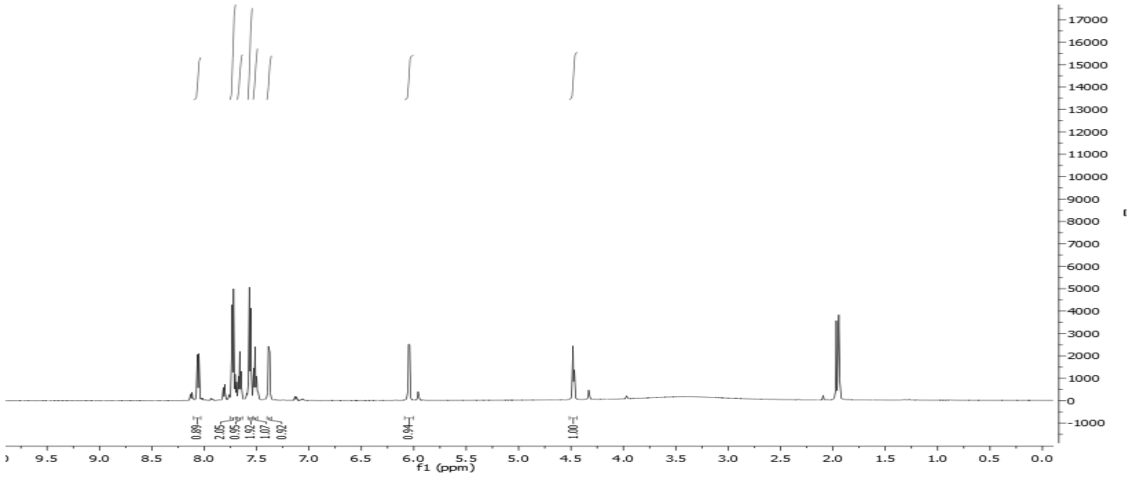

^{1}H NMR spectrum of **21**.

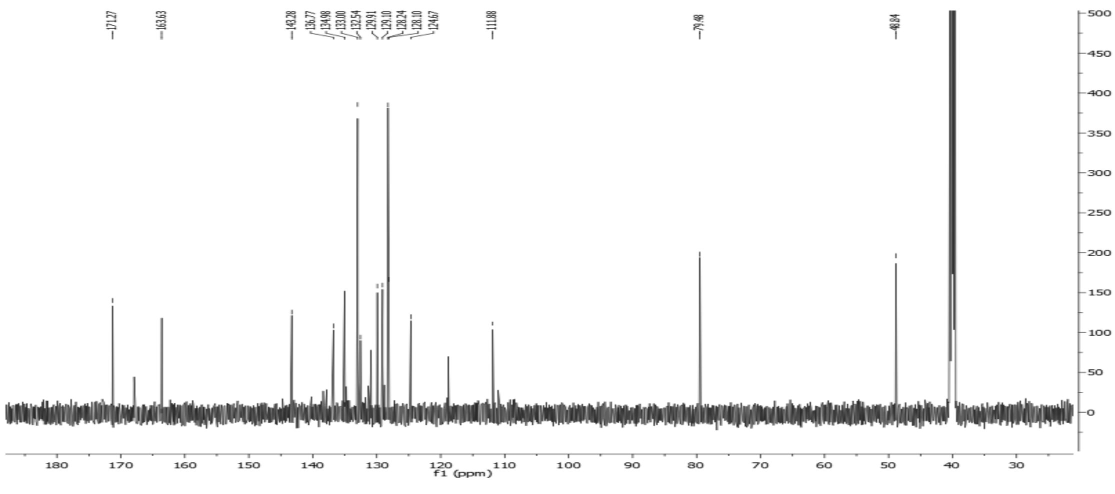

^{13}C NMR spectrum of **21**.

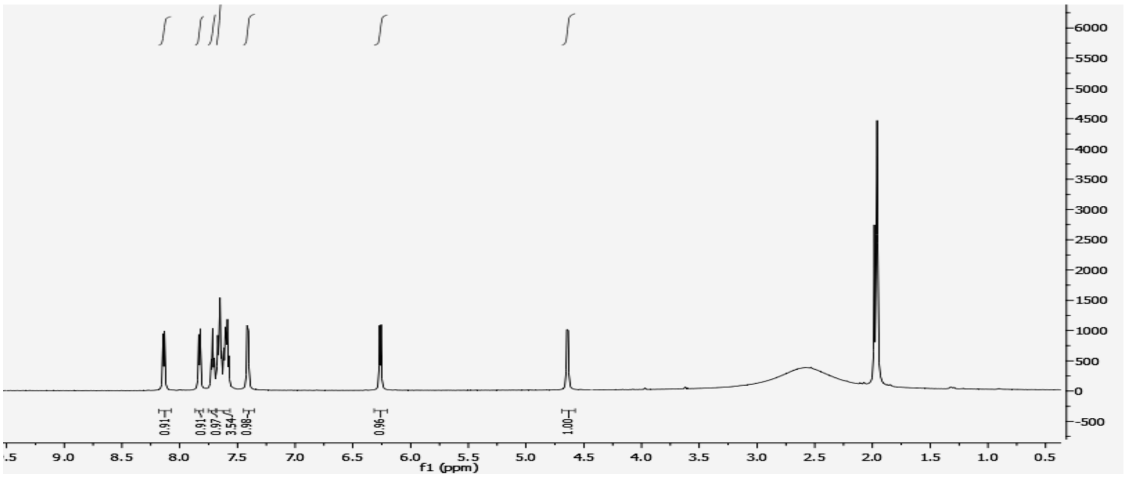

^{1}H NMR spectrum of **22**.

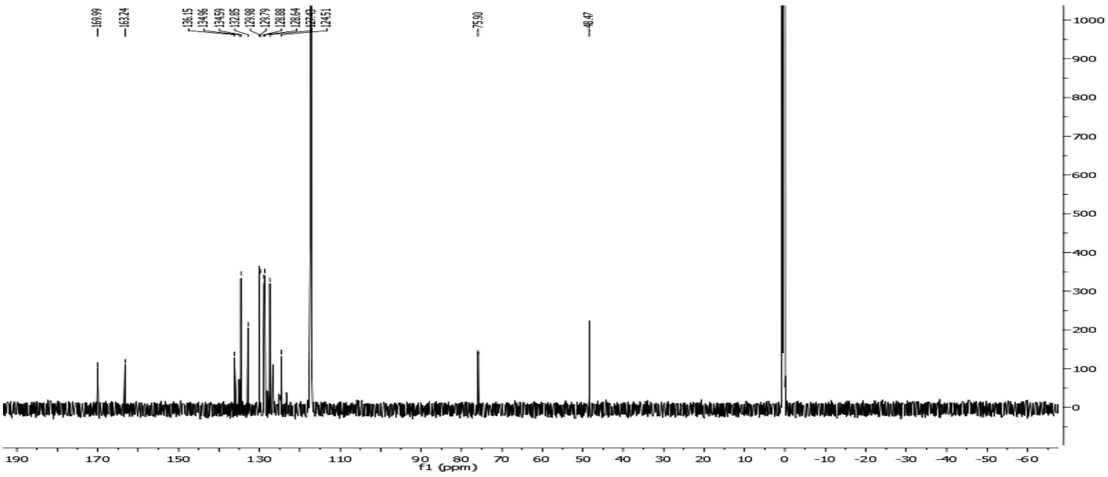

^{13}C NMR spectrum of **22**.

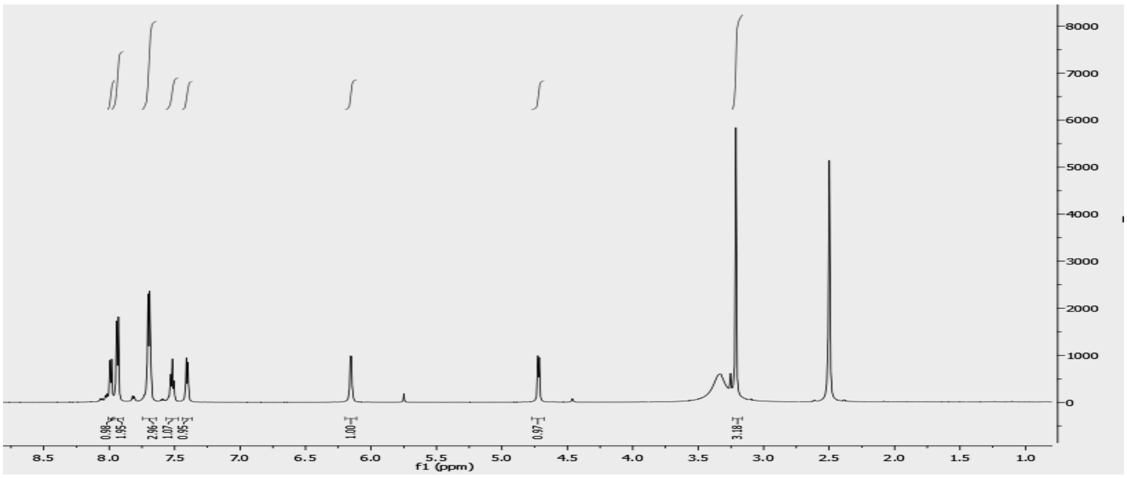

^{1}H NMR spectrum of **23**.

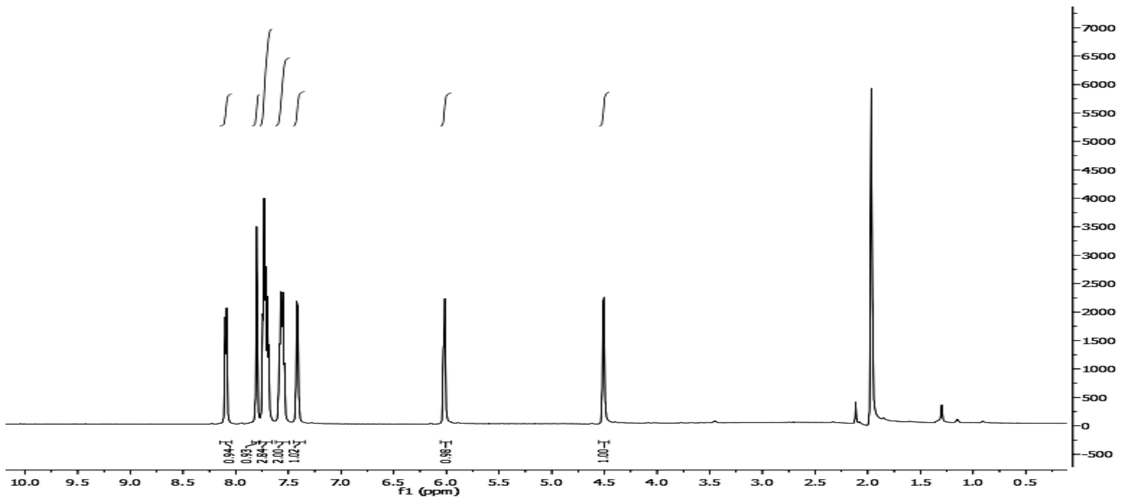

^{1}H NMR spectrum of **27**.

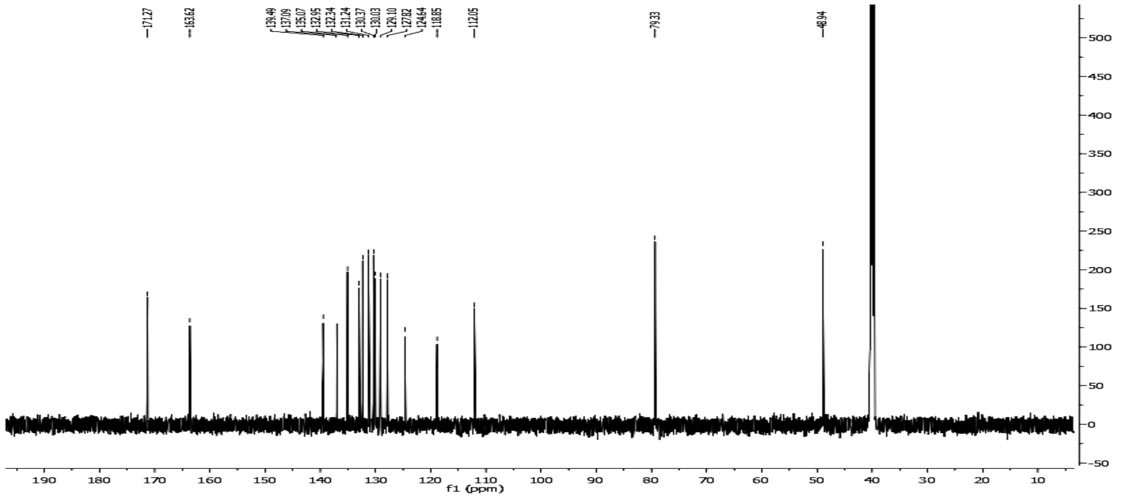

^{13}C NMR spectrum of **27**.

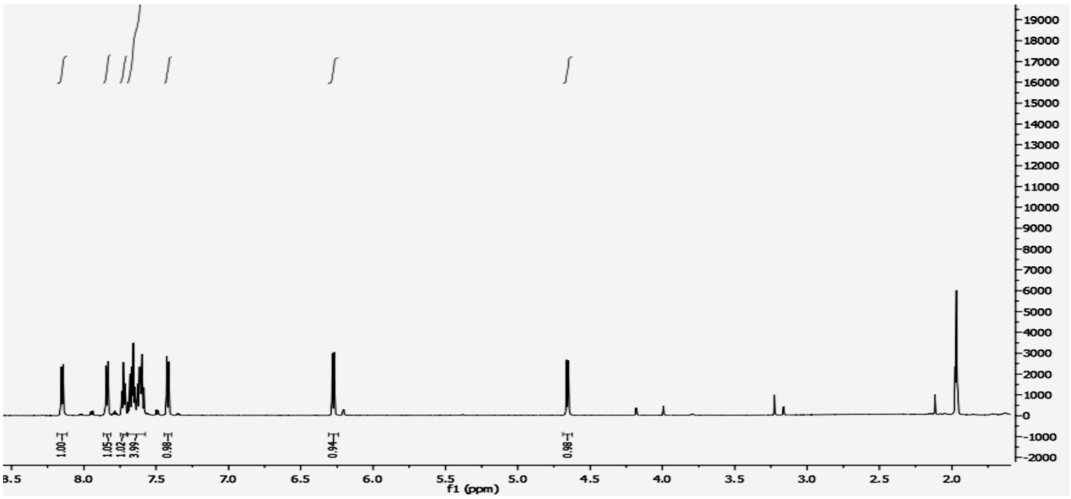

^{1}H NMR spectrum of **28**.

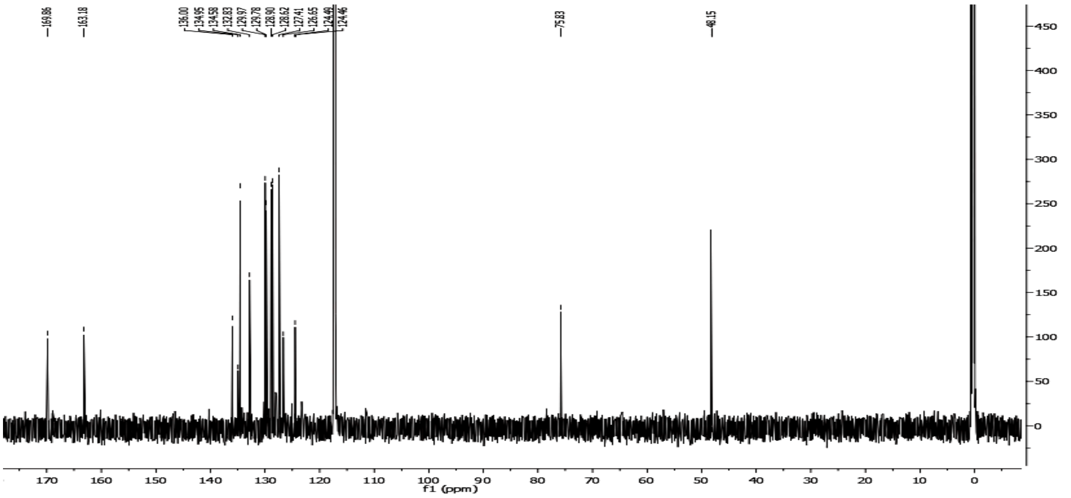

^{13}C NMR spectrum of **28**.

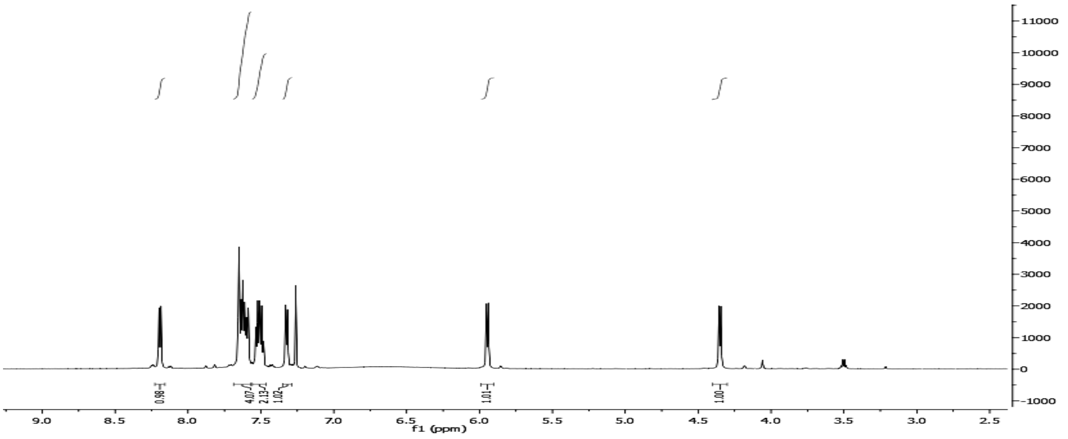

^{1}H NMR spectrum of **29**.

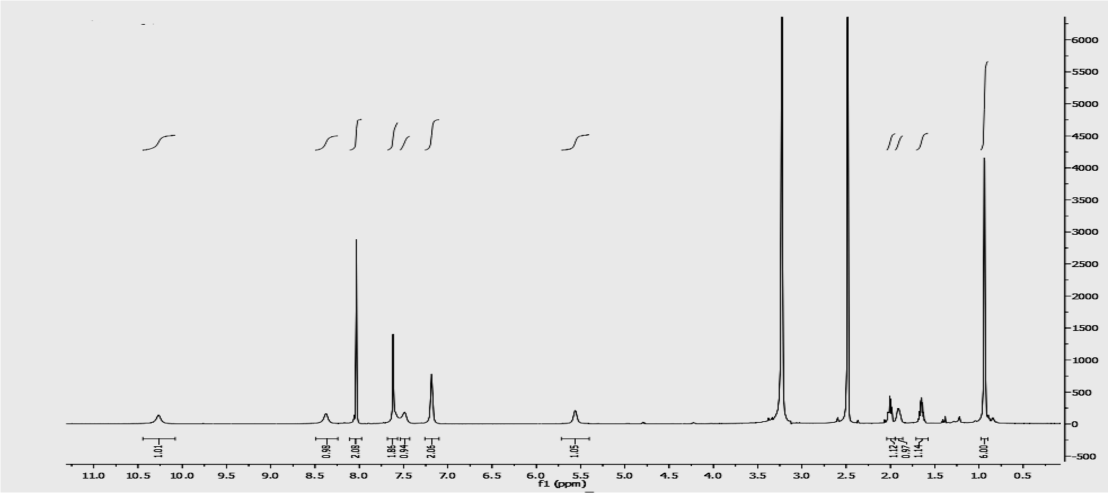

^{1}H NMR spectrum of **24sq**.

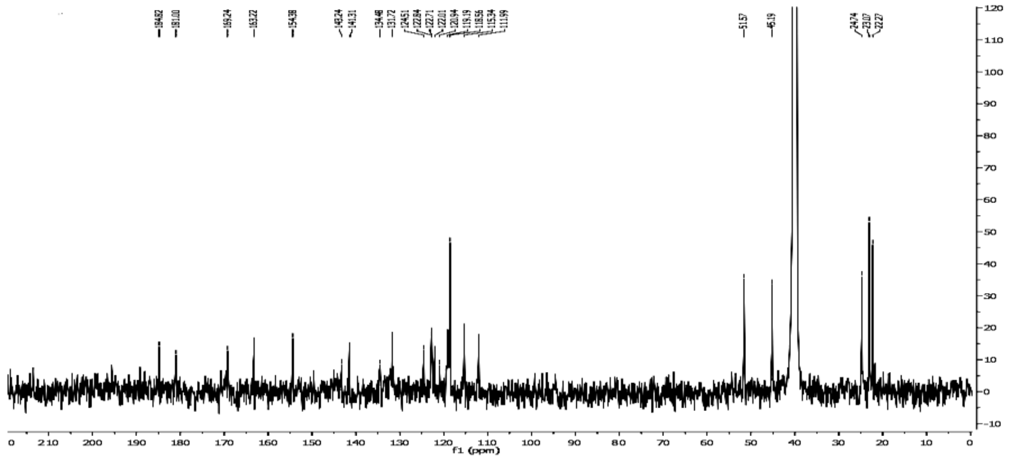

^{13}C NMR spectrum of **24sq**.

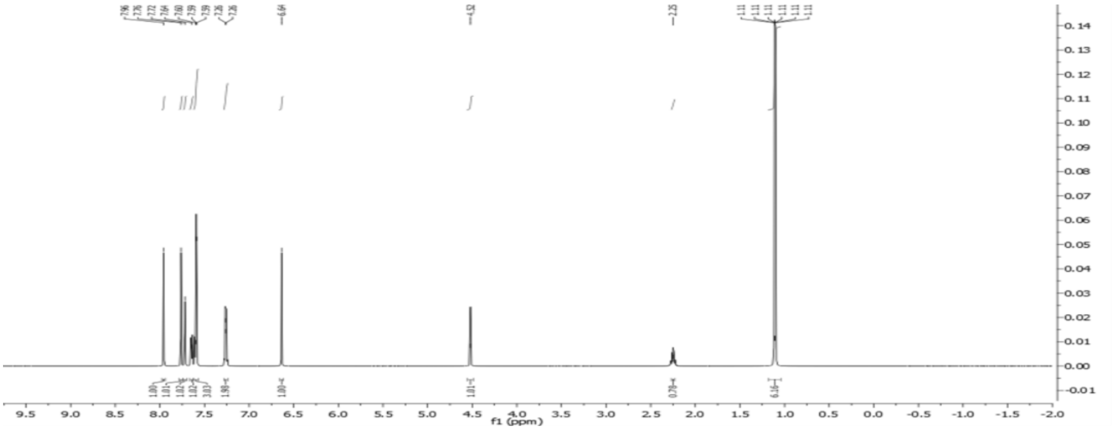

^{1}H NMR spectrum of **26aS**.

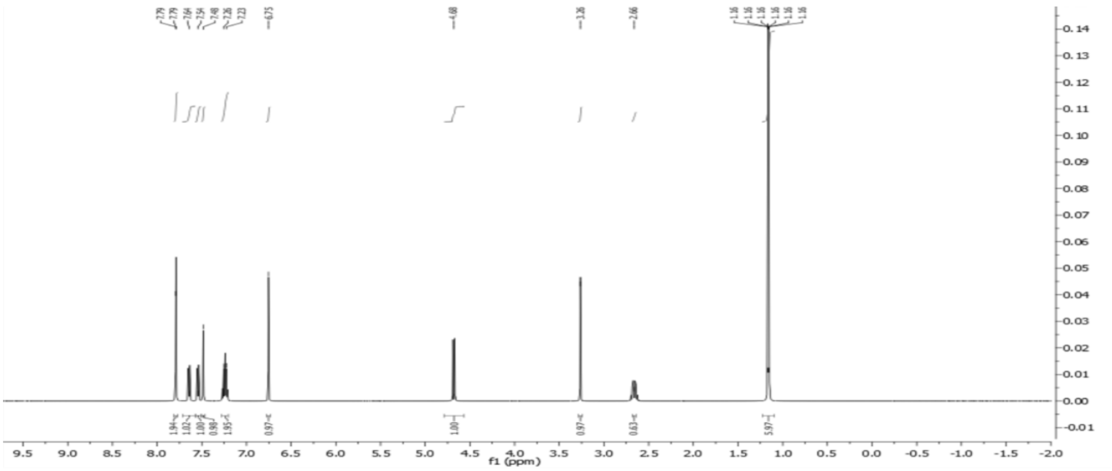

^{1}H NMR spectrum of **26aO**.

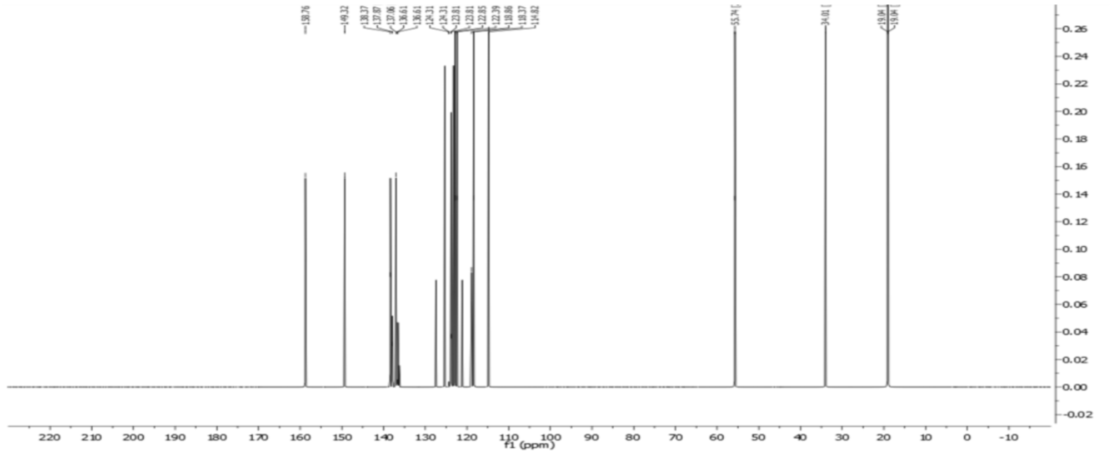

^{13}C NMR spectrum of **26aO**.

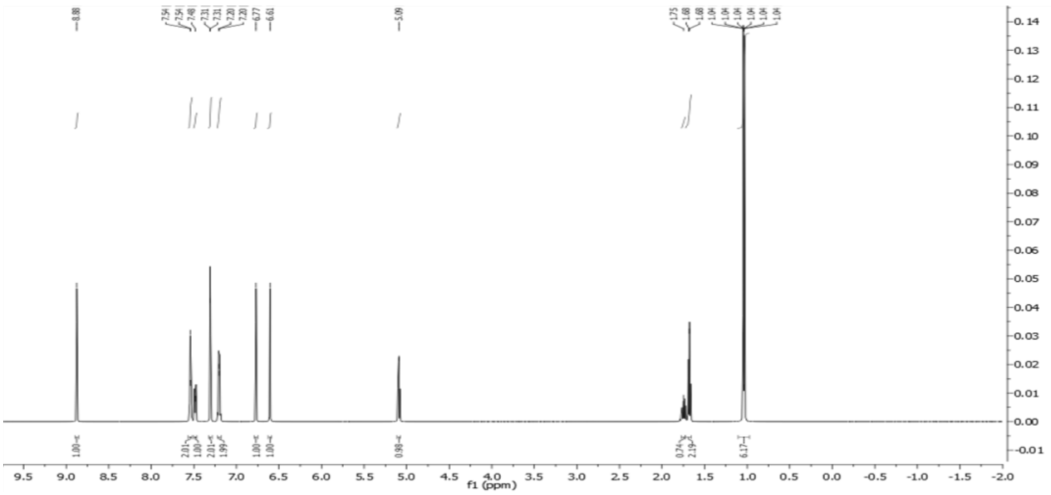

^{1}HNMR spectrum of **25aS**.

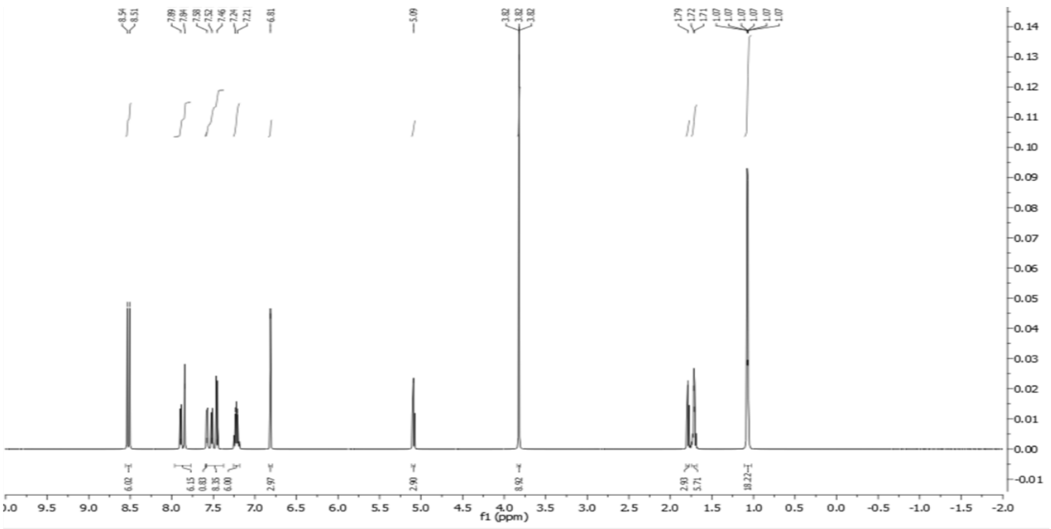

^{1}HNMR spectrum of **25bS**.

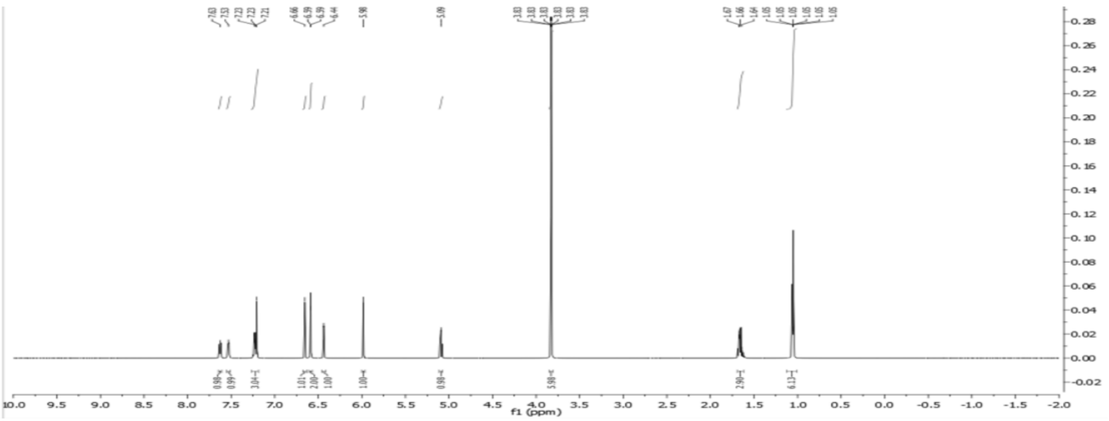

^{1}HNMR spectrum of **25cS**.

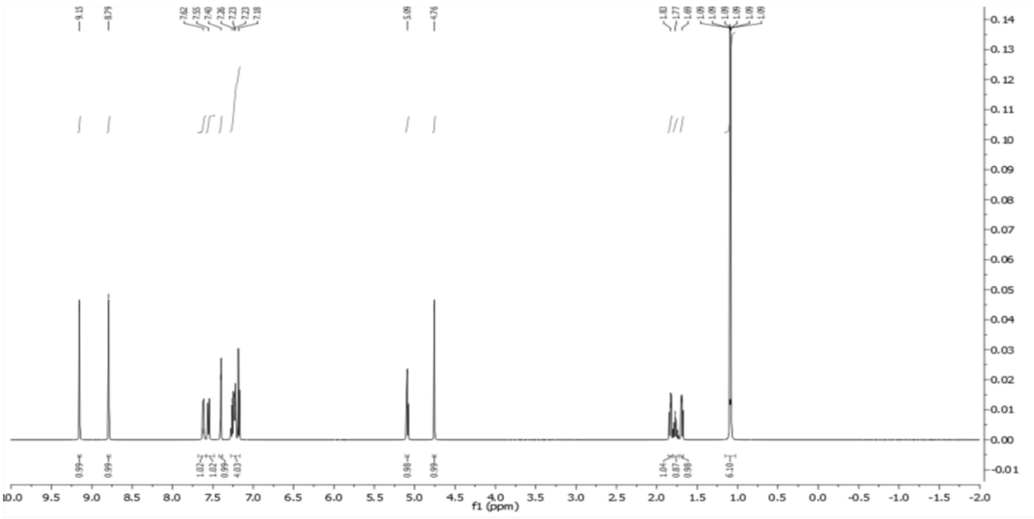

^{1}HNMR spectrum of **25dS**.

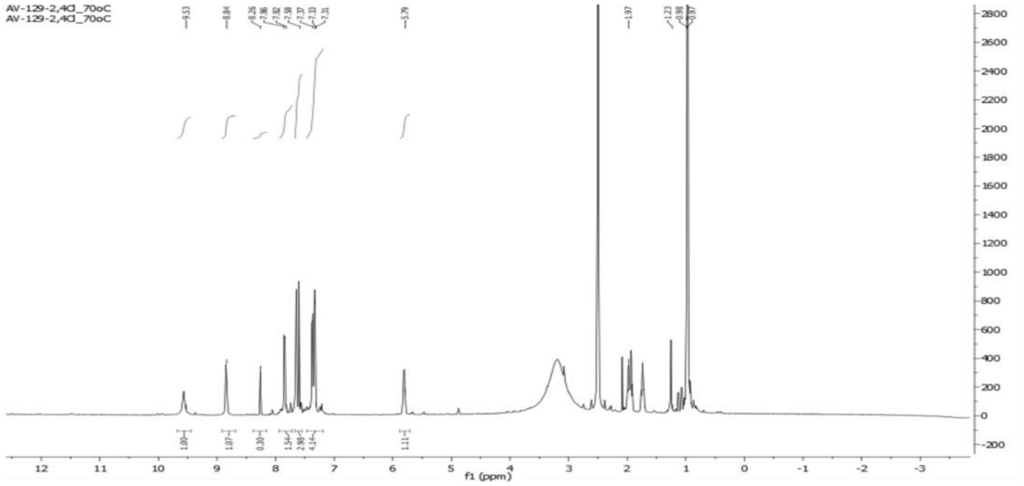

^{1}HNMR spectrum of **25eS**.

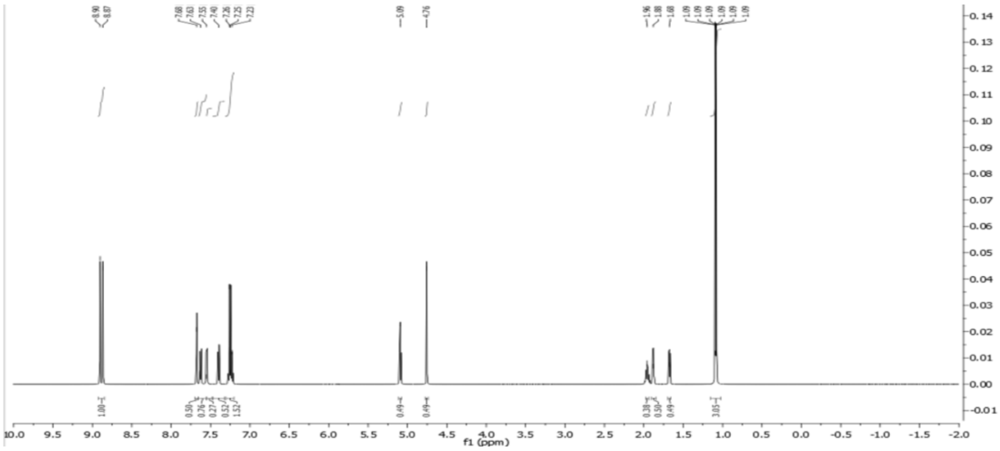

^{1}HNMR spectrum of **25fS**.

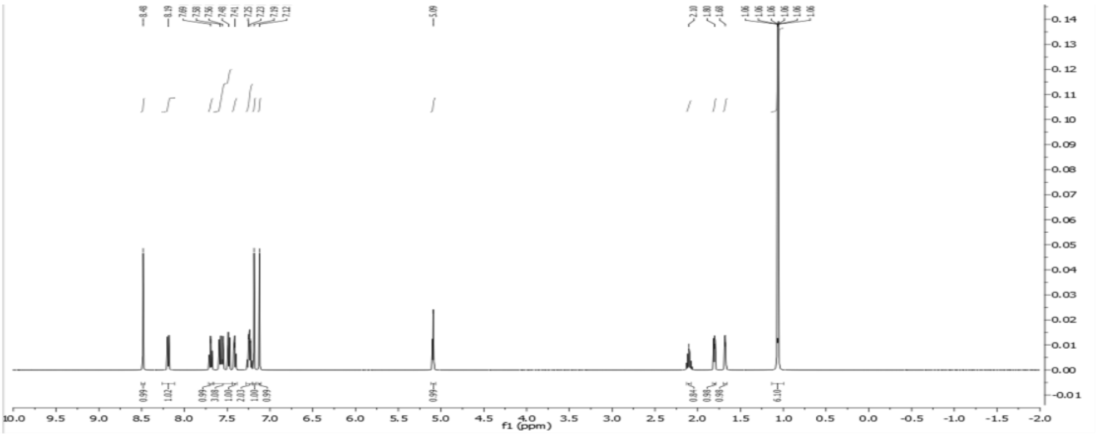

^{1}HNMR spectrum of **25gS**.

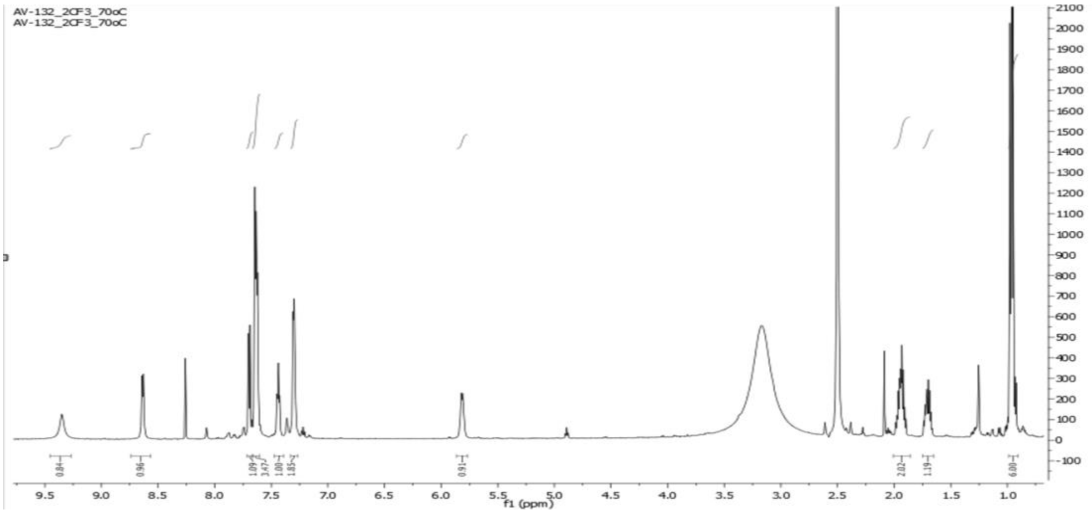

^{1}HNMR spectrum of **25hS**.

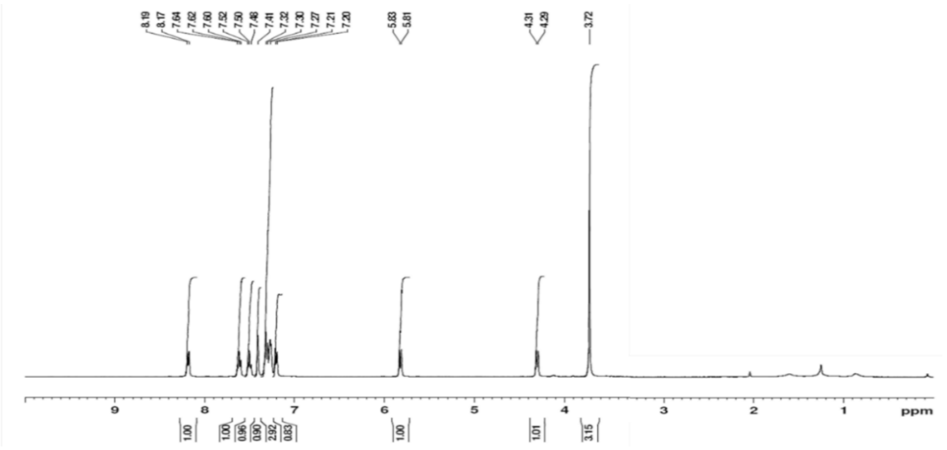

^{1}H NMR spectrum of **39**.

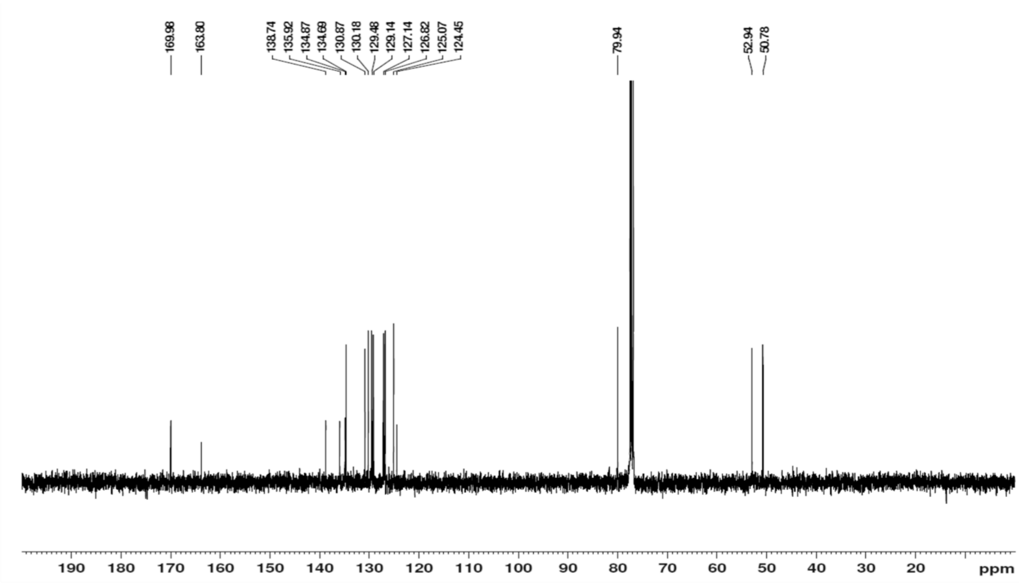

^{13}C NMR spectrum of **39**.

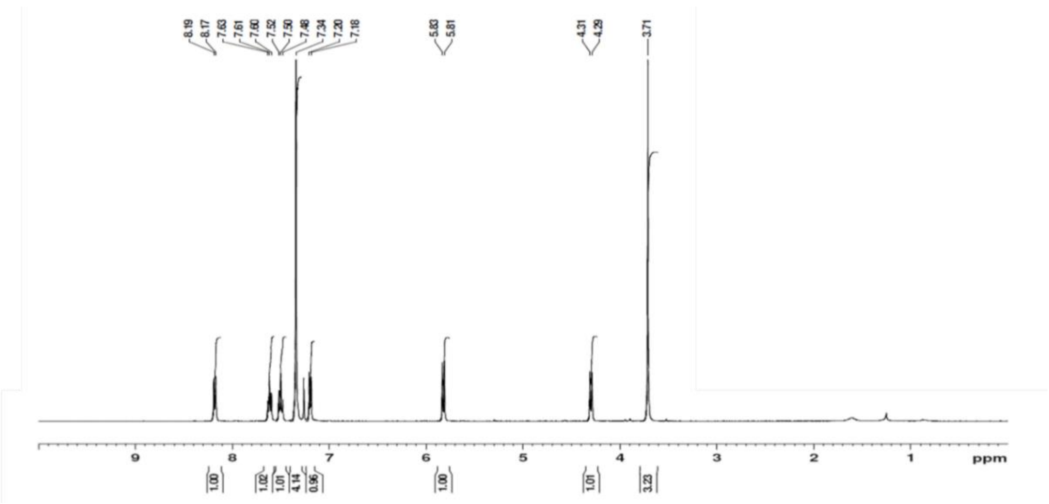

^{1}H NMR spectrum of **40**.

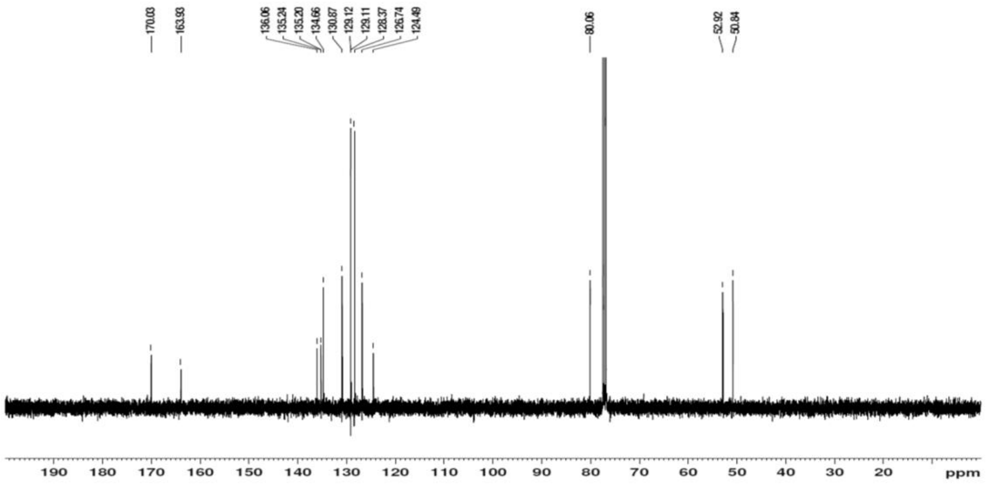

^{13}C NMR spectrum of **40**.

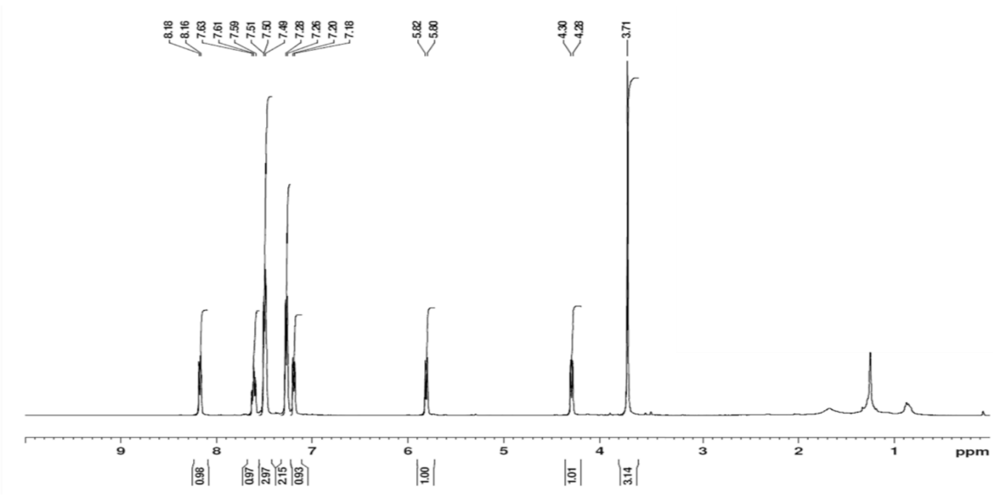

^{1}H NMR spectrum of **41**.

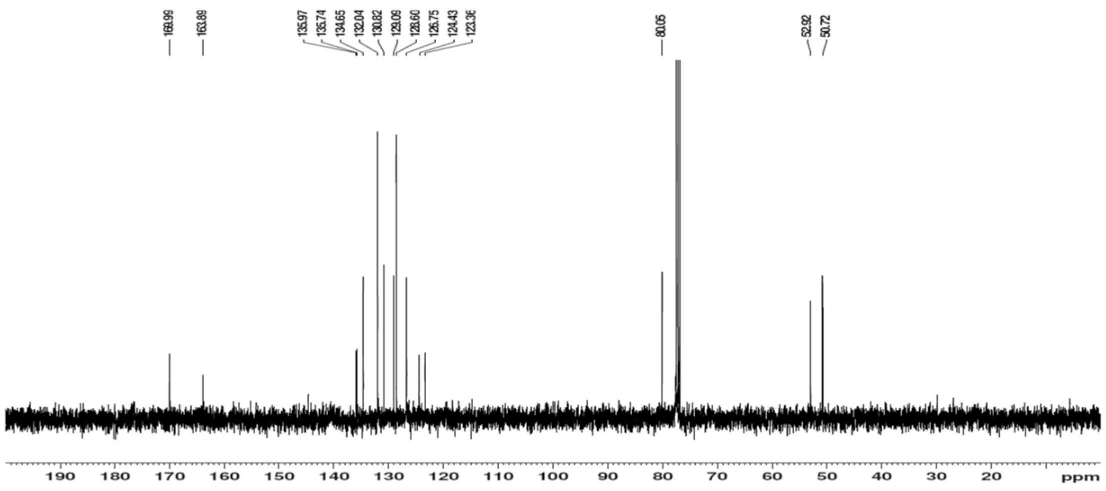

^{13}C NMR spectrum of **41**.

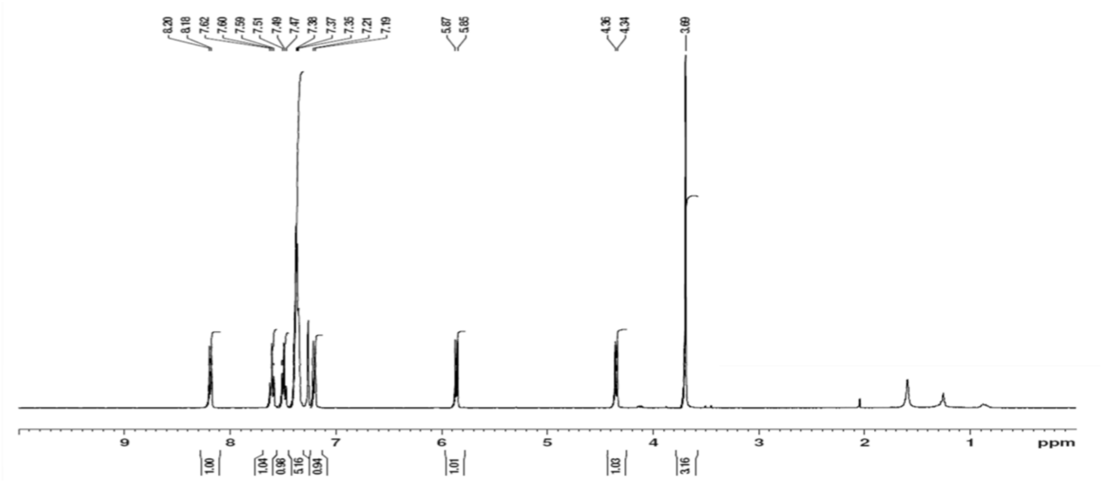

^{1}H NMR spectrumof **42**.

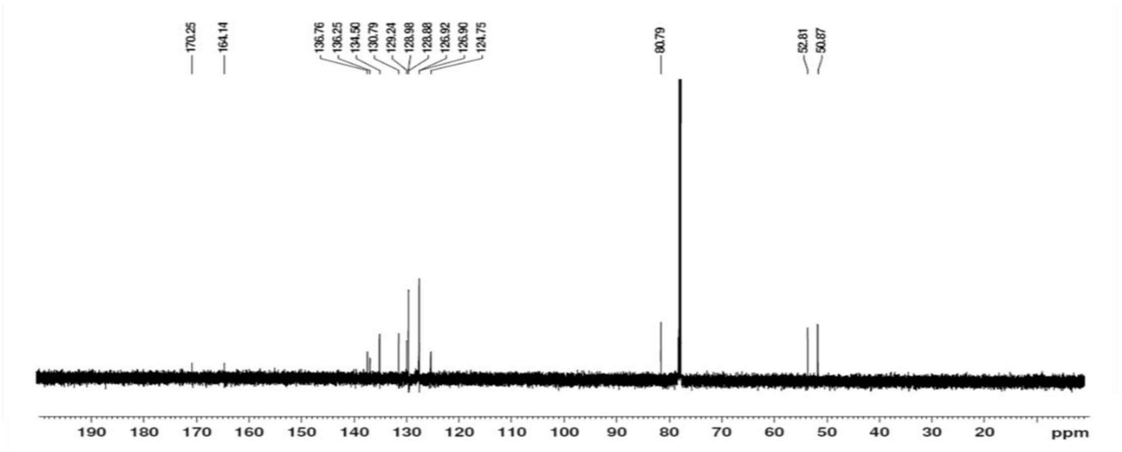

^{13}C NMR spectrum of **42**.

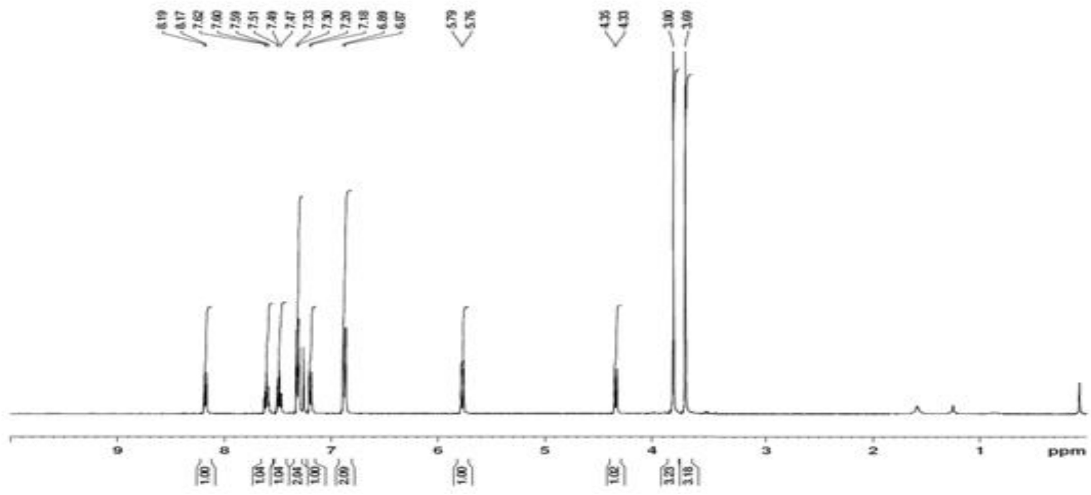

^{1}H NMR spectrum of **43**.

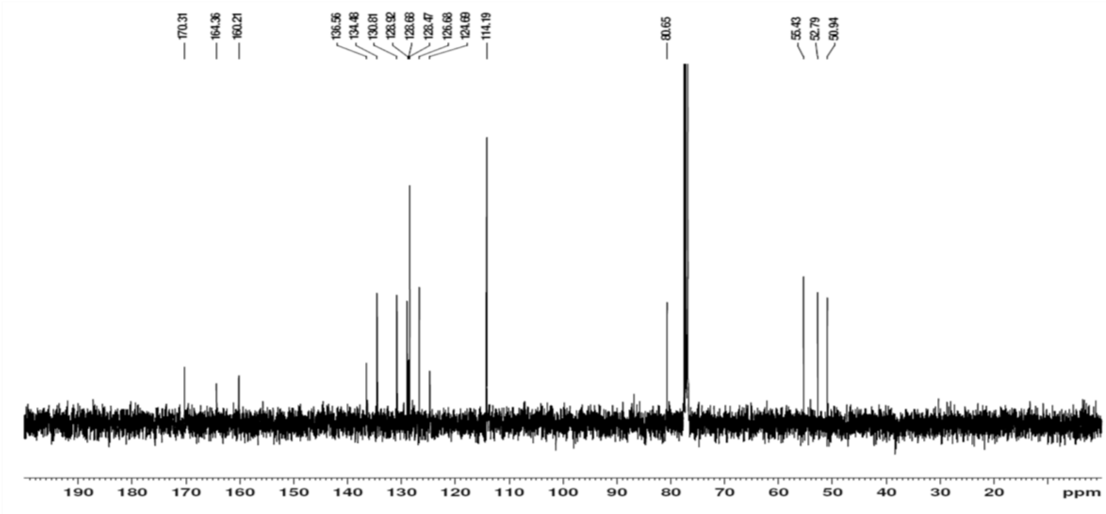

^{13}C NMR spectrum of **43**.

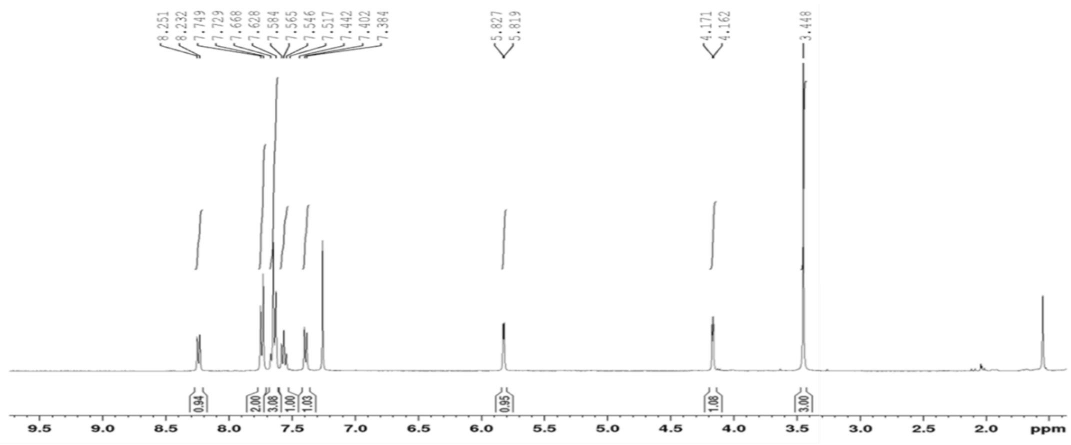

^{1}H NMR spectrum of **44**.

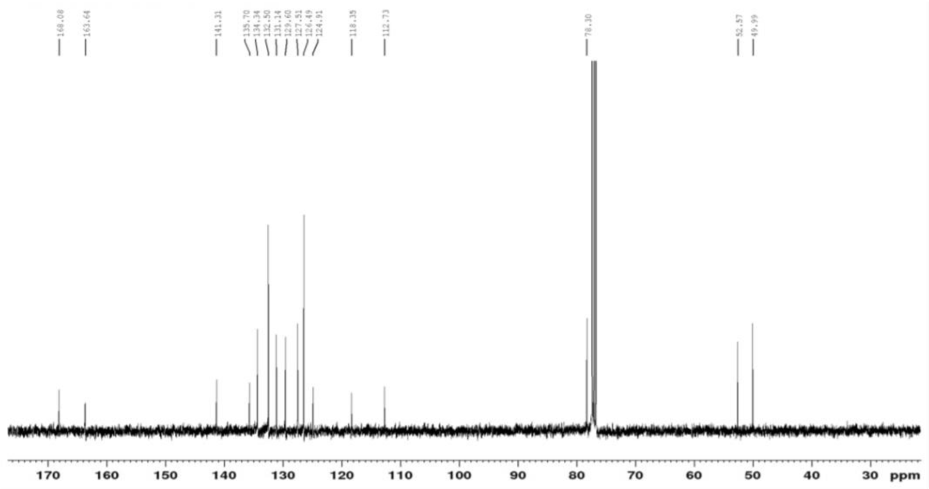

^{13}C NMR spectrum of **44**.

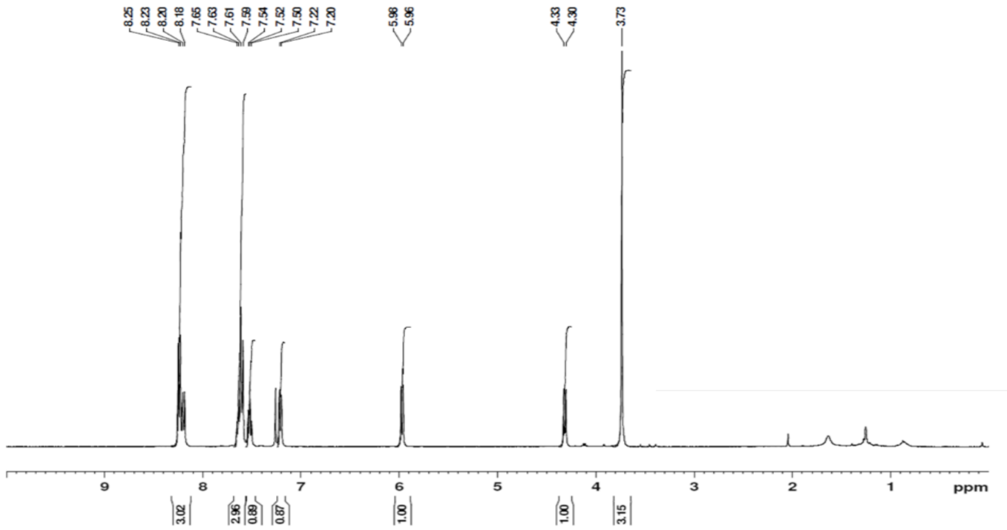

^{1}H NMR spectrum of **45**.

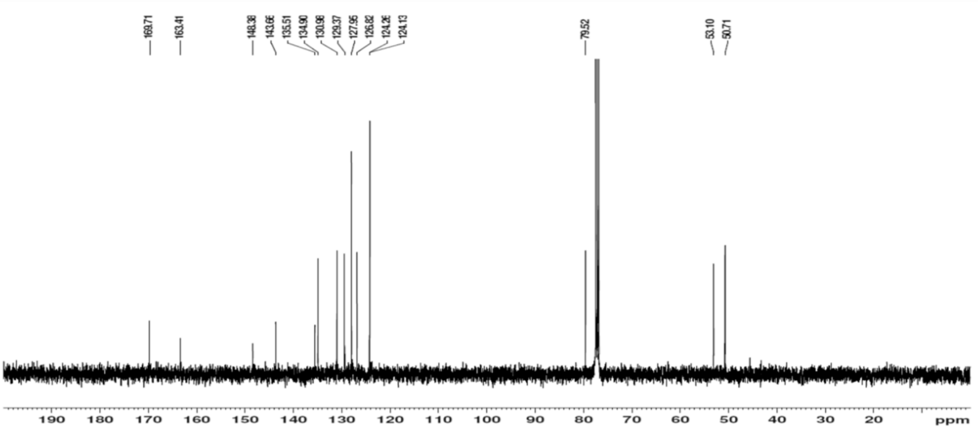

^{13}C NMR spectrum of **45**.
